# Imperfect refractive index matching in scanning laser optical tomography and a method for digital correction

**DOI:** 10.1117/1.JBO.29.6.066004

**Published:** 2024-05-15

**Authors:** Ole Hill, Merve Wollweber, Tobias Biermann, Tammo Ripken, Roland Lachmayer

**Affiliations:** a Leibniz University Hanover, Hannover, Germany; b Laser Zentrum Hannover e.V., Hannover, Germany

**Keywords:** tomography, digital microscopy, scanning laser optical tomography, non-destructive

## Abstract

**Significance:**

Scanning laser optical tomography (SLOT) is a volumetric multi-modal imaging technique that is comparable to optical projection tomography and computer tomography. Image quality is crucially dependent on matching the refractive indexes (RIs) of the sample and surrounding medium, but RI matching often requires some effort and is never perfect.

**Aim:**

Reducing the burden of RI matching between the immersion medium and sample in biomedical imaging is a challenging and interesting task. We aim at implementing a post processing strategy for correcting SLOT measurements that have errors caused by RI mismatch.

**Approach:**

To better understand the problems with poorly matched Ris, simulated SLOT measurements with imperfect RI matching of the sample and medium are performed and presented here. A method to correct distorted measurements was developed and is presented and evaluated. This method is then applied to a sample containing fluorescent polystyrene beads and a sample made of olydimethylsiloxane with embedded fluorescent nanoparticles.

**Results:**

From the simulations, it is evident that measurements with an RI mismatch larger than 0.02 and no correction yield considerably worse results compared to perfectly matched measurements. RI mismatches larger than 0.05 make it almost impossible to resolve finer details and structures. By contrast, the simulations imply that a measurement with an RI mismatch of up to 0.1 can still yield reasonable results if the presented correction method is applied. The experiments validate the simulated results for an RI mismatch of about 0.09.

**Conclusions:**

The method significantly improves the SLOT image quality for samples with imperfectly matched Ris. Although the absolutely best imaging quality will be achieved with perfect RI matching, these results pave the way for imaging in SLOT with RI mismatches while maintaining high image quality.

## 1 Introduction

Refractive index (RI) matching is standard in biological imaging. It allows for distortion free imaging with high magnification. But there can be drawbacks when using immersion media such as oils. They can be expensive, toxic, and environmentally hazardous. If a sample is entirely embedded in an immersion medium, it can also be challenging or impossible to clean it for further use.

Scanning laser optical tomography (SLOT) is a three-dimensional imaging modality that is mostly utilized in biomedical imaging.^1^^–^^3^ It is a method best compared to computed tomography (CT) in which an object is placed in a tube with a detection unit circling around it during image acquisition. Many projections are taken from different incident angles. Volumetric information is generated in a reconstruction process, which usually involves a variation of filtered back projection.^4^ SLOT could be considered a competitor to light sheet fluorescence microscopy (LSFM), which is used in many applications of volumetric fluorescence imaging.^5^ Although SLOT does not reach sub-micrometer resolution, it is possible to image samples in sizes from a few hundred micrometers up to a few centimeters in diameter with a single device and only one objective.^6^ A great advantage of SLOT compared with LSFM, confocal, and multiphoton microscopy is the ability to spatially resolve the absorption of the sample. It is also possible to measure scattering and second harmonic generation,^7^ which is not possible with a typical LSFM.

SLOT has two major differences compared with CT. The contrast mechanism is based on light instead of x-rays, and the sample is rotated instead of the detection unit. Further details and challenges of SLOT are described in section 2. With the use of light instead of x-rays comes the biggest obstacle of SLOT and comparable techniques such as light sheet microscopy. Although x-rays suffer very little refraction when travelling through typical CT media such as human tissue,^8^ light rays are subject to strong refraction when passing media interfaces. This phenomenon is described in one of optics fundamental equations, Snell’s law^9^: $n_{1}*\sin\;\alpha =n_{2}*\sin\;\beta$, where $n_{1}$ and $n_{2}$ are the respective medium’s RI, α is the incident angle, and β is the exit angle. The issue of refraction is typically solved in imaging by surrounding the object with a medium that has the same RI as the sample, e.g., immersion objectives. This can be achieved using the clearing agent used in clearing the object as an immersion medium. An example of this is optical clearing with ethyl cinnamate (ECi) for LSFM in which ECi is used as a clearing agent and the immersion medium.^10^^,^^11^ However, this is only possible for liquid and preferable nontoxic clearing agents. Protocols such as CRISTAL^12^ use a solidified optical adhesive as the clearing agent, which renders them unusable as an immersion medium. In SLOT, it is necessary to fix the object in a transparent and hard material to mount it on a rotary stage for data acquisition. Using the same material as an immersion medium is obviously not possible. Therefore, this issue is usually solved using a silicone oil or a mixture of silicone oils with a closely matched RI. This solution does not come without drawbacks. Silicone oils can be costly, and a large variety of oils is necessary if a wide range of RI’s is supposed to be covered because not all oils are mixable. Further issues concern the toxicity and disposal of silicone oils, which can increase the overall cost further. An approach that reduces the necessity of RI matching is presented in this paper. It allows for data acquisition with imperfect RI matching of the medium and sample by ray propagation simulation and reordering of image data.

## 2 Materials and Methods

### 2.1 Scanning Laser Optical Tomography

SLOT is a digital imaging method usually used for fluorescent biological samples. The technique has similarities with CT, with the main differences being the usage of light instead of x-rays and the rotation of the sample instead of the detection unit. The implications of these differences, especially the refraction of light, are subjects of this work. An explanation of the fundamentals of SLOT is essential to the understanding of the proposed improvements to the method. Therefore, the technique is explained in the following.

The sample is usually a few millimeters in size and needs to be solid to allow for precise and reliable rotation during measurement. Therefore, it is usually embedded in an optical adhesive in a tube-like shape to be mounted on a rotary stage. An optical adhesive is used as the final clearing agent when applying the CRISTAL clearing protocol.^12^ An advantage of this clearing protocol is that the prepared sample is relatively easy to handle because of the hard and transparent tube, which is formed by the optical adhesive. It is also very durable, and the sample is usable for years after clearing. This tube-like shape is necessary for the rotation of the sample during image acquisition. The sample has to be surrounded by a medium with a similar RI because mismatches in RI lead to artifacts and data loss during acquisition. To achieve this, the sample is positioned inside a glass cuvette, which is filled with the immersion medium that is usually a mixture of silicone oils.

**Fig. 1:**
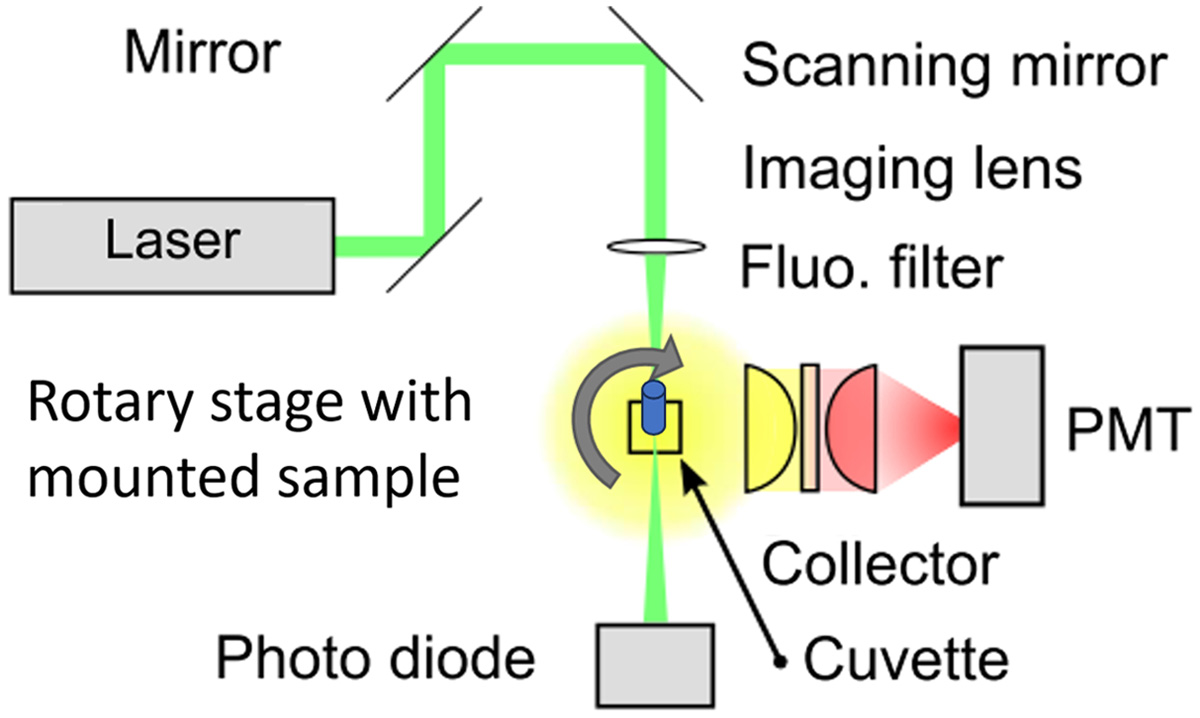
SLOT principle. A laser beam (green) is scanned across a sample, which is mounted on a rotary stage. The sample is embedded in an immersion medium inside a glass cuvette. A photo diode collects the transmitted light while the fluorescent light is focused on a photo multiplier tube, which can be fitted with spectral filters (modified from Ref. 13).

Light is formed in a so-called needle beam, which has a Rayleigh length that is matched to the radius of the sample. The intensity of transmitted, scattered, and fluorescent light is measured in different beam paths, and the laser is scanned over the sample. These data are correlated with the scanner position and result in a projection image. The intensity of transmitted light is measured by a photo diode behind the sample, and the scattered and fluorescent light are measured from the side. The fluorescent light is collected by a lens and filtered through interchangeable spectral filters before being recorded with a photo multiplier tube. The method is schematically depicted in Fig. 1. The resolution perpendicular to the beam is determined by the focusing optic and the resolution along the propagation of the beam is pseudo infinite, which leads to an anisotropic resolution very comparable to the projections of computer tomography. Several hundred projections with equally distributed projection angles are acquired over a full rotation of the sample. The result is a stack of sinograms of the acquired field of view in which the stack size is equal to the number of cross sections that will be reconstructed from the projections. This data can be reconstructed with filtered back projection to form cross sections of the sample containing locally resolved information about transmittance and fluorescence. The volumetric nature of SLOT is achieved by combining the cross sections into a volume as in LSFM. In other words, SLOT is not inherently three-dimensional but rather a method to acquire many neighboring thin cross sections of a sample.^13^

This method works well if the RIs of the sample and medium are perfectly matched. But if there is a mismatch in RI, there is refraction at the boundary of the sample. This leads to a slightly altered real beam path compared to the expected beam path. The supposedly acquired data of each point does not match the actually acquired data. This leads to poor quality of the volumetric data.

### 2.2 Simulation Software

The SLOT simulation software is written in C++ using the Qt GUI library.^14^ Its goal is to visualize the effects of RI mismatches between the sample and the immersion medium in SLOT. To achieve this, ray propagation simulations are performed for each projection angle. The input data consist of two cross sections of a sample in which one only contains the boundary of the sample and the other contains a desired structure or pattern. In the following, the image of the boundary is called the input surface, and the image of the structure is called input structure.

#### 2.2.1 Ray propagation

The incident rays are refracted when hitting an interface of changing RI, e.g., the sample surface. Snell’s law is applied in this simulation. The RIs of the sample and medium can be set to any value between 1 and 99. The slope of the surface at each incident point is determined by polynomial regression with a variable order and variable amount of considered neighbors. The exit angle represents the divergence from the refraction-free beam path. It is calculated with simple trigonometric functions.

The ray is then propagated through the cross section of the input structure. It is assumed that the sample has a constant RI and that the rays are not refracted inside the sample. A bi-linear interpolation of the four closest neighboring pixels of the in-between-pixel position of the ray after it propagated the distance of one pixel is performed repetitively until the ray hits the surface on the backside. The values of these bi-linear interpolations are summed to make up the brightness of the pixel of the incident position of the ray in the projection image. This propagation is repeated for each incident position of each projection angle and results in a sinogram of the input structure.

The deflection at the exit point in the specimen is not considered for this work. The fluorescent light, which is the main information to be measured, is unidirectional and collected by a photo multiplier tube underneath the glass cuvette. The deflection of the illumination beam at the back of the specimen is therefore irrelevant for the fluorescent light. The measurement of the transmitted light is influenced by the deflection at the back end of the specimen. This could lead to a total loss of light when the accumulated deflection is strong enough that no light hits the photo diode, which measures the transmission intensity. This is undesirable and remains a challenge to be overcome. Moving the photo diode closer to the glass cuvette would increase the acceptance angle and reduce the effect of strong deflection on the transmitted light intensity.

#### 2.2.2 Rearrangement of sinogram

**Fig. 2:**
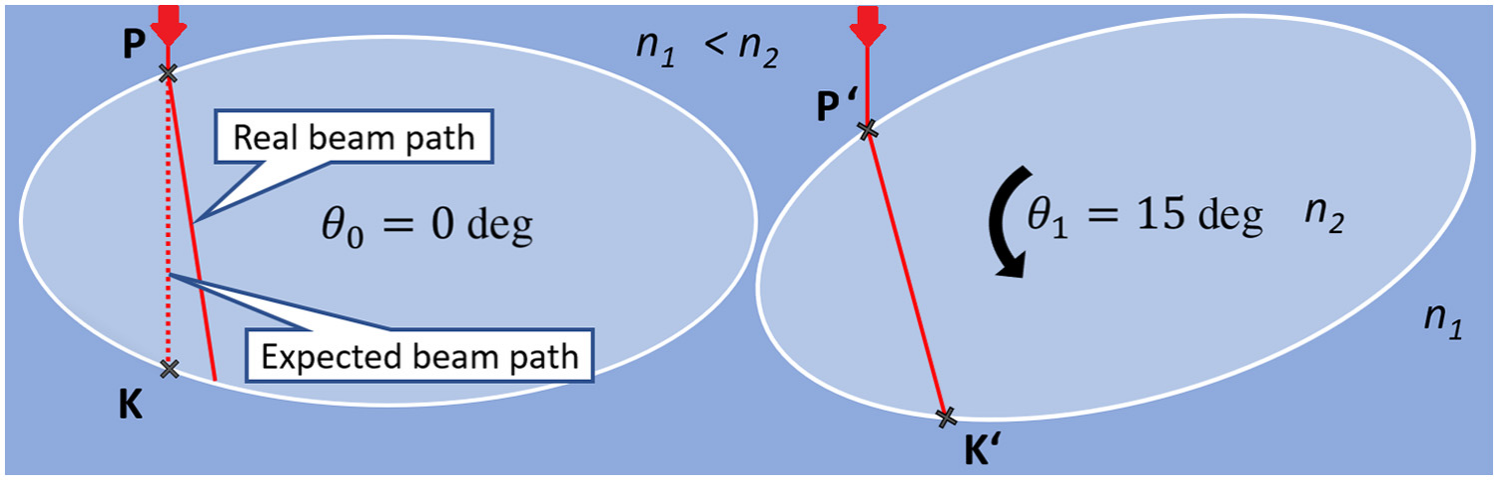
Refraction during RI mismatched SLOT measurement. Dark blue: immersion medium; light blue: specimen. Left: the ray at entryPoint P is refracted because the RI of the medium does not match the RI of the sample. Because of that, the real beam path (solid red line; RBP) is unequal to the expected beam path (dotted red line; EBP) and subsequently incorrect information is measured. Right: after digitally rotating the sample by Δθ, the RBP at $\theta_{1}$ (arbitrary) is equal to the EBP at $\theta_{0}$. This means that the information that was supposed to be recorded at entryPoint P was actually recorded at $P^{\prime }$ at a different rotation angle ($\theta_{1}$). In other words, $\overset{\to }{PK}$ contains the same information as $\overset{\to }{P^{\prime }K^{\prime }}$.^17^

Because the sample is rotated during data acquisition, there is information from every incident angle (projection angle) for each surface position. All of this data is saved in the sinogram of the input structure. When the RI is not matched, this information is simply associated to the wrong position.^15^^,^^16^ The goal of the rearrangement software is to correct these positioning errors by searching for the matching surface position and projection angle. The ray path through the sample when refraction occurs can be translated into a path without refraction at a different rotation angle of the sample. Fig. 2 visualizes the problem and the proposed solution. By digitally rotating the sample utilizing the OpenCV library,^18^ the projection angle is found; the incident position is hit by the ray in a way that the ray travels vertically through the sample at the original projection angle of the sample.

**Fig. 3:**
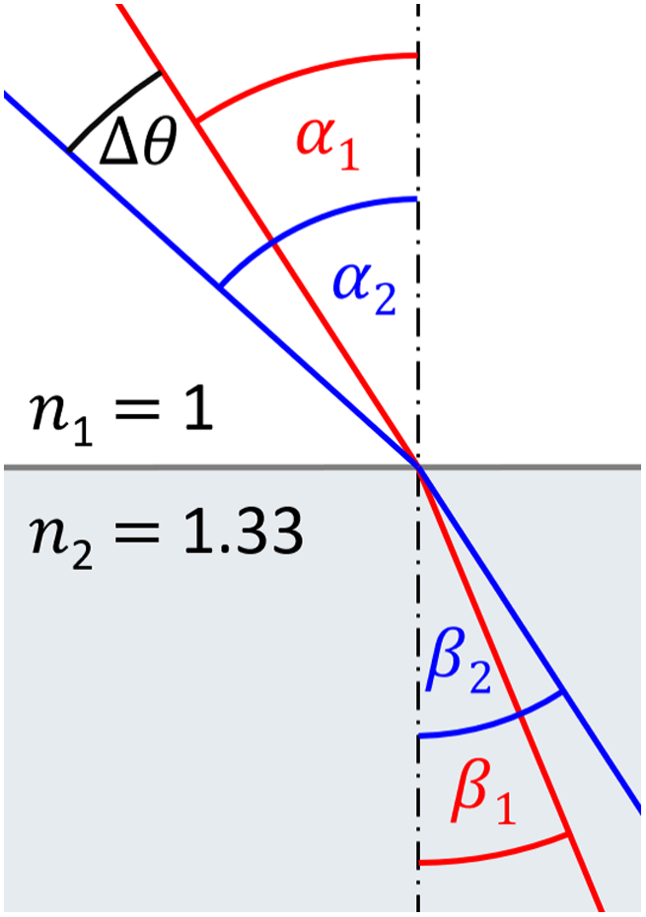
Sketch to show the derivation of the equation to determine how much steeper or flatter the incident angle has to be to get an exit angle as if no refraction occurred. The values are arbitrary and just for illustration.

A preemptive surface is generated by reconstructing the uncorrected transmission measurement. Although the sinogram is potentially distorted and yields no useful information about the inside structure of the specimen, the shape (or surface) of the specimen is always reconstruction correctly. The boundary of the specimen is visible in SLOT in the transmission even when the specimen is optically cleared. The surface roughness of a real specimen is responsible for some absorption or deflection, and this leads to the visibility of the boundary of the cleared specimen. Before a rearrangement of the sinogram is possible, each ray’s refraction angle for each projection angle has to be calculated. This is done by rotating the input surface to each projection angle and performing ray propagation calculations. This information is temporally saved in a multidimensional list called entryPoints. In the next step, Δθ (compare Fig. 2) is calculated for each incident point and angle with the following equation (the derivation is explained in Eqs. 2(a)–2(d) and in Fig. 3)

$$\Delta \theta =\arcsin[\frac{n_{2}}{n_{1}}*\;\sin(\alpha_{1})]-\alpha_{1},$$ (1)

where Δθ is the angle by which the sample has to be digitally rotated to get the data of the real beam path, $n_{1}$ is the RI of the medium, $n_{2}$ is the RI of the sample, and $\alpha_{1}$ is the incident angle of the laser beam. Δθ is converted to an offset in the sinogram, where each horizontal line represents a projection. 800 projections over a 360 deg rotation is a typical angular sampling rate in SLOT, which means that a Δθ of one degree equals a vertical pixel offset of $2.\overset{¯}{2}$ in the sinogram. The vertical offset in the sinogram is calculated by multiplying this factor by Δθ. As the sample rotates, the position of an incident point also rotates. A horizontal offset is calculated to account for this. The incident position is known from the ray propagation calculations, which are performed prior to the rearrangement of the sinogram. The incident position is rotated around the center of rotation by Δθ, which yields the horizontal offset of the incident point. The horizontal and vertical offsets are added to the original position in the sinogram, and bilinear interpolation is performed to acquire the brightness value for the rearranged sinogram.

Δθ is computed as (compare Fig. 3)

$$\Delta \theta =\alpha_{2}-\alpha_{1}.$$ (2a)

The exit angle is equal to incident angle:

$$\alpha_{1}=\beta_{2}.$$ (2b)

Snell’s Law is used to solve for $\alpha_{2}$ as

$$\alpha_{2}=\arcsin(\frac{n_{1}}{n_{2}}*\sin\;\beta_{2}).$$ (2c)

Inserting Eqs. (2b) into (2c) and Eqs. (2c) into (2a) results in

$$\Delta \theta =\arcsin(\frac{n_{1}}{n_{2}}*\;\sin\;\alpha_{1})-\alpha_{1}.$$ (2d)

The RI of the specimen has to be relatively homogeneous. Refraction inside the specimen cannot be corrected by the presented method. Although this is unfortunate, it should be noted that refraction inside a specimen also reduces the performance of every other optical imaging method. A sufficiently cleared biological specimen (which has to be assumed to have a homogeneous RI distribution) or a technical sample, such as the polydimethylsiloxan (PDMS) waveguides used as an example in this work, is needed for SLOT.

To derive an RI of a specimen that is not exactly known, a method is implemented into the correction software to allow for the correction of a sinogram with different specimen RIs. The user can compare the image quality of the resulting reconstructions to estimate the RI of the specimen. Some preemptive knowledge of the expected structures inside the specimen and user experience is necessary for this.

#### 2.2.3 Reconstruction

The tomograms (or cross sections) were reconstructed utilizing filtered back projection,^4^^,^^19^ which is a standard method for the reconstruction of a volume from projections in CT. Mathematically, it is based on the inverse Radon transform, which was published over 100 years ago.^20^ Fiji,^21^ which is a library for ImageJ,^22^ and the IMOD^23^ library, which is an open source library for tomographic reconstruction, were used in this process. The tool for reconstruction was written specifically for SLOT but works for any set of projections that are acquired in a parallel projection setup.

#### 2.2.4 Artificial dataset

**Fig. 4:**
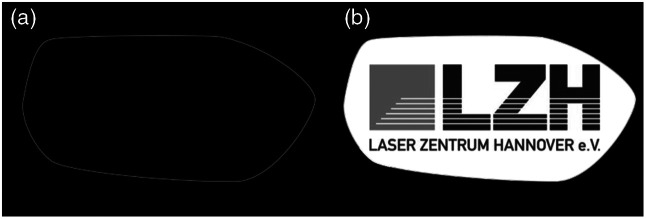
Input surface (a) and input structure (b) used for the simulated results (Sec. 3). 8-bit gray value format with a resolution of 650×688  pixels each.^17^

SLOT requires the sample to be embedded in an optical adhesive to mount the sample on the rotary stage. Although this compound can, in theory, be of any geometrical or random shape, the practice in the lab is standardized. To execute the CRISTAL clearing protocol successfully, the sample and optical adhesive are put in a thin Teflon tube for curing. This results in a cylindrical shaped compound with a constant RI (Fig. 4).

A hand drawn shape was used as the digital phantom outline with this fact in mind. But to account for potential changes in radius and curvature of a real compound, the phantom is not perfectly round but rather potato shaped. The phantom is two-dimensional because of reasons discussed in Sec. 2.1. In short, SLOT is not truly three-dimensional but spatial stacking of thin optical cross sections through precise imaging planes. If the outer boundary of the compound in the vertical direction (along the rotary axis) is not close to perpendicular to the incident beam, the presented correction method fails. This is because filtered back projection only works for parallel beam geometries and a precise two-dimensional image plane. If the outer boundary in the vertical direction is not close to ninety degrees and there is an RI mismatch, the laser beam gets refracted away from the precise imaging plane, and this derivation cannot be corrected digitally. Therefore, only a two-dimensional phantom is considered in this work.

The inside of the digital phantom is the logo of the Laser Zentrum Hannover e.V., where bright parts represent a high fluorescence signal. The phantom contains large homogeneous areas as well as very small and precise structures, e.g., the letters in the lower half. The input surface and input structure are illustrated in Fig. 4.

### 2.3 Real Specimen: Agarose Cylinder with Fluorescent Polystyrene Beads

A cylinder with an agarose (UltraPure Low Melting Point Agarose, Thermo Fisher Scientific, United States) concentration of 1% was prepared as an exemplary sample. Fluorescent polystyrene beads (FluoSpheres orange (F8833), Thermo Fisher Scientific, United States) with a concentration of $1.2*10^{4}$ beads per milliliter were added. The water-agarose-bead-emulsion was heated to 60°C and drawn into a syringe with a diameter of 3 mm, where it cooled and cured. The agarose cylinder was partially pressed out of the syringe, so it could be imaged directly. Negative pressure held the agarose cylinder in place (pressed out the syringe by a few mm) and the syringe was mounted on the rotary stage for the SLOT measurement.

Agarose was chosen for the test sample because the fluorescent beads are immersed in water and agarose is easy to prepare for SLOT using a syringe. Matching the RI with water was also very convenient as the agarose cylinder has an RI of about 1.335,^24^ an RI mismatch to water, which leads to weak enough refraction that it can be neglected for SLOT.

### 2.4 Real Specimen: Polydimethylsiloxan Cylinder with Fluorescent Nanoparticles

A second exemplary specimen consists of a polydimethylsiloxan (PDMS, Dowsil™ EI-1184) cylinder with supposedly equally distributed fluorescent nanoparticles. It was manufactured in an embedded printing process. Two-component PDMS is mixed and extruded into a matrix gel in which it cures for several hours. One component is enriched with fluorescent nanoparticles prior to printing. The RI of the used PDMS is 1.42.

### 2.5 SLOT Measurement of Agarose Cylinder

The agarose cylinder was measured in two different immersion media. Distilled water was selected as the immersion medium to display a case in which the RI fits reasonably well. The difference in RI of 0.002 at 1% concentration of agarose and water was considered negligible for the scope of this work. This conclusion was drawn from the relation between the concentration of agarose and the resulting RI of the agarose gel.^24^ A silicone oil with an RI of 1.422 (AS 100, Sigma-Aldrich, Germany) was selected as the second immersion medium to display the consequences of poorly matched RIs and test whether the correction algorithm presented in this work performs well in a real measurement. Although it would not make sense to use a mismatched silicone oil when water is matching the RI very well, this setup was chosen purely to test whether the correction algorithm works for a real measurement.

A laser diode with a wavelength of 532 nm was used for the measurements of the agarose cylinder. A 590 nm longpass filter was used to filter the fluorescence light, and 800 projection images were acquired during a 360 deg rotation. The resolution of the reconstructed cross sections, which in SLOT is always dependent on the sample diameter, is about 10  μm in all three dimensions.

### 2.6 SLOT Measurement of Polydimethylsiloxan Cylinder

The SLOT measurement settings were equal to the ones mentioned above. Water was used as the immersion medium for the mismatched case, and the silicone oil mentioned above was used to measure the PDMS cylinder with a matched RI. It should be noted that the PDMS cylinder interacted with the silicone oil. A swelling of up to 10 percent in each dimension was observed. Because this process takes about 2 days before it reaches saturation, the cylinder rested in silicone oil for 2 days prior to imaging. The geometry and stiffness of the sample changed because of the interaction with the silicone oil.

### 2.7 Quantitative Analysis

In an effort to evaluate the simulated results, two methods of quantification were applied. The first method is known as structural similarity index measure (SSIM).^25^ An SSIM plugin for ImageJ was used.^26^ It is used to quantify how similar images appear to human observers. The evaluation goes from zero, which would be a total mismatch between the images, to one, which would mean absolute similarity. The second method is called absolute pixel difference (APD) in the following. For APD, the difference in brightness of each pixel of the result compared to the expected image is taken, and the average over the image or region of interested is calculated. Here, a value of zero means a perfect match. The comparison between a white and black image would yield the highest APD value possible, which is 255.

**Fig. 5:**
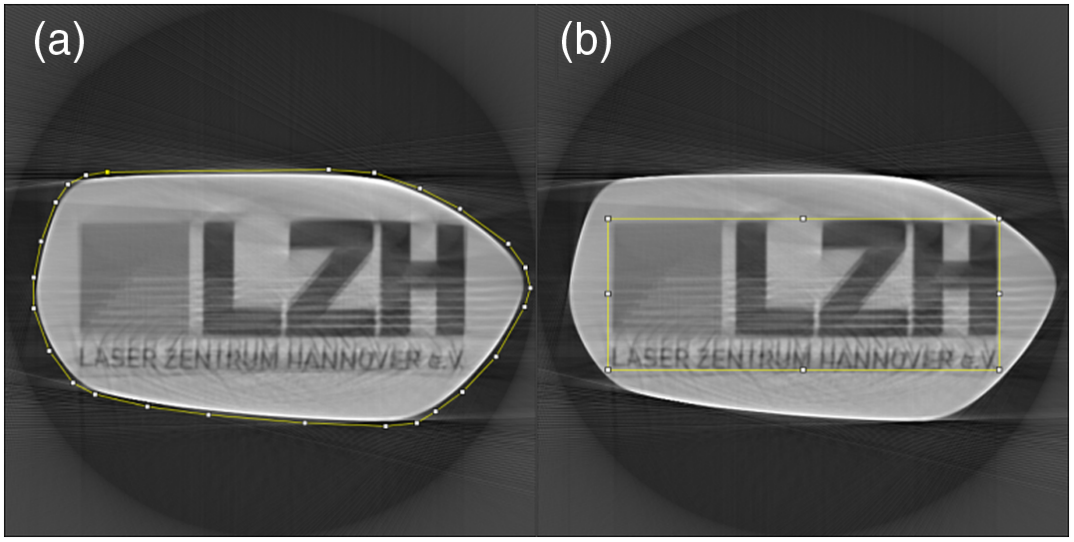
Two different regions of interested (ROI) were evaluated for each simulation. (a) The larger ROI, which includes the entire cross section and a bit of background. (b) The smaller ROI, which only includes the LZH logo. The typical ring artifact of tomograms is visible on the outside of the images.^17^

The outer parts of tomographic images reconstructed with filtered back projection are often polluted with artifacts and contain no useful information. Because of this, both methods were applied to the object area and a few pixels around it. A second region of interest was set centrally in the object. Both areas are shown in Fig. 5.

## 3 Results

### 3.1 Simulations

The evaluation of the results of the simulations is split up into qualitative and quantitative analysis. It is important to make this distinction as different applications can have different requirements. For example, if a classification of tissue is done by a person, the structural similarity might be more important than the numerical similarity, e.g., contrast. But if a numerical analysis is performed, the brightness of individual pixels might be the deciding factor.

#### 3.1.1 Qualitative analysis

**Fig. 6:**
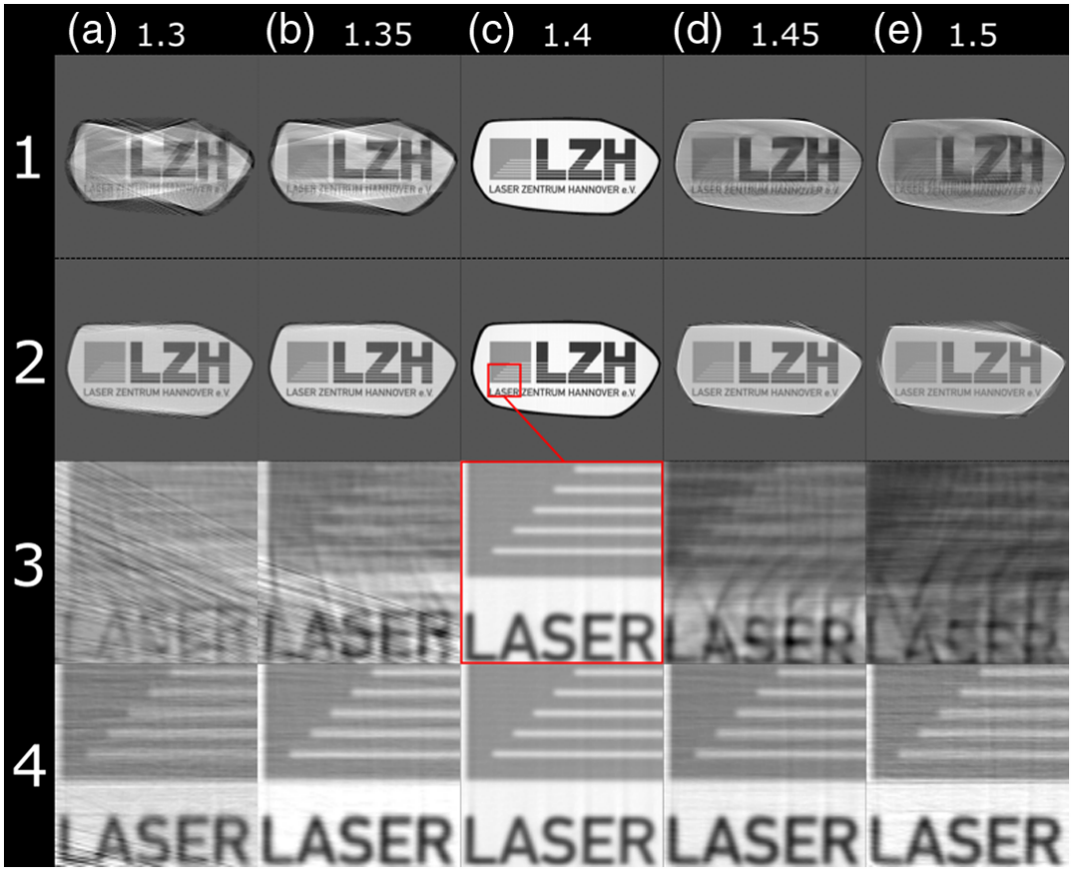
(1a)–(4e) Comparison of reconstructions of different RI mismatches: the numeric values on top indicate the RI of the sample. The RI of the medium was 1.4 in all cases. Rows 1 and 3 show reconstructions without correction of the sinogram. Rows 2 and 4 show reconstructions with the sinogram corrected for refraction. Rows 3 and 4 display zoomed-in images from rows 1 and 2, respectively. 800 projections over one rotation were simulated for these images.^17^

Figure 6 shows reconstructions of simulations of the digital phantom with different mismatches between the medium and sample. The RI of the medium was 1.4 in all cases. 800 projections were performed for each result. Rows 1 and 3 show the results without correction, and row 2 and 4 show results in which a rearrangement of the sinogram based on ray propagation was performed. The lower two rows are zoomed-in images of the upper ones, indicated by the red square. Column (c) shows the result of reconstruction when no RI mismatch is present; the contrast in these images is the highest of all columns, and the structure is best resolved.

The sample appears smaller in the reconstruction in Figs. 6(1a) and 6(1b) than in the matched case displayed in Fig. 6(1c). This effect is called shrinking in the following. The opposite effect is visible in Figs. 6(1d) and 6(1e), where the inner part of the sample appears enlarged. There is no shrinking or enlargement visible in Fig. 6 row 2, where the sinograms were rearranged prior to reconstruction. A few line-shaped artifacts are visible in Fig. 6(4a), and the correctly reconstructed area is reduced in Figs. 6(2d) and 6(2e). In these cases, information is lost at the edges of the phantom.

#### 3.1.2 Quantitative analysis

**Fig. 7:**
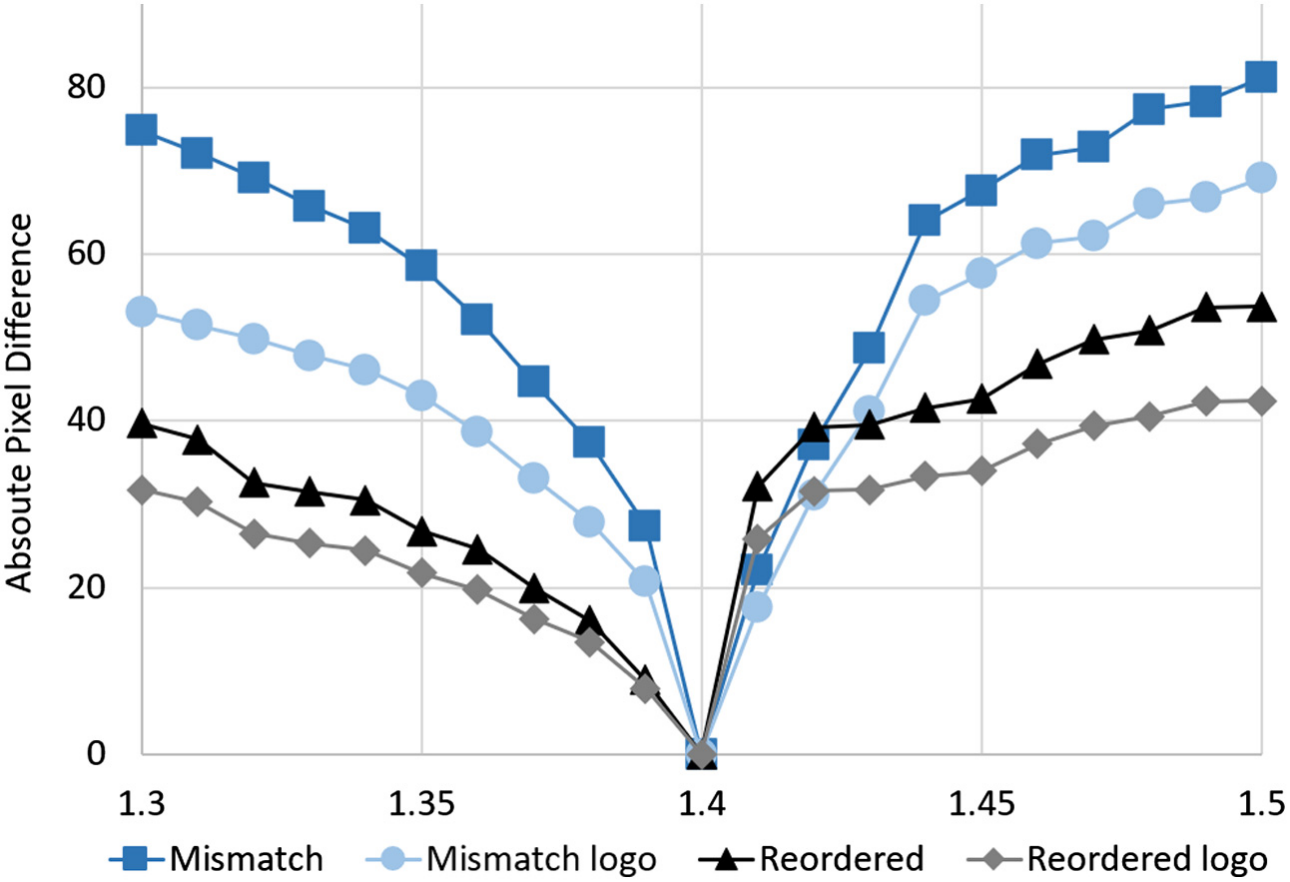
APD analysis of simulated reconstructions. The sample RI is indicated on the horizontal axis. The RI of the medium is 1.4 in all cases. The averaged APD over the respective region of interest is shown. The ground truth image as well as the different regions of interest are depicted in Figs. 4 and 5.^17^

Figure 7 shows the results of APD applied to the images shown in Fig. 6 and additional simulations complementing the shown results with an increment of 0.01 mismatch in RI. The lower the value is, the higher the similarity to the result in which no RI mismatch was present is. The subjective improvement of the result shown in the previous section when the sinogram is rearranged is quantitatively confirmed here. Going back to the qualitative analysis, it can be seen that the corrected images suffer a lower contrast that is homogeneous over the image, meaning the main portion of the APD value results from the lower contrast and not from worse structural resolution. The performance of the rearrangement results in APD would be even greater if the contrast of the images would have been adjusted manually.

**Fig. 8:**
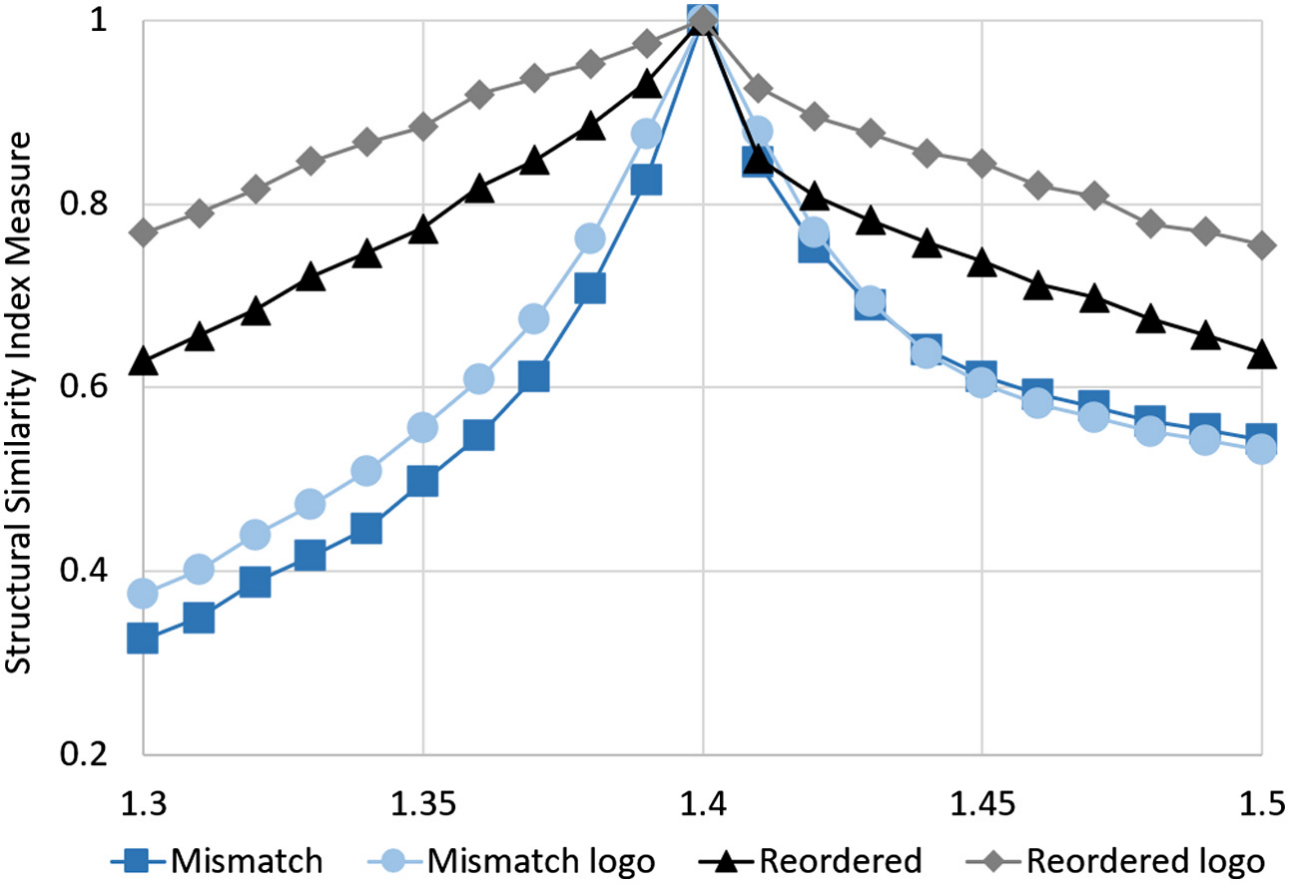
SSIM analysis of the same simulations as in Fig. 7. The greater the value is, the closer the evaluated image is to the reference image (perfect RI matched simulation).^17^

In contrast to APD, SSIM accounts more for structural similarity and less for total and regional differences in brightness. The effect of this difference in evaluation is visible in Fig. 8. In the case of a mismatch of 1.4 (medium) to 1.3 (sample), the reconstruction of the corrected sinogram is twice as good as the uncorrected result when only looking the logo region of the sample. The SSIM values of the uncorrected simulations drop below 0.5, which indicates very strong differences in appearance to the phantom image. Although the performance of the uncorrected simulations is very similar for the whole sample and logo region, the performance of the corrected simulations is much better in the logo region.

### 3.2 Validation Experiment

#### 3.2.1 Agarose cylinder

Exemplary SLOT measurements of fluorescent beads in an agarose cylinder were performed. Water was used as the immersion medium to illustrate an RI matched case. A silicone oil with an RI of 1.422 was used to showcase the issues of a poorly RI matched measurement and to test the capabilities of the correction algorithm in a real application.

**Fig. 9:**
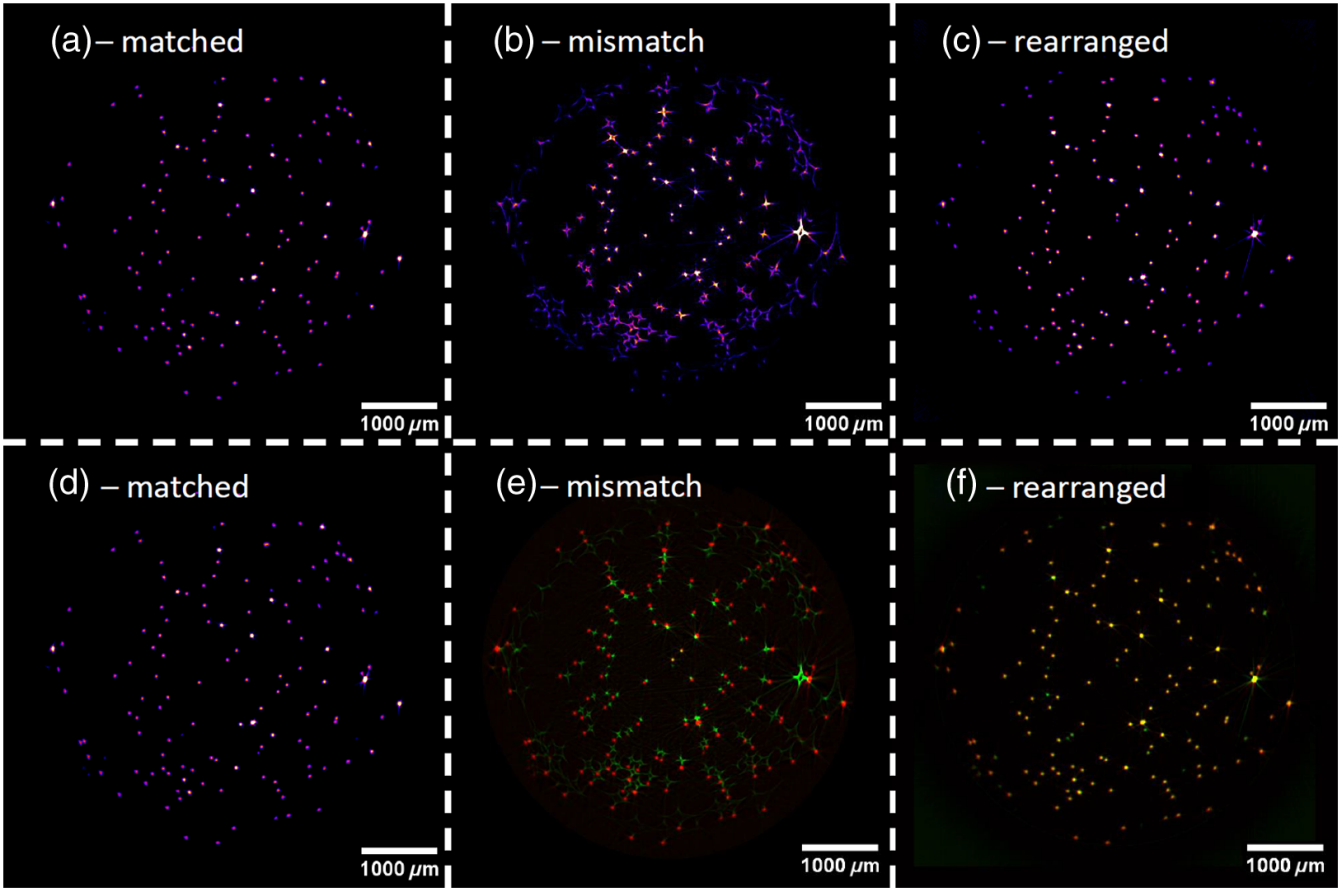
SLOT measurements of an agarose block with fluorescent polystyrene beads with different RI-matching configurations. All images show the average of 100 cross sections. The intensity of fluorescence was color-coded with the “fire” look-up table (ImageJ) for images (a)–(d). (a) Water was used as the immersion medium, which has a negligible RI difference compared to the agarose cylinder. (b) A silicone oil with an RI of 1.422 was used. (c) The measurement of (b) was corrected using the presented rearrangement method. (d) The same image as (a). Panels (e) and (f) have a different color-code: they are composites in which the results of the matched measurement are shown in red, and the mismatched measurement is shown in green. (e) Composite of the matched (red) and mismatched (green) measurement. (f) Composite of the matched (red) and the corrected (green) mismatched measurement.

Figure 9 shows the results of these measurements. The matched measurement shown in Fig. 9(a) displays spherical beads, which is the expected outcome. The mismatched measurement Fig. 9(b) shows strong artifacts, especially at the outer parts of the cylinder. The spherical shape of the beads is completely lost. Only the center part of the image is reasonably close to the matched case shown in Fig. 9(a). The measurement with the sinograms that were rearranged in post processing, shown in Fig. 9(c), is very close to the matched measurement. The fluorescent beads have a spherical shape, and the image quality is comparable to Fig. 9(a). The intensity of the fluorescence signal is falling off a bit at the outer parts of the image.

A different visualization of the same data is shown in the bottom row. The composites make it easier to compare the results. The effect of shrinking is much more visible in Fig. 9(e) than in Fig. 9(b). Figure 9(b) shows the composite of the matched Fig. 9(d) in red and rearranged Fig. 9(f) measurement in green. It is evident that the correction algorithm performs very well for the center part of the image, where almost every bead appears yellow, which means that the images are close to equal. The outer part is more reddish, which indicates a lower intensity in the corrected image. This is also visible in Fig. 9(c). A few completely red or green beads are visible. This is due to the cross sections not being perfectly set at the same heights for the matched and mismatched measurements.

**Fig. 10:**
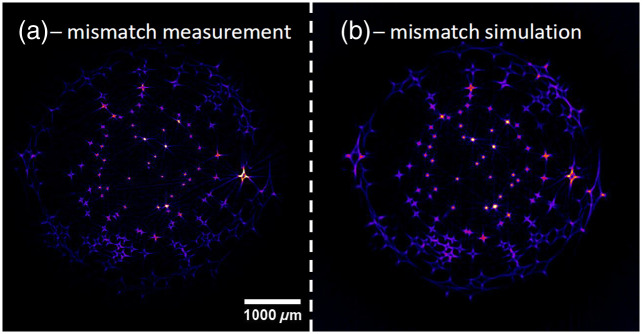
Comparison of measured (a) and simulated (b) SLOT images with an RI mismatch of 0.09. The rearranged, summed cross sections shown in Fig. 9(a) were used as the simulation phantom for (b). The simulation and the real measurement match well and show the same kind of image artifacts, which is the star-like smearing of the supposedly spherical beads.

The averaged cross sections shown in Fig. 9(a) were used as a phantom to cross-validate the results of the mismatched measurement. The result is shown in Fig. 10. The measurement and simulation match well. The same kind of artifacts appear in both images. The beads, which are not directly in the center of the sample, appear in a star-like shape. The drop-off in intensity toward the edge of the sample that was visible in Fig. 9(c) is also visible in this figure.

#### 3.2.2 Polydimethylsiloxan cylinder

**Fig. 11:**
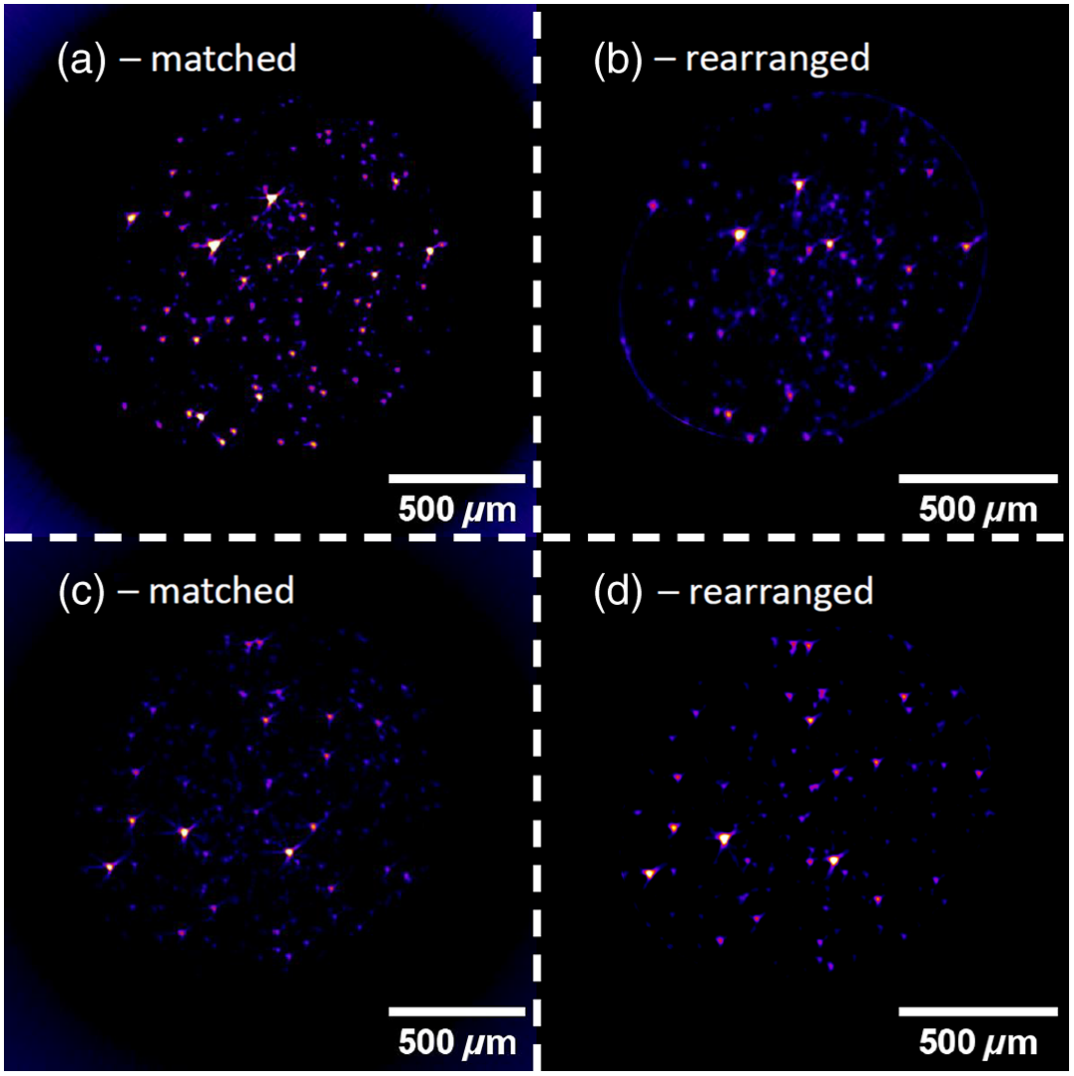
(a)–(d) RI matched and mismatched measurements of a PDMS waveguide with evenly distributed fluorescent nanoparticles. The images of each row show the same part of the sample. The images are averages of multiple image planes. The general appearance is similar for matched and mismatched measurements. The contrast is lower for the mismatched images.

A second validation experiment was performed on a PDMS cylinder with fluorescent nanoparticles. The purpose of this experiment was to show the inverse case of RI mismatch in which the RI of the sample is higher than the medium’s RI. The results of this experiment are shown in Fig. 11. Two different imaging planes of the same specimen are shown. The comparison of the matched and rearranged images show that the correction algorithm works fairly well for this case of RI mismatch. The contrast of the rearranged images is lower, and the nanoparticles appear less sharp. The drop-off in intensity when an RI mismatch is present, which was observable in the agarose cylinder measurements, is also visible in this case.

Not every fluorescent sphere appears in both measurements at the same position. This is because the specimen interacted with the silicone oil that was used as the immersion medium. The specimen grew in size, but the growth was not homogeneous. The geometry of the specimen changed, and the arrangement of the fluorescent spheres shifted unpredictably.

## 4 Discussion

It is clearly visible in Fig. 6 that a mismatch in RI is detrimental to the performance of SLOT if no correction is applied. The reconstructed outline of the sample is distorted, and the inner structure is smeared. The improvement of image quality is remarkable when the rearrangement method presented in this work is applied. The comparison of Fig. 6(3a) and 6(4a) highlights the effects. In the uncorrected images, only the general brightness can be extracted from the result, but in the corrected images almost all features of the source image are distinguishable.

An asymmetry regarding the RI mismatch is visible in the APD analysis (Fig. 7) when looking at values of the corrected images. A possible explanation for this is the strong refraction of light beams that hit the sample at the edges when an RI mismatch is present. Two different phenomena are present, depending on the type of RI mismatch.

In cases in which the RI of the sample is smaller than the RI of the medium, the light gets refracted away from the center of the sample. This effect reduces the information density for the outer parts of a projection because neighboring rays fan out. This can be viewed as a reduced sampling rate compared to parallel light rays. This also leads to the shrinking observed in Figs. 6(1a) and 6(1b).

In extreme cases, the total internal reflection can prevent the light ray from entering the sample all together. This leads to a dark spot in the sinogram and lower reconstruction quality, even when digital correction is applied.

In cases in which the RI of the sample is higher than the RI of the medium, the light gets refracted toward the middle of the sample. This results in a magnification effect that makes the inner parts of the reconstructed image look enlarged when no correction is applied to the sinogram. For steep incident angles and large RI mismatches, this leads to a complete loss of information, which cannot be corrected by the method presented in this paper. The presented method depends on the availability of data for each incident point and rotation angle. But if the rays at the edges of a sample get refracted too strongly toward the middle of the sample, no data for the outer incident points are recorded. This is visible in Fig. 6(2e), where no information is available at the edge of the sample. This effect is also seen in the SSIM-analysis in Fig. 8, where the logo region, which is located in the center of the sample, has a much higher SSIM than the whole sample region for every increment of RI mismatch.

The validation experiment with the agarose cylinder shows very promising results. First, it validates the simulations of mismatched RI SLOT measurements. This is clearly visible in Fig. 10, where a measurement with an RI mismatch and a simulation with the same RI mismatch are compared. Aside from the drop-off in intensity toward the edge of the sample, the measurement and simulation are very similar. The effect of shrinking, which was also observed in the simulations in Fig. 6, is very prominent in Figs. 9(b) and 9(e). Shrinking happens when the RI of the medium is higher than the RI of the sample.

The star-like artifacts of the mismatched measurement were completely corrected, and the resulting image is very close to the measurement with a matched RI. The drop-off in intensity toward the edge of the sample is an issue. This is not caused by the correction algorithm. This conclusion is drawn from the fact that the mismatched measurement shown in Fig. 10 also has a lower intensity toward the edge of the sample, and the simulated mismatched image does not. There is also no significant intensity drop-off visible in the simulated and corrected images shown in Fig. 6 row 2, which supports this conclusion. The lower intensity at the outer parts of the real measurement likely results from the following two issues.

A part of the excitation beam gets reflected at the interface of the medium and the sample when an RI mismatch is present. This reflected part increases with the rate of RI mismatch and when the incident angle becomes more acute. This relation is described in detail in the Fresnel equations.^9^

The more prominent reason for the loss in intensity would be the poor surface quality of the agarose cylinder. This leads to light scattering when an RI mismatch is present. This effect has a stronger influence at more acute incident angles because the light effectively hits a larger surface area, so more light can be scattered and multiple scattering can happen, too. This scattering is much weaker when the RI is perfectly matched.

The second validation experiment confirms the validity of the correction algorithm for cases in which the RI of the sample is higher than the RI of the medium. This case is very interesting because it enables the use of water as the immersion medium. Using water instead of silicon oil has many advantages. Aside from the facts that water is super available and cheap, it is also much easier to handle. Silicone oil is very persistent, and cleaning the glass cuvette that holds the immersion medium is very time consuming and tedious. Using silicone oil can, in some cases, destroy or heavily influence the specimen, as was the case with the PDMS cylinder. Water did not interact with the specimen at all.

A point-wise acquisition such as in SLOT is typically slower than a snapshot acquisition in optical projection tomography or light-sheet microscopy, but it allows for the correction of images through ray propagation. The precise correction of an acquisition with a significant RI mismatch is not possible in a snapshot acquisition without knowledge of the phase of the wavefront such as in optical diffraction tomography. Algorithms with a comparable correction to the one presented in this work have been developed for optical coherence tomography. There, a deep learning approach was realized to correct the distorted position of an insertion needle for robotic eye surgery.^27^

## 5 Conclusion

In this work, an approach to correct SLOT data that were acquired with a mismatch in RI between the sample and medium was presented. Simulations of uncorrected and rearranged measurements were compared. Evidence was presented that the correction works well and greatly improves the perceived image quality compared to uncorrected images. Although the best result was still achieved when no RI mismatch was present, this approach will allow for more freedom when choosing immersion media for SLOT. It could also reduce the need of precisely measuring the RIs of the sample and medium because these values can be adjusted in the rearrangement of the sinogram to achieve the highest image quality. Validation experiments were conducted with an RI mismatch of about 0.09 in both mismatch cases. The results are very promising as the image quality of the corrected measurement is very comparable to the RI matched measurement.

## Supplementary Material



## Data Availability

The code of the SLOT simulation and correction software and Supplementary Materials are available at https://github.com/LZHBO/SLOT-Mismatch-Correction.
