# Supplementary material for: Imperfect refractive index matching in scanning laser optical tomography and a method for digital correction
Source: J Biomed Opt. 2024 May 15;29(6):066004. doi: 10.1117/1.JBO.29.6.066004 (PMC11095122; doi:10.1117/1.JBO.29.6.066004)
Supplement: Supplementary file 1 [file JBO_029_066004_SD001.pdf]

Individual images of the simulated SLOT measurements (figure 6,7,8) which are also available at https://github.com/LZHBO/SLOT-Mismatch-Correction/tree/main/data/data%20set%20lzh%20logo :

RI mismatch, ordered from medium RI of 1.3 to 1.5

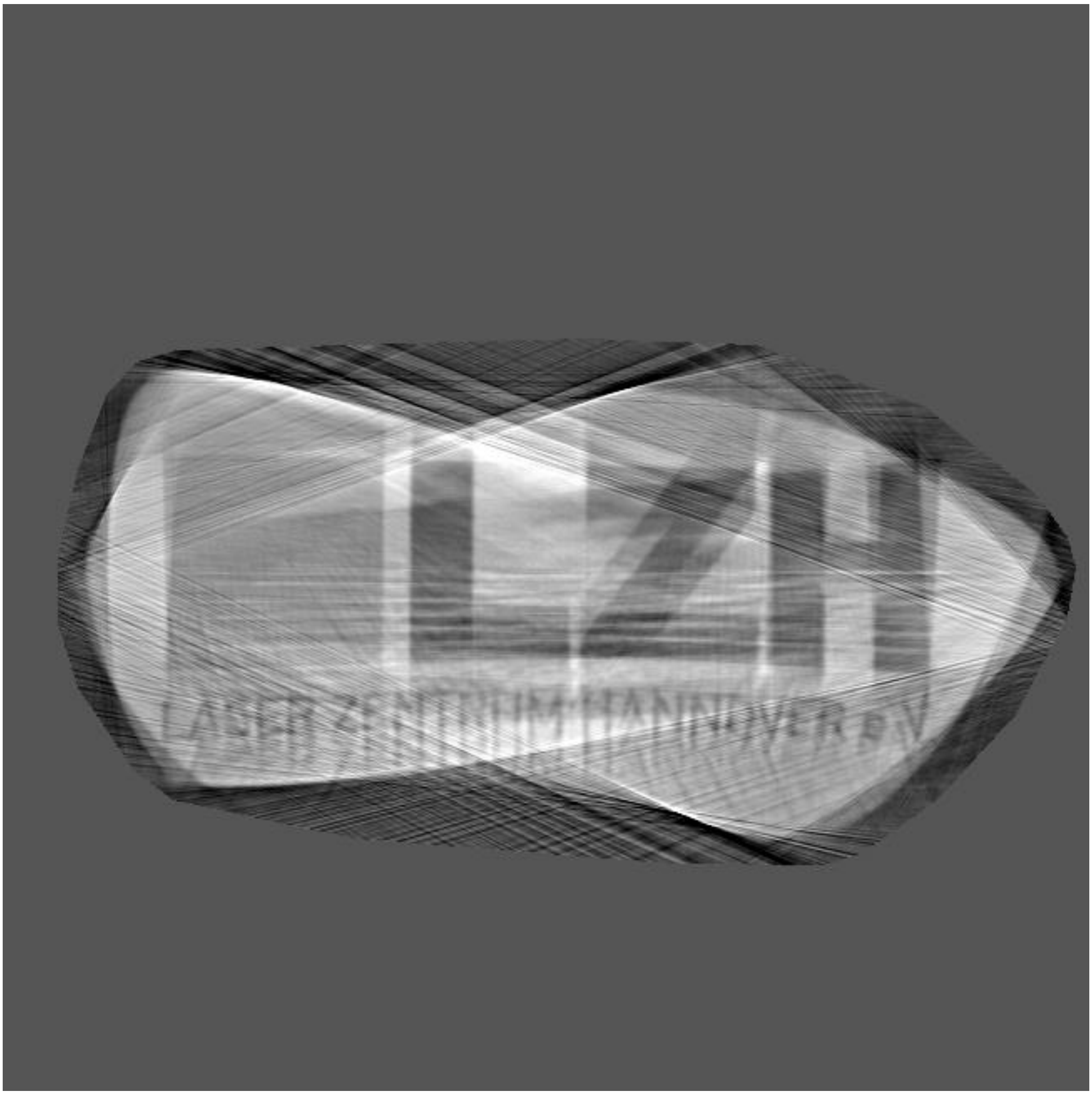

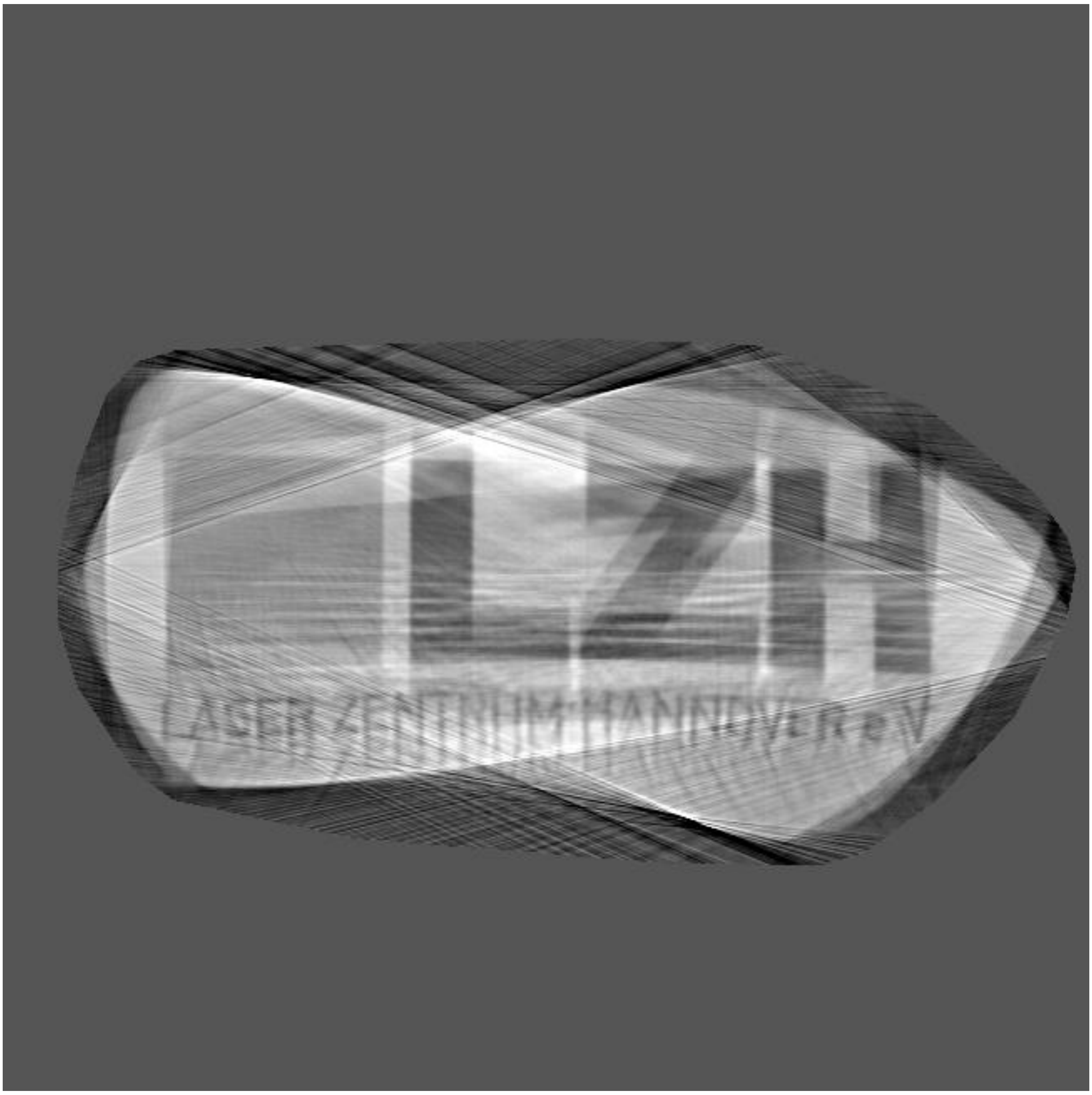
LZH
LASER ZENTRUM HANNOVER e.V

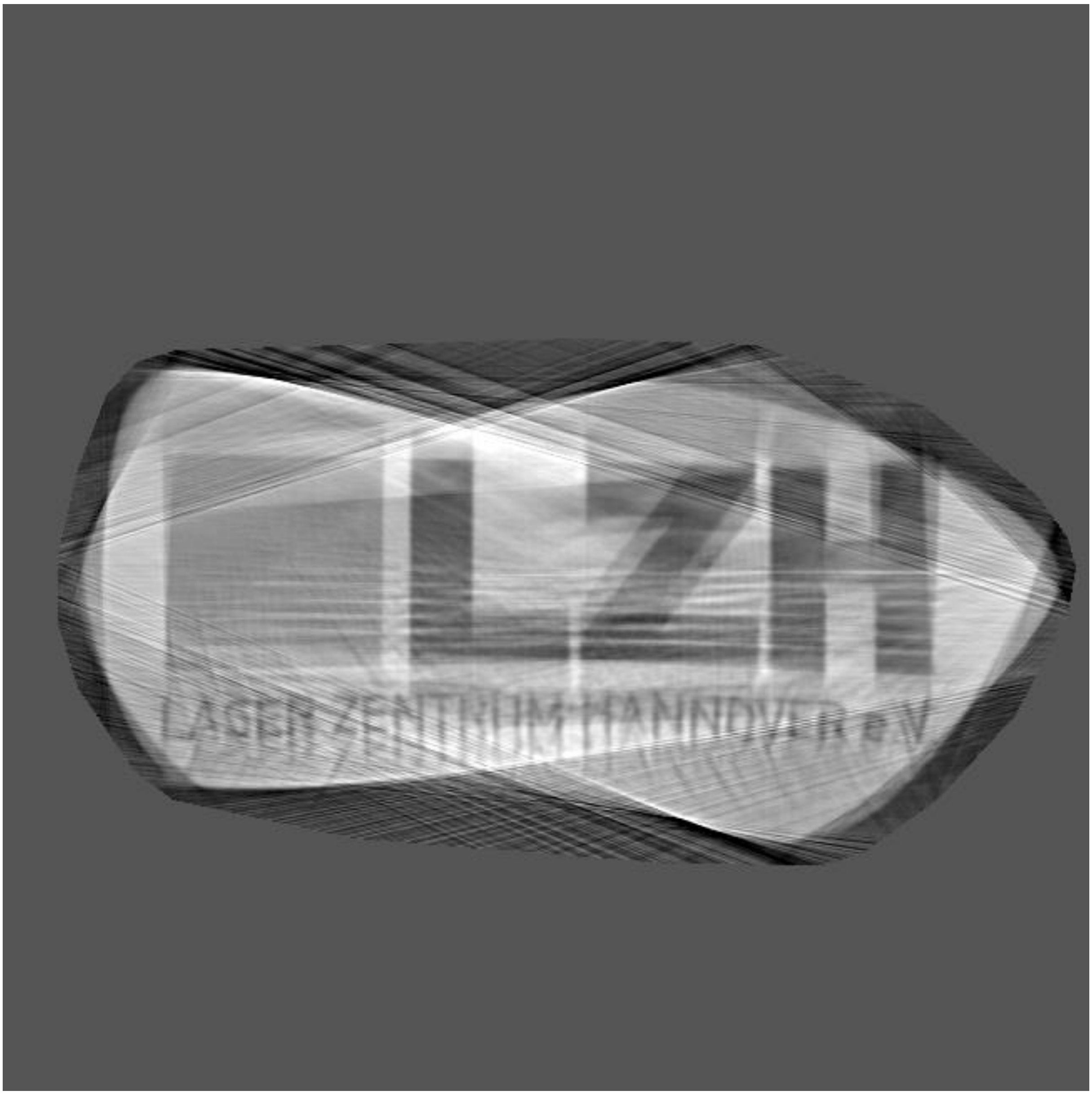
LZH

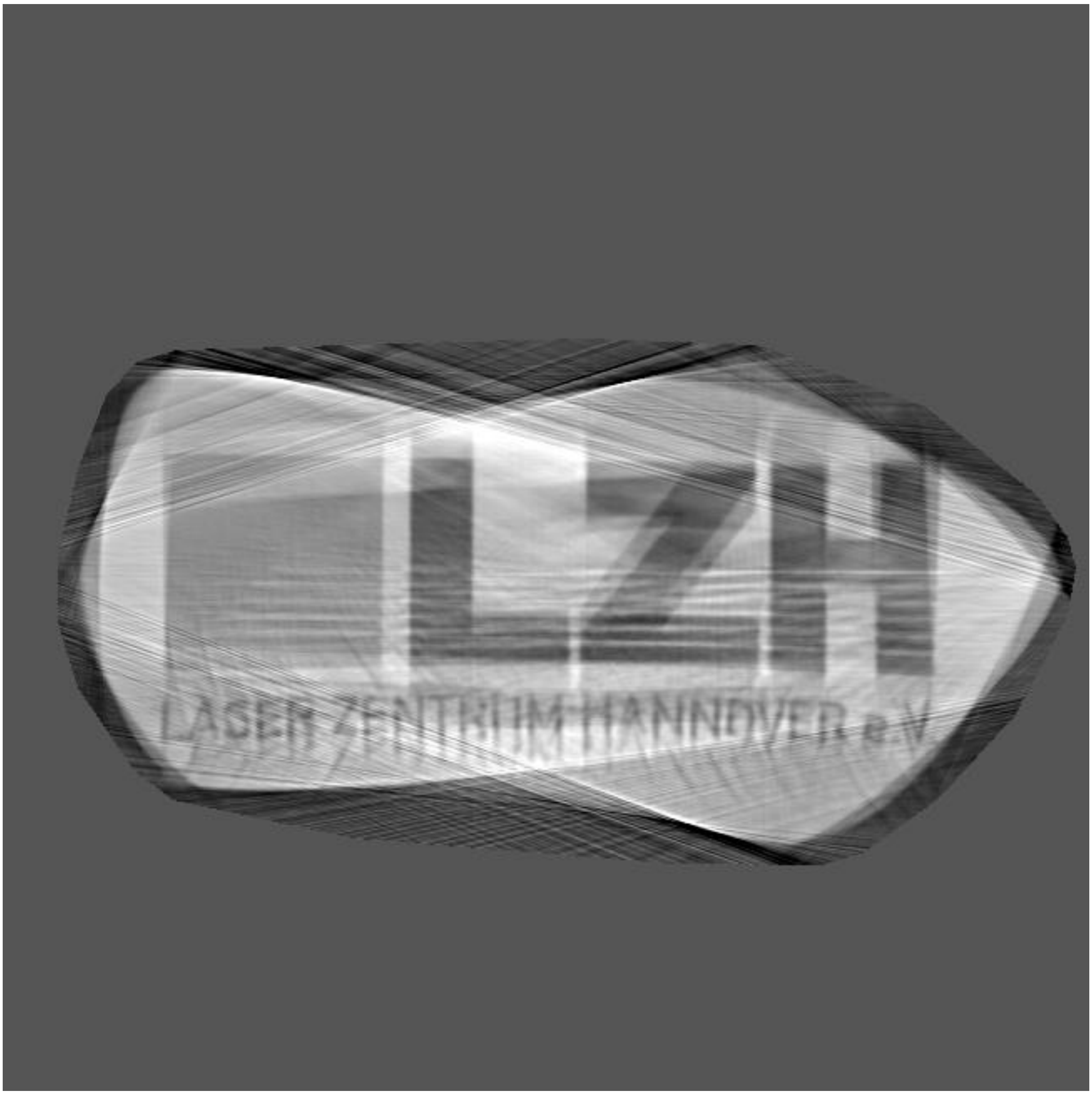
LZH
LASER ZENTRUM HANNOVER e.V.

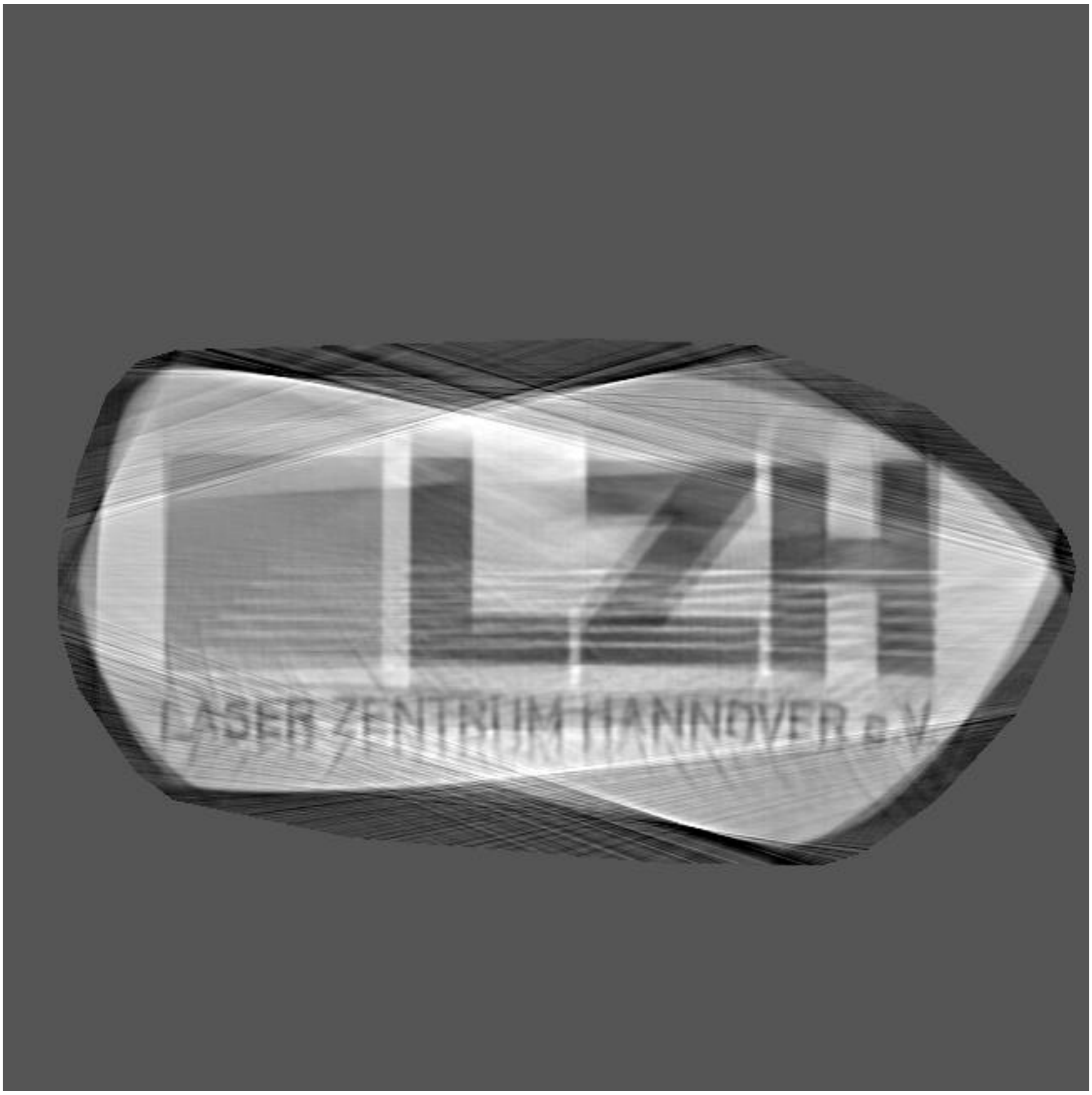
LZH
LASER ZENTRUM HANNOVER e.V.

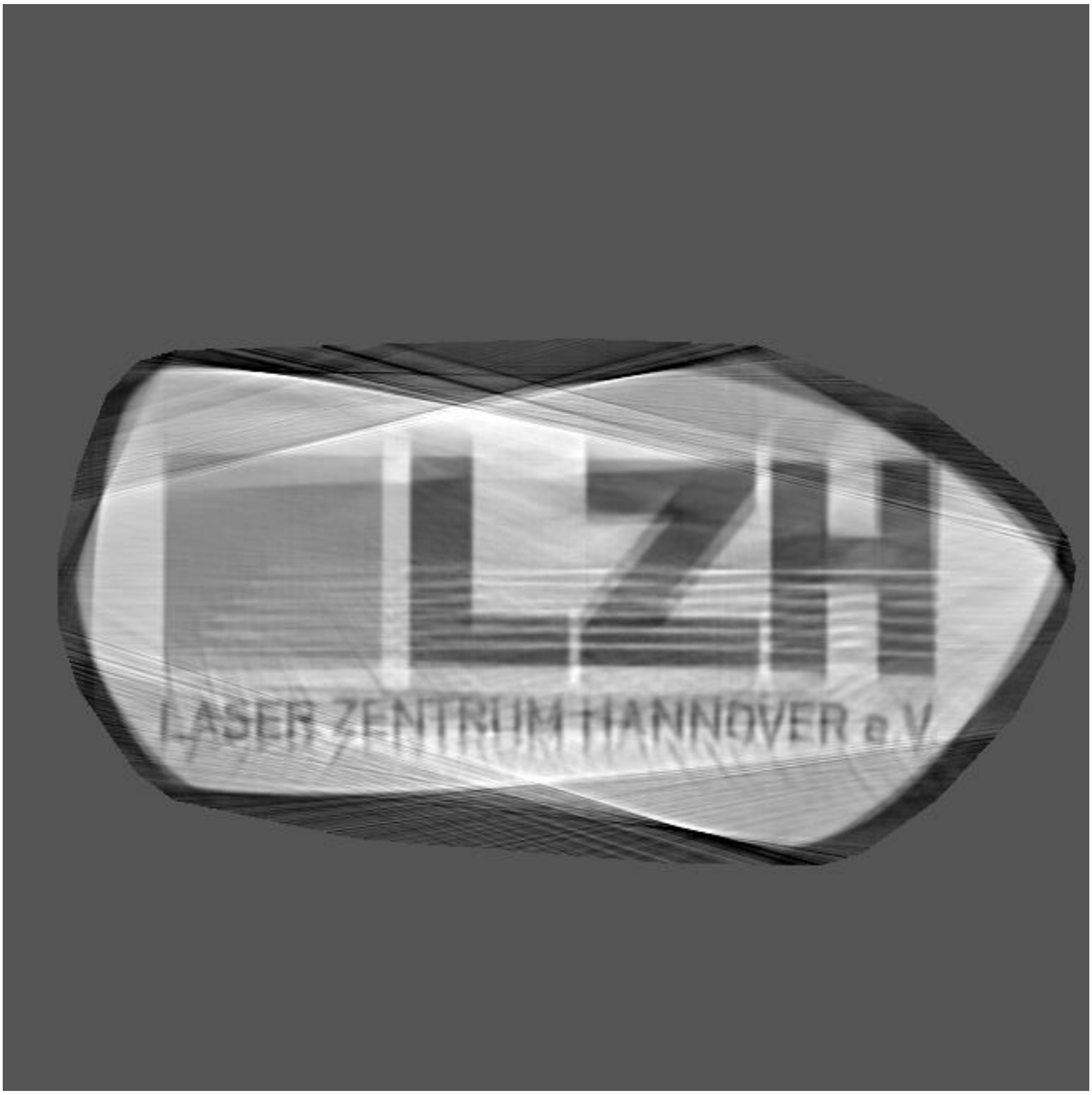
LZH
LASER ZENTRUM HANNOVER e.V.

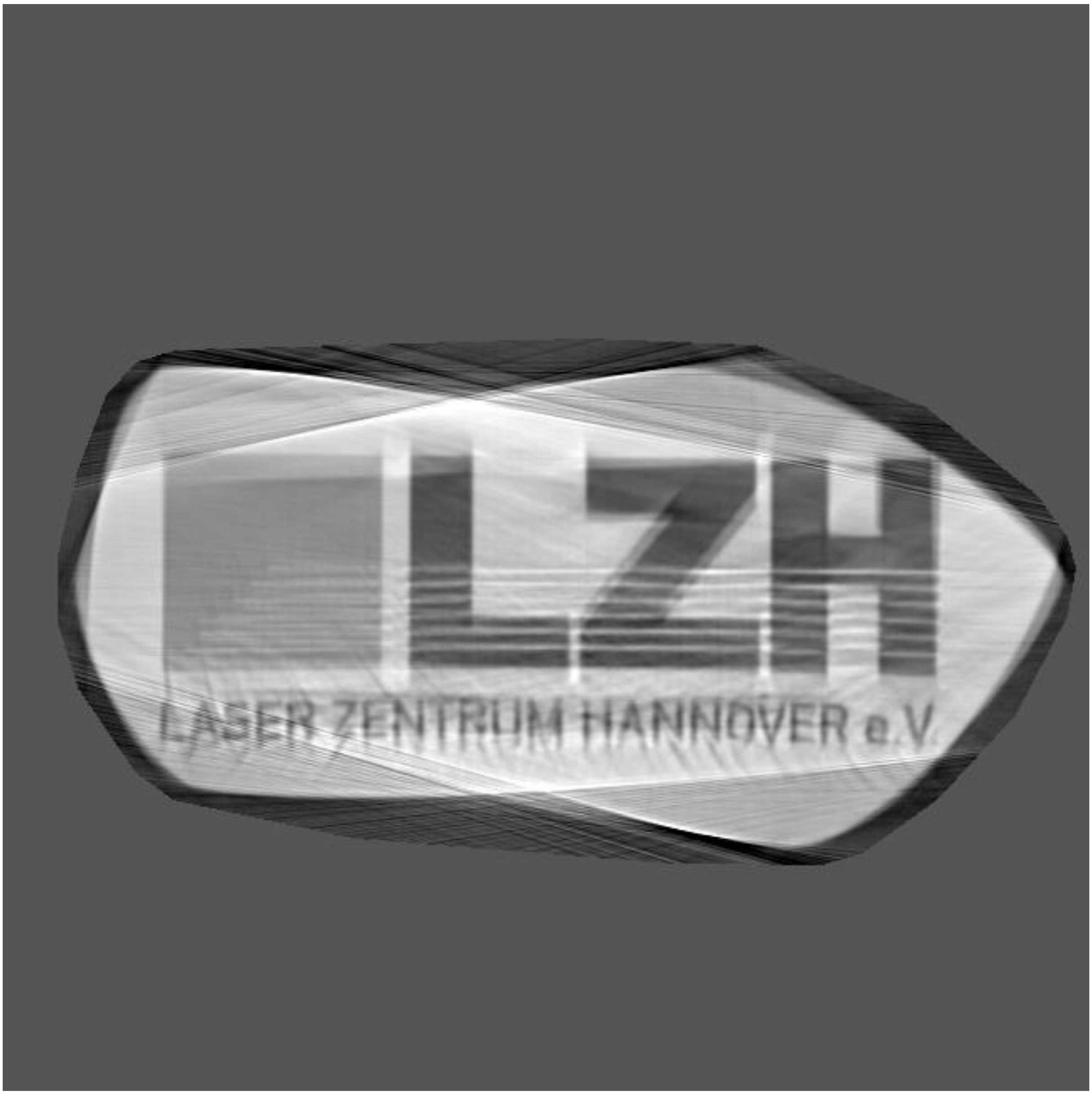
LZH
LASER ZENTRUM HANNOVER e.V.

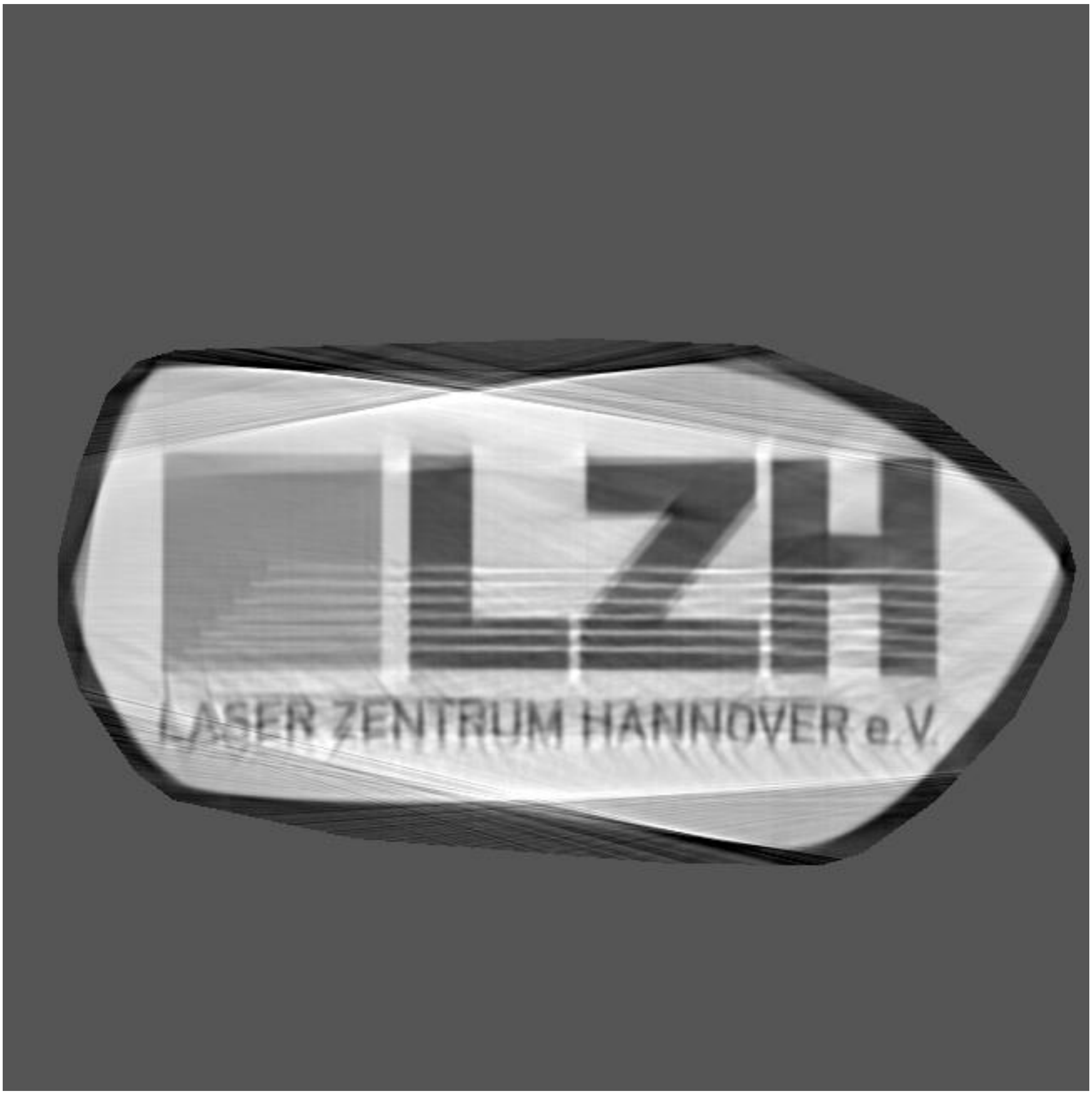
LZH
LASER ZENTRUM HANNOVER e.V.

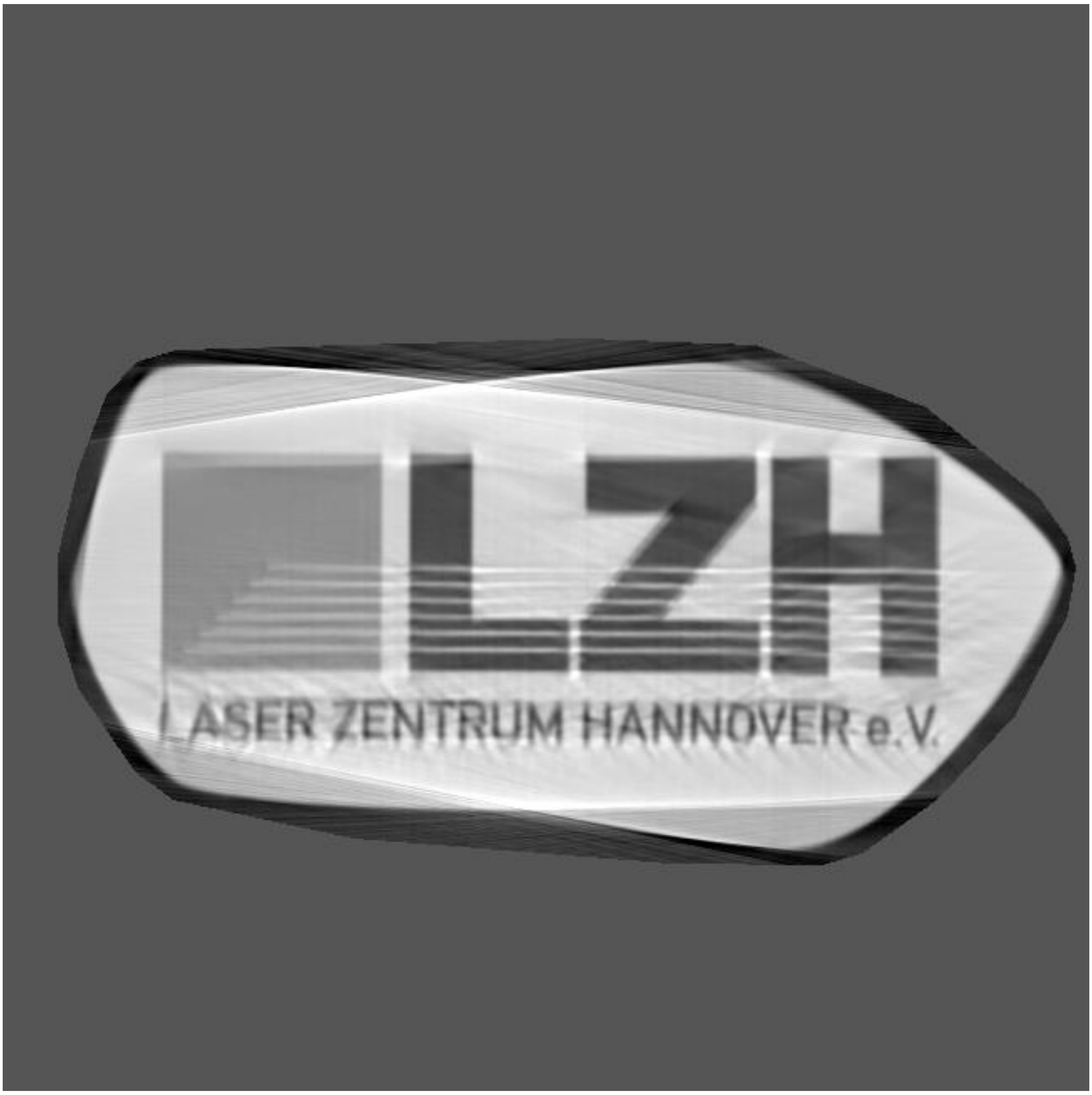
LZH
LASER ZENTRUM HANNOVER e.V.

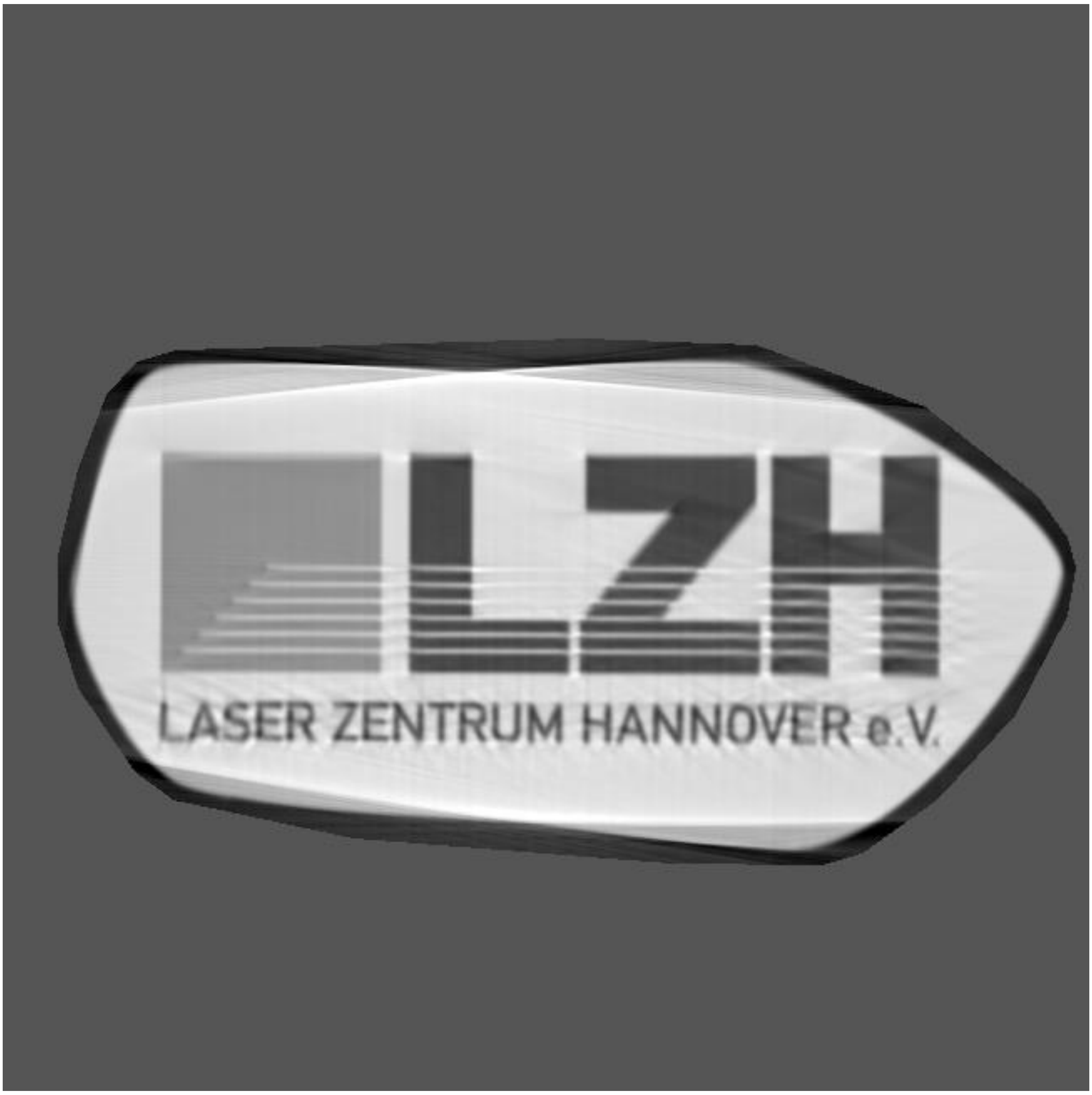
LZH
LASER ZENTRUM HANNOVER e.V.

LZH

LASER ZENTRUM HANNOVER e.V.

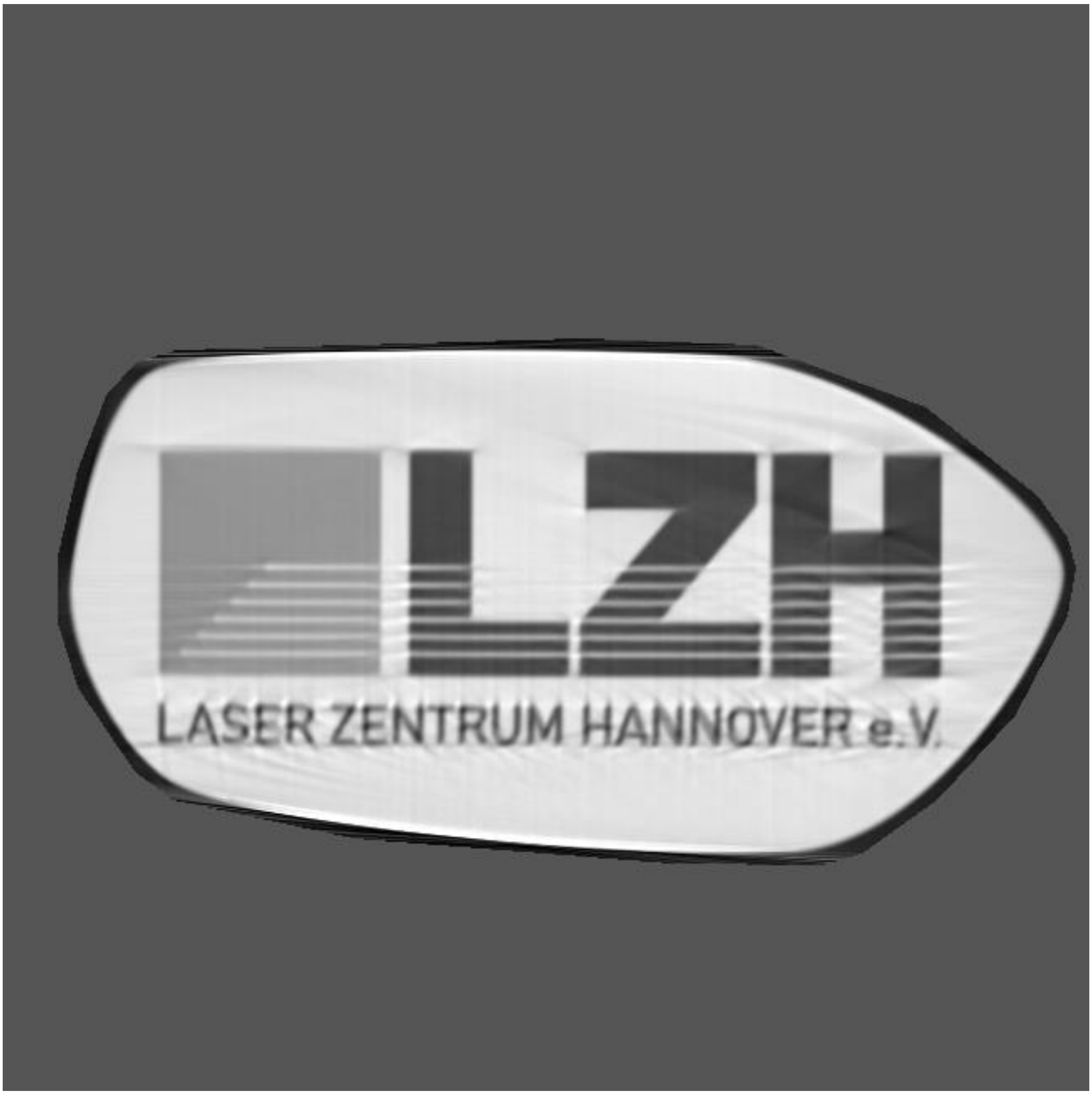
LZH
LASER ZENTRUM HANNOVER e.V.

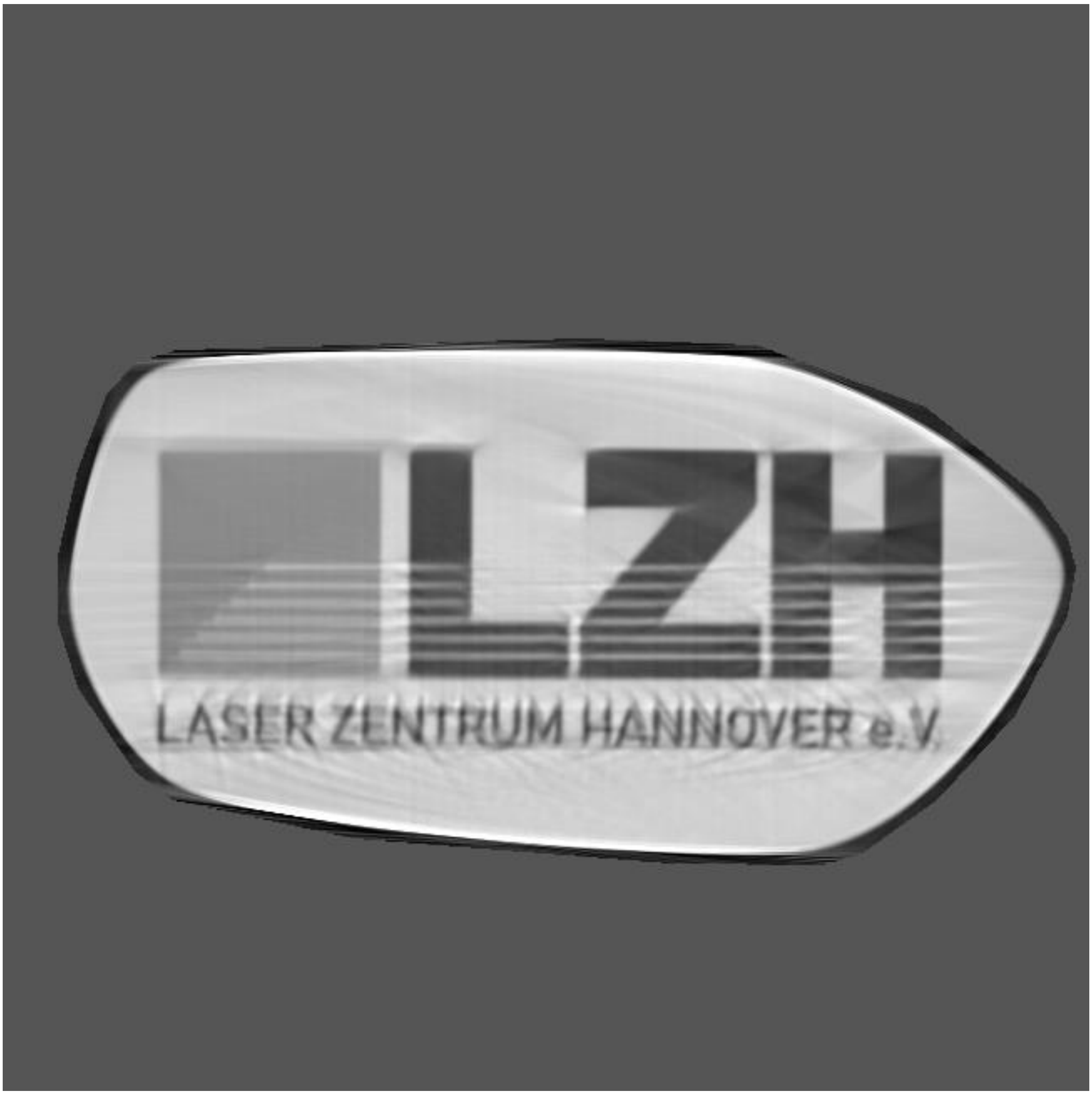
LZH
LASER ZENTRUM HANNOVER e.V.

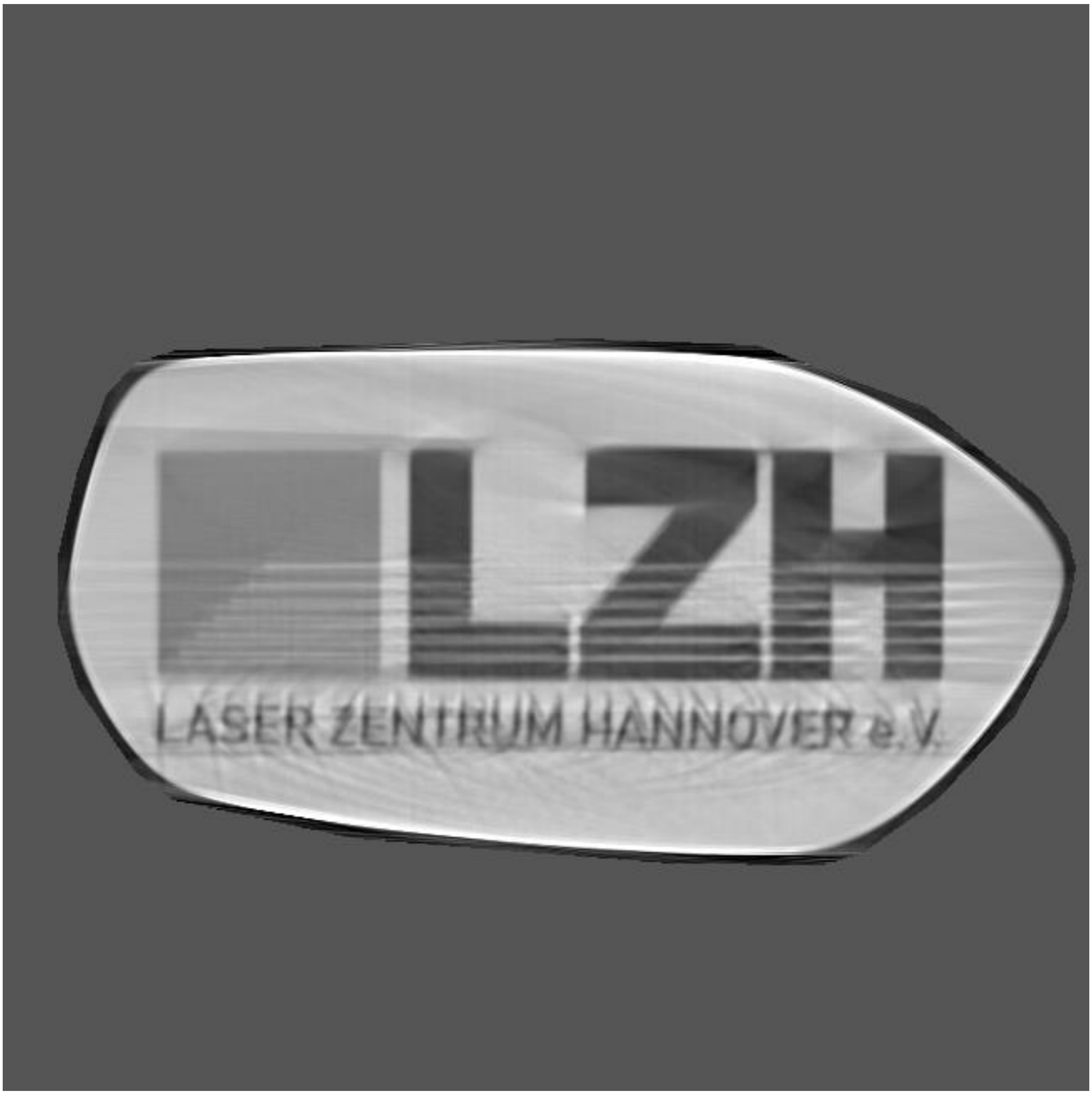
LZH
LASER ZENTRUM HANNOVER e.V.

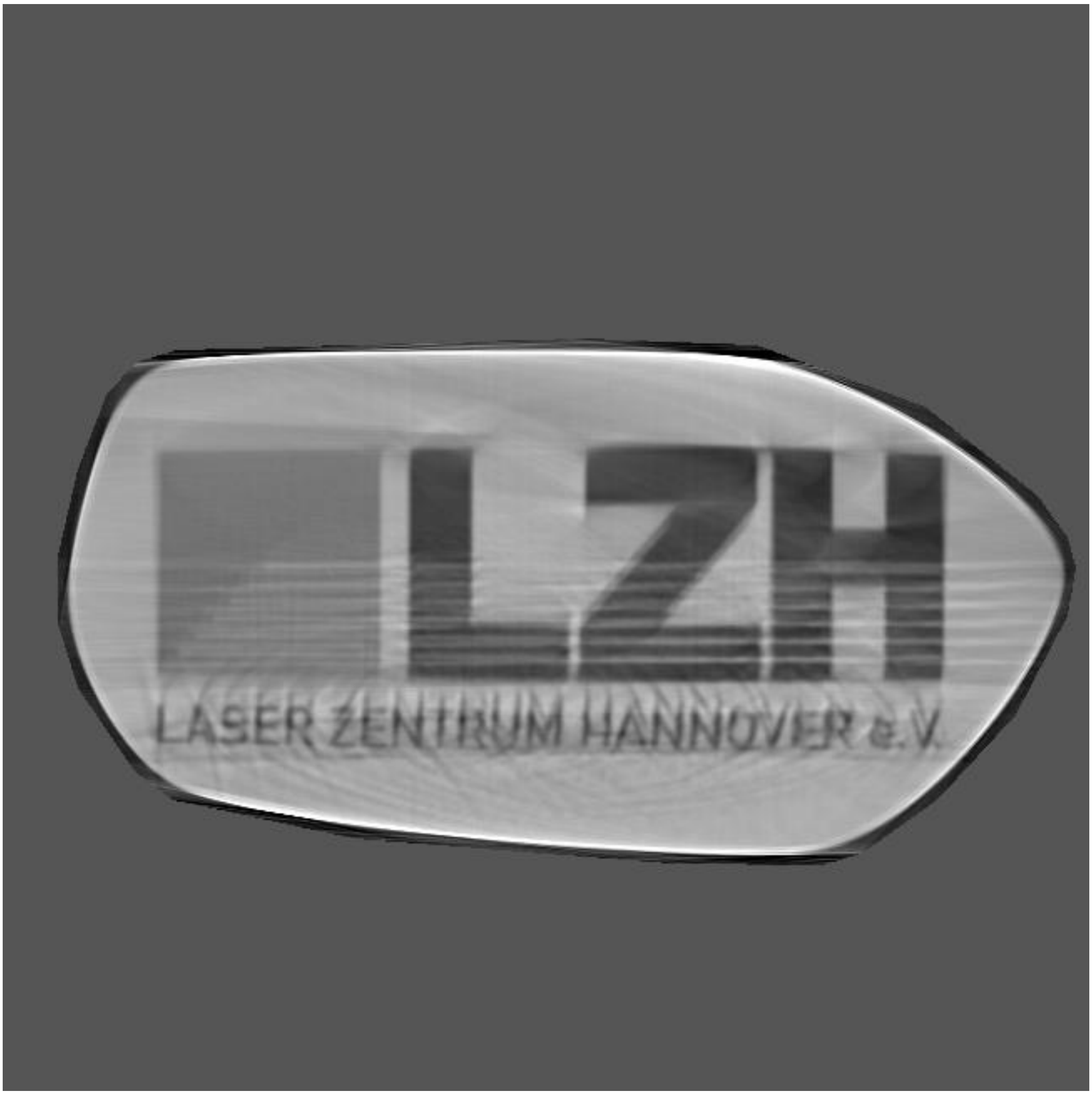
LZH
LASER ZENTRUM HANNOVER e.V.

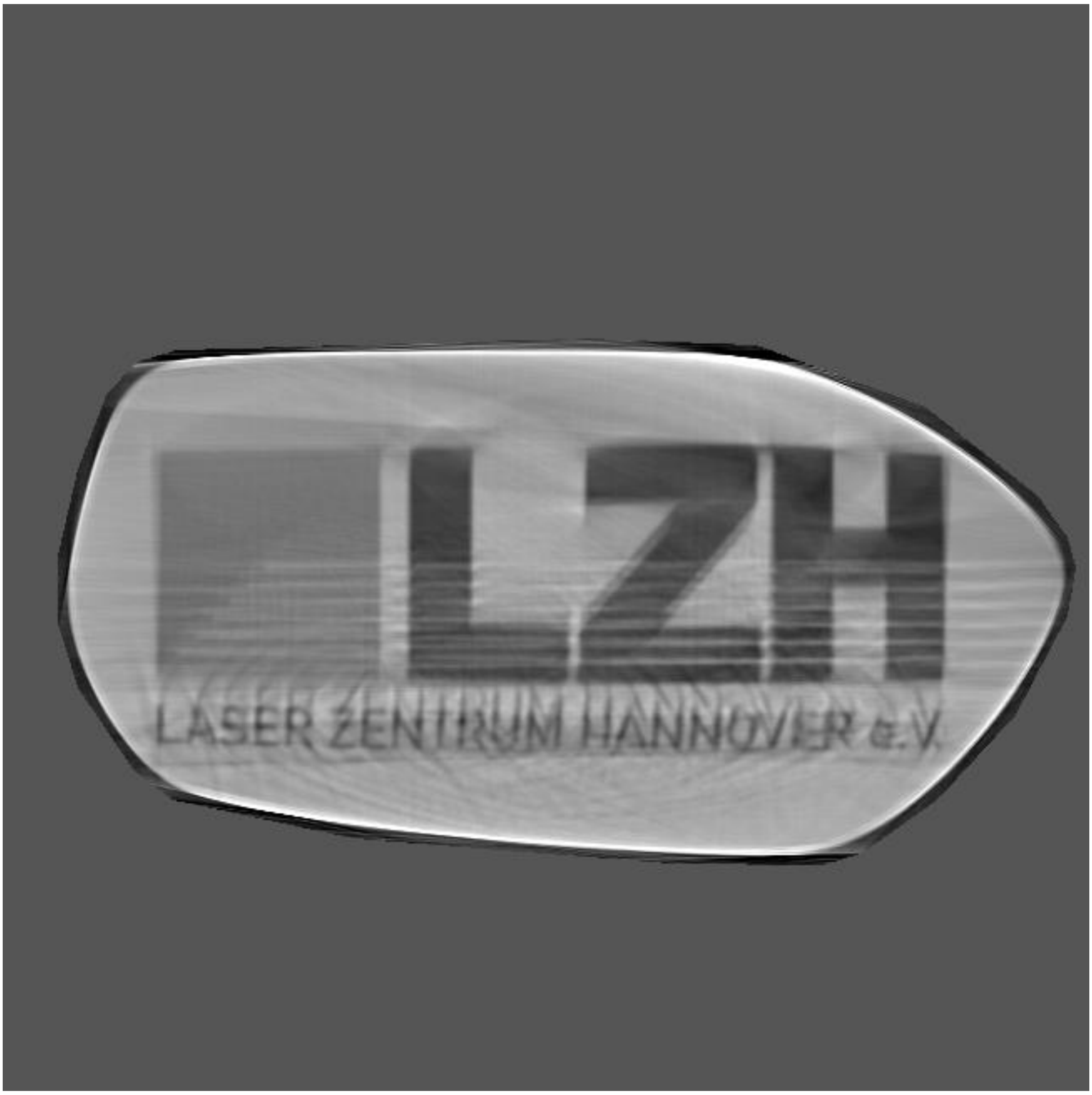
LZH
LASER ZENTRUM HANNOVER e.V.

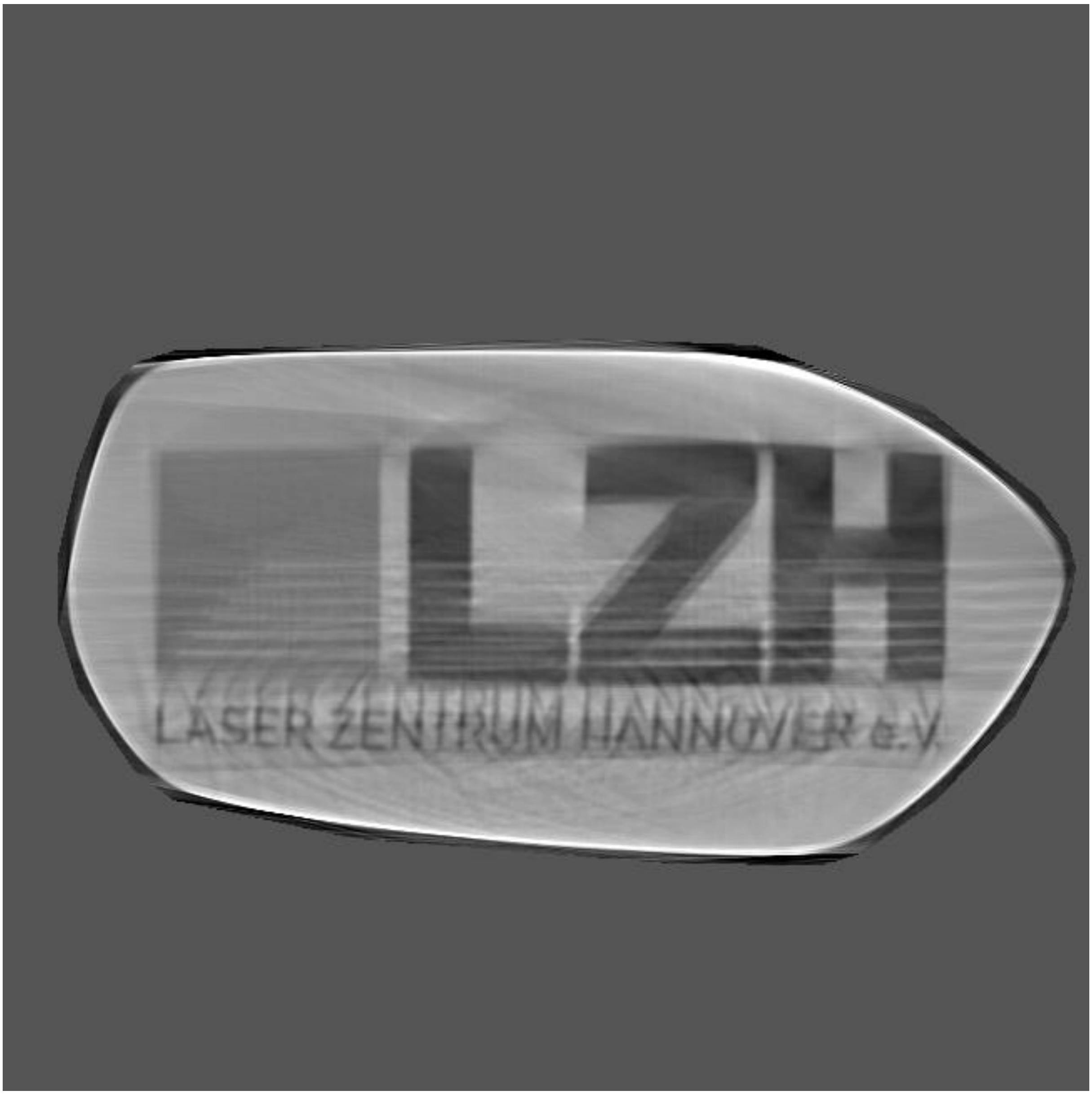
LZH
LASER ZENTRUM HANNOVER e.V.

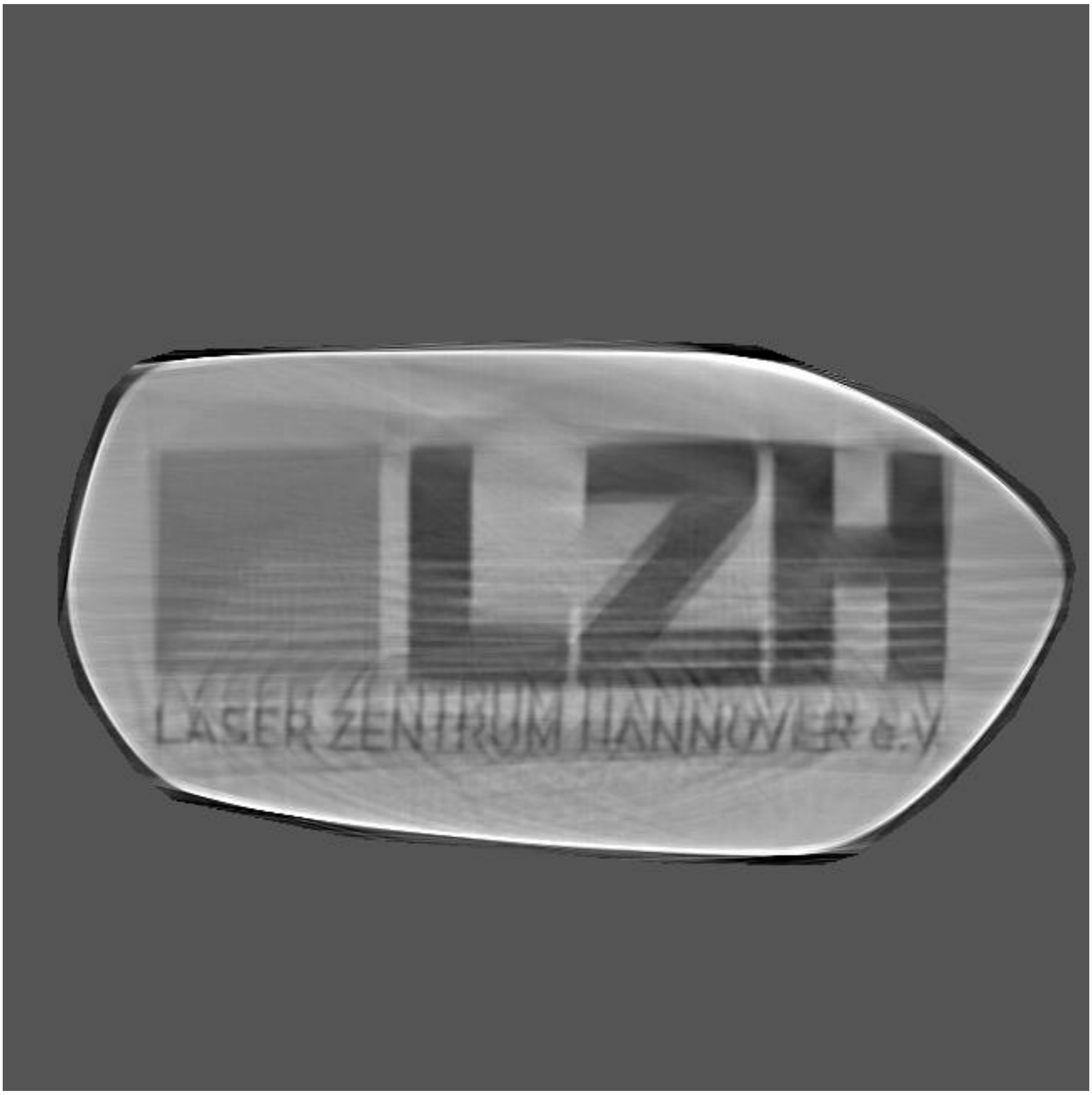
LZH
LASER ZENTRUM HANNOVER e.V

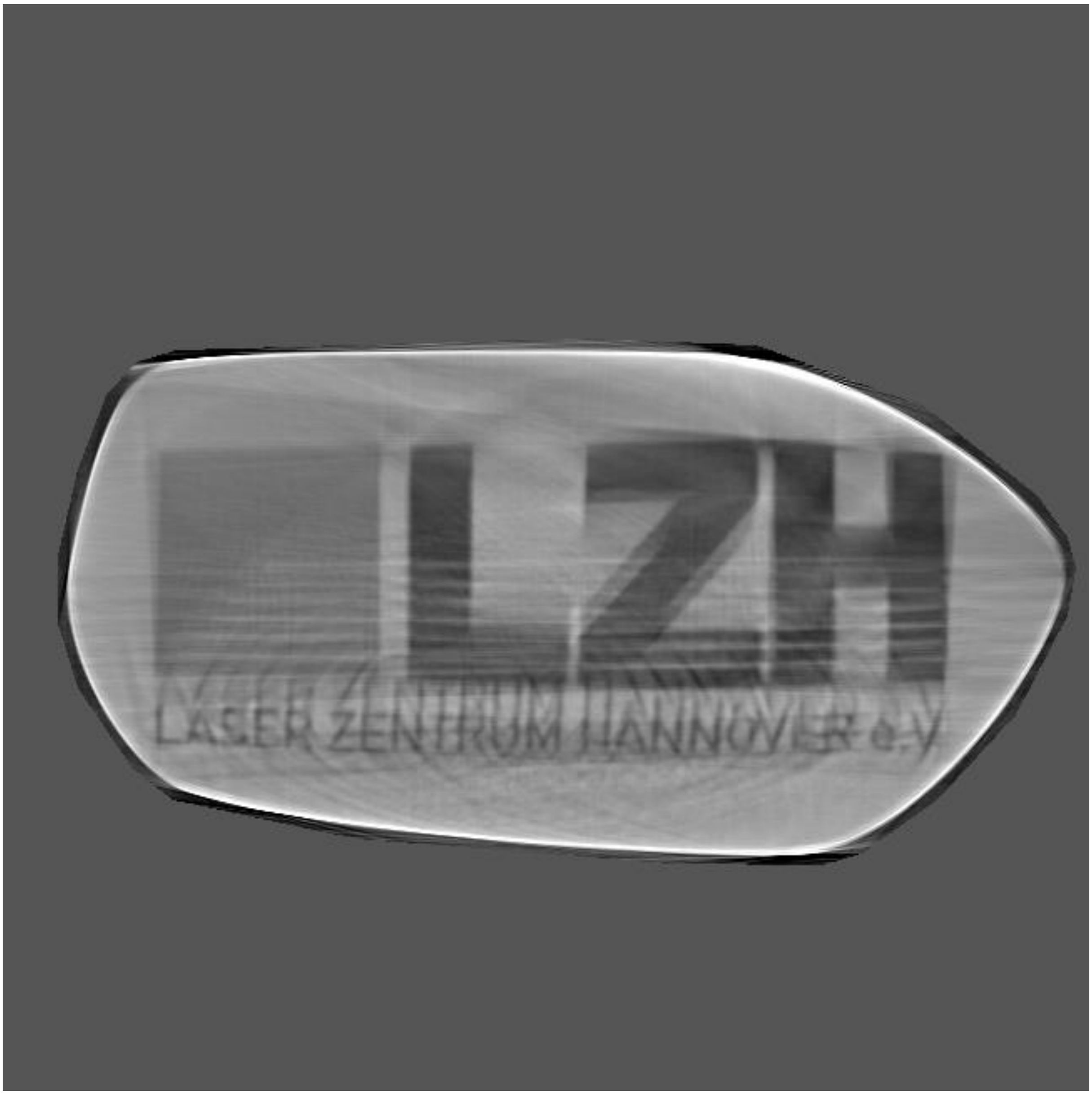
LZH
LASER ZENTRUM HANNOVER e.V

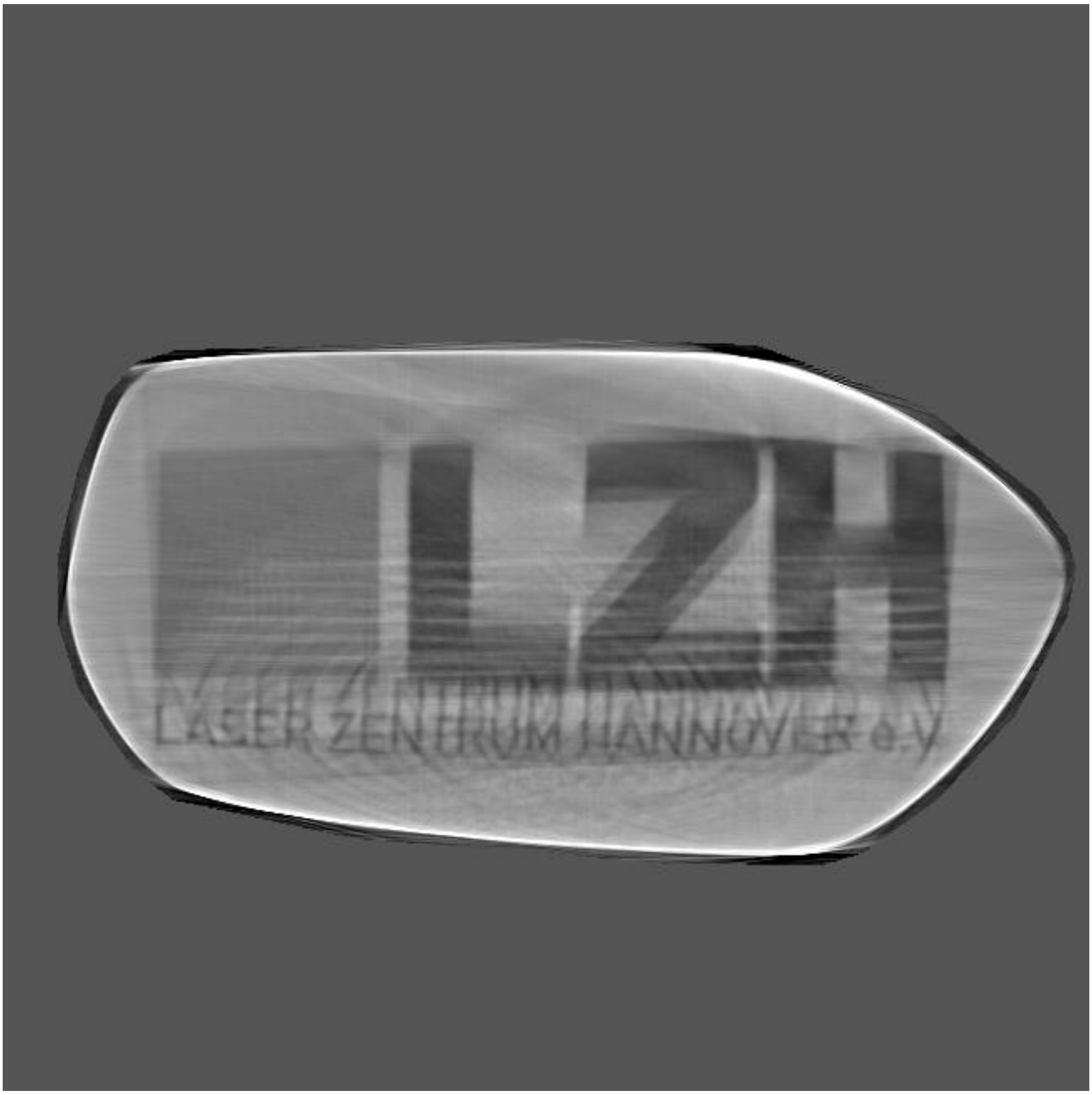
LZH
LASER ZENTRUM HANNOVER e.V

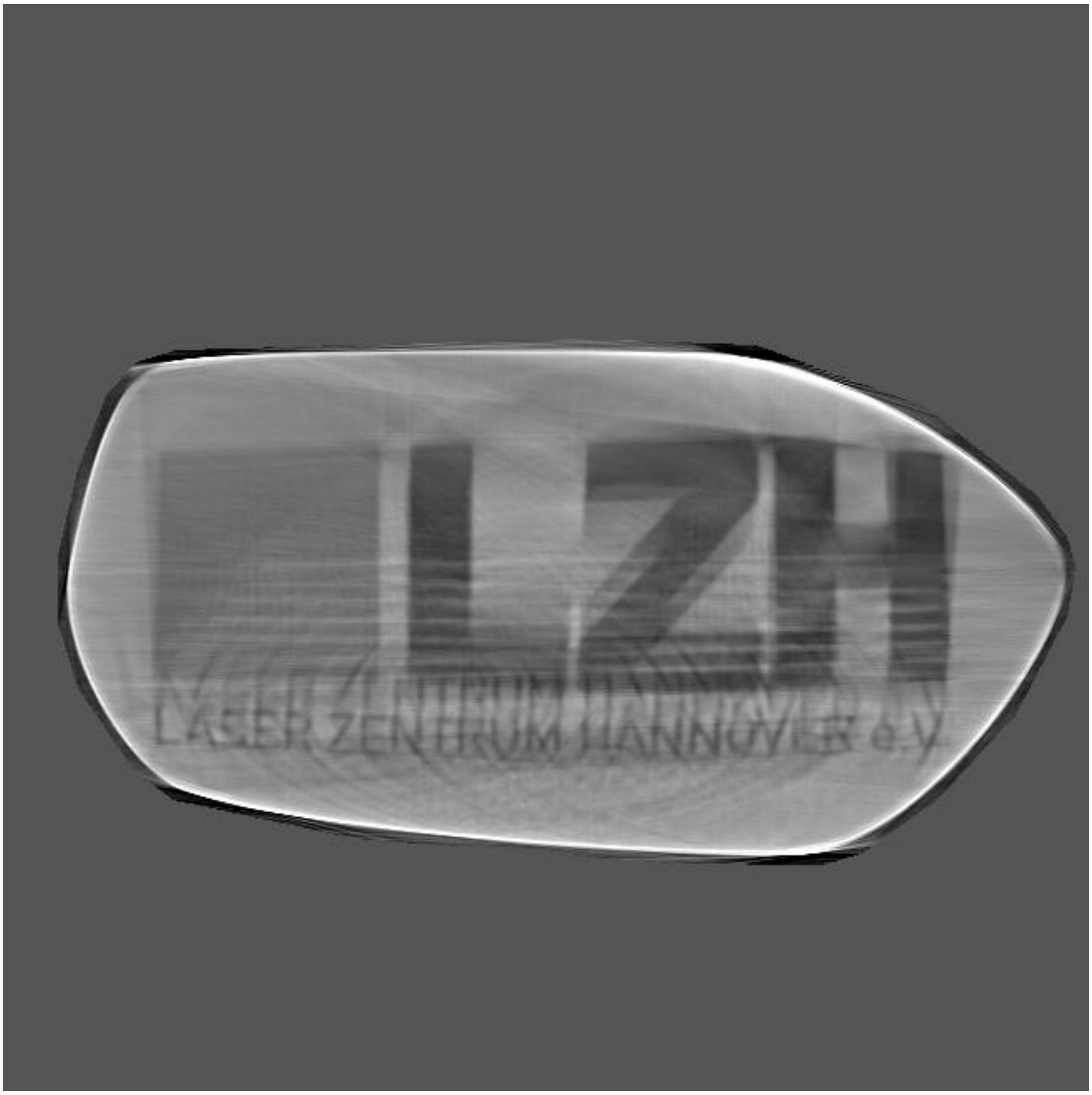
LZH
LASER ZENTRUM HANNOVER e.V.

Corresponding, simulated SLOT measurements where the correction method was applied, also figure 6,7,8 and ordered with a medium RI of 1.3 to 1.5

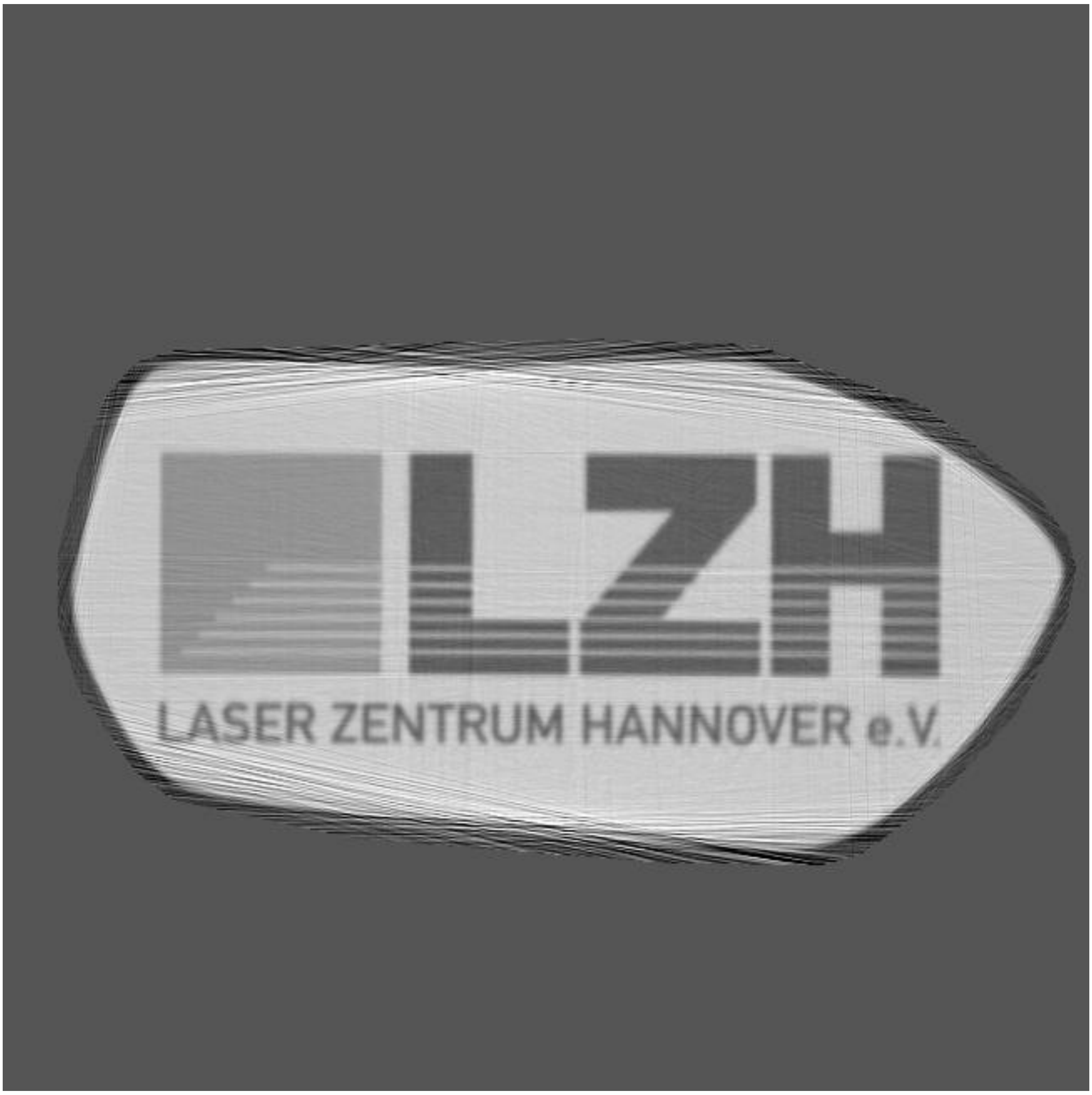

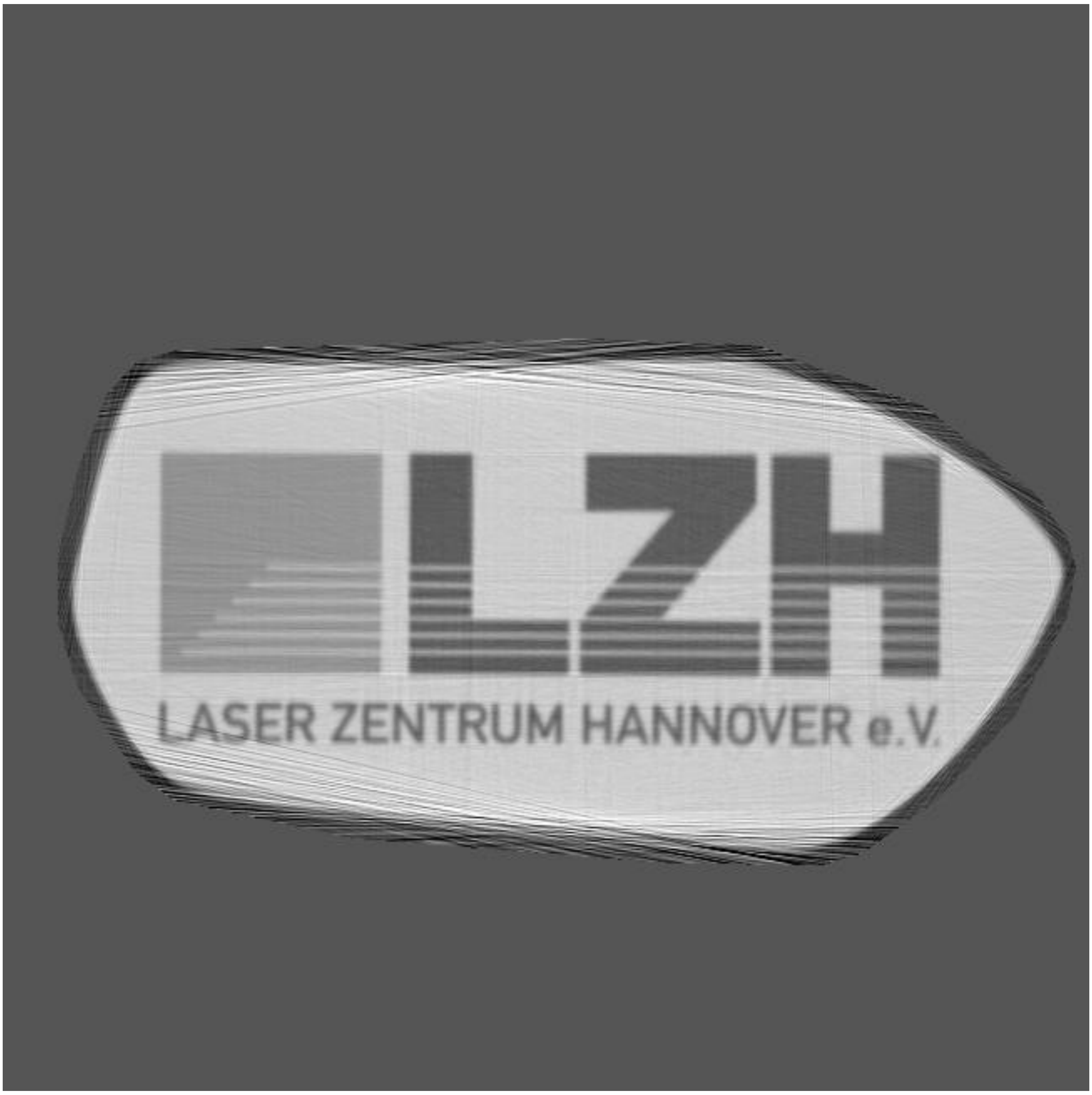
LZH
LASER ZENTRUM HANNOVER e.V.

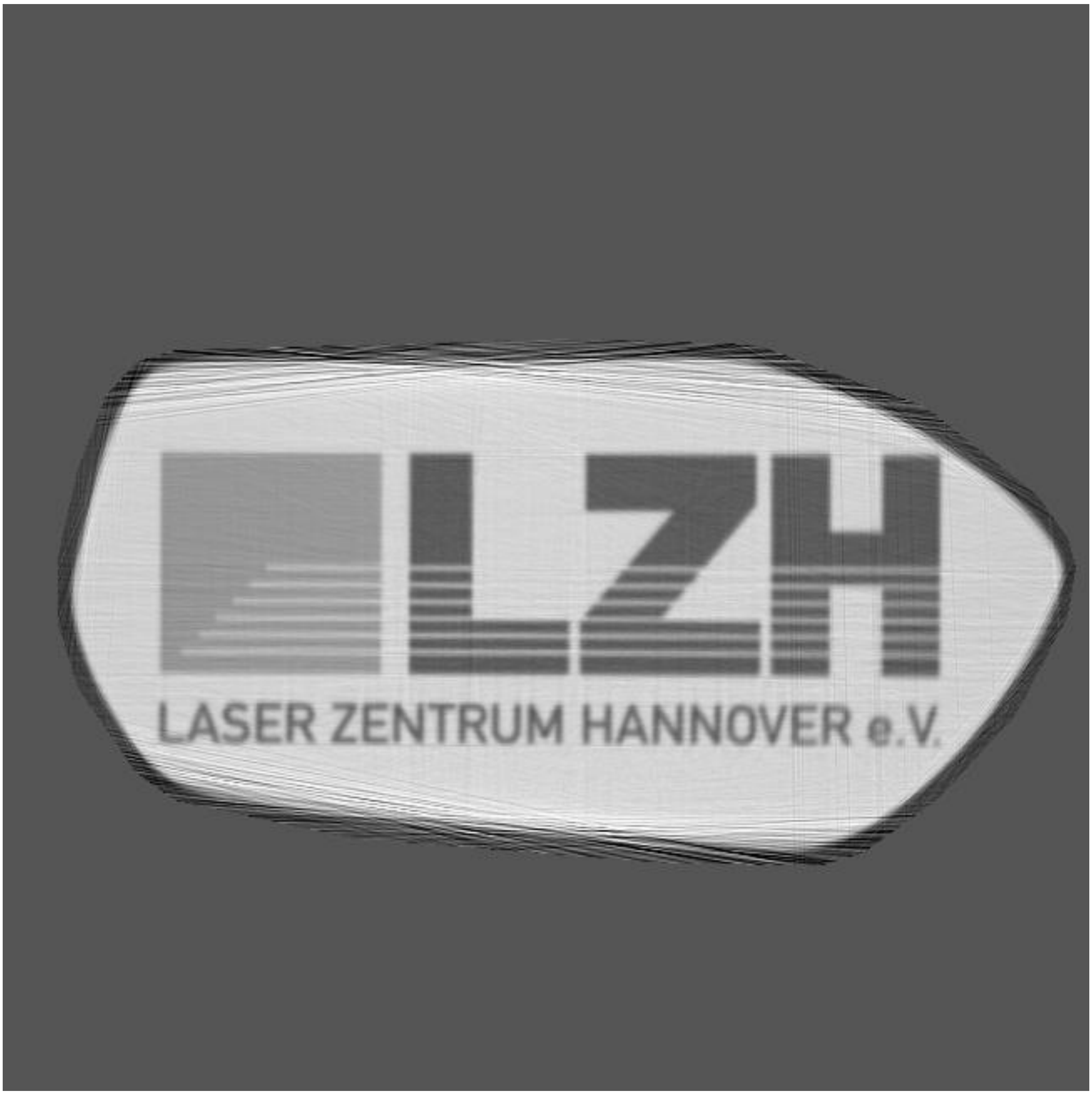
LZH
LASER ZENTRUM HANNOVER e.V.

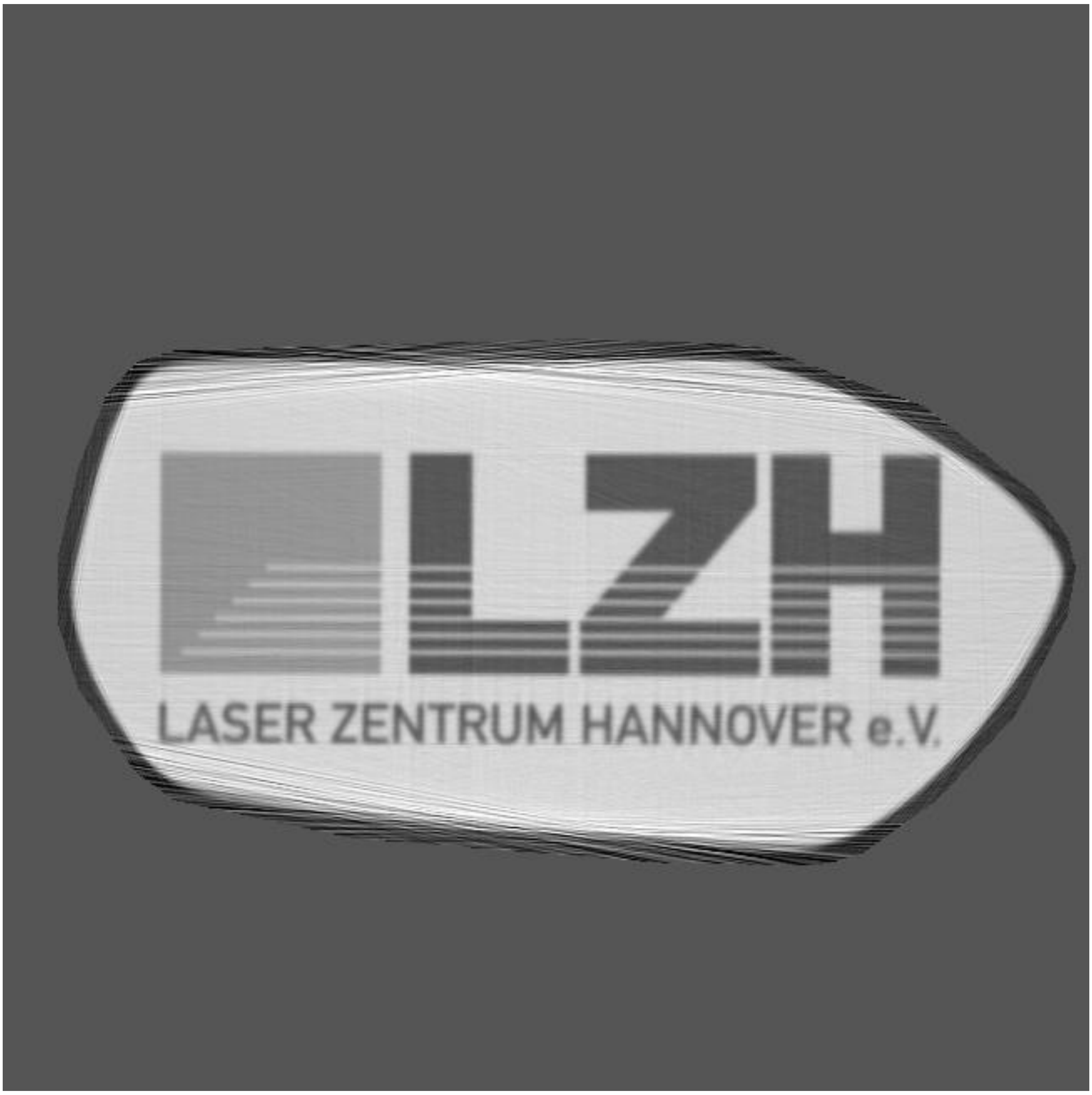
LZH
LASER ZENTRUM HANNOVER e.V.

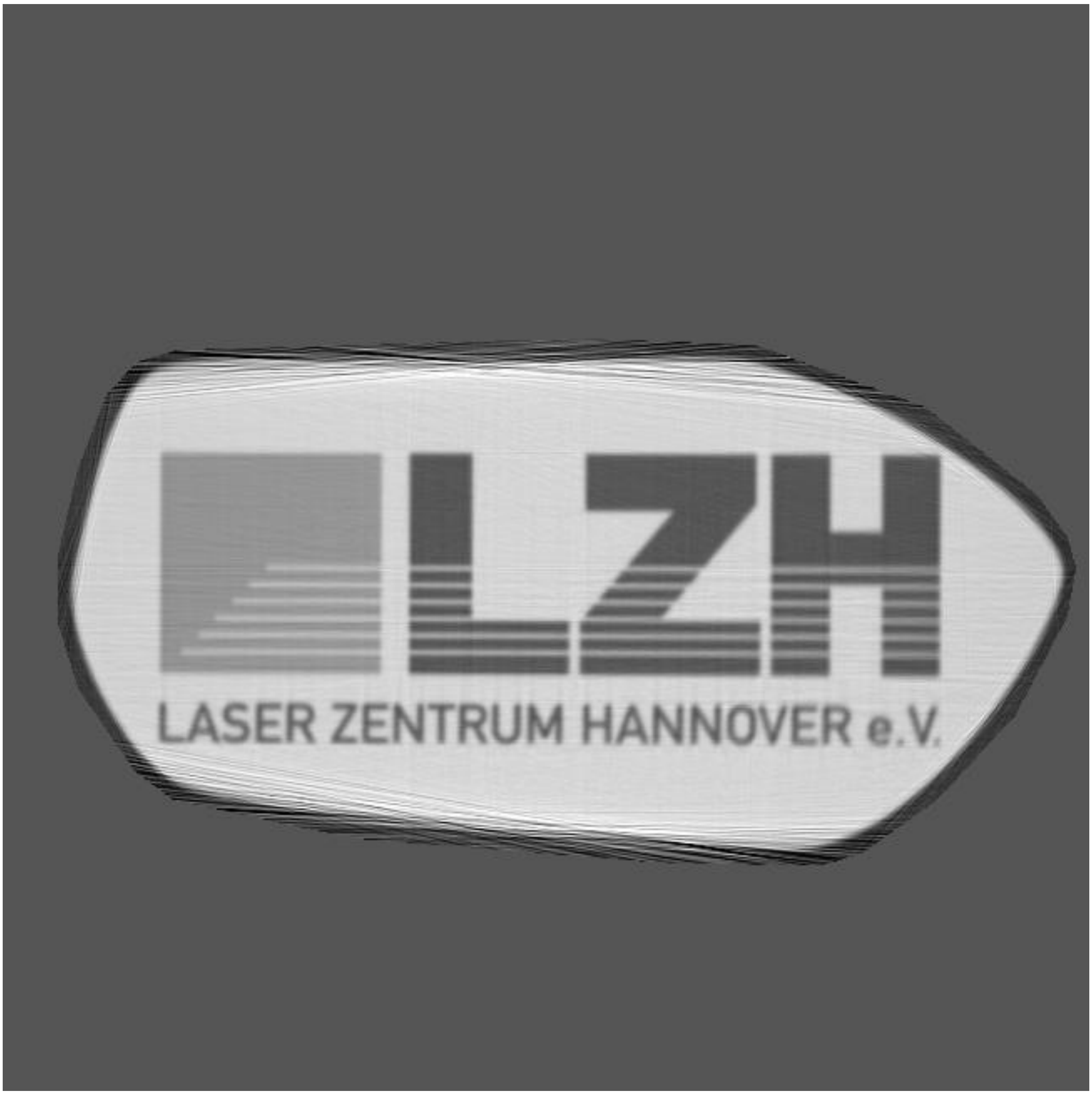
LZH
LASER ZENTRUM HANNOVER e.V.

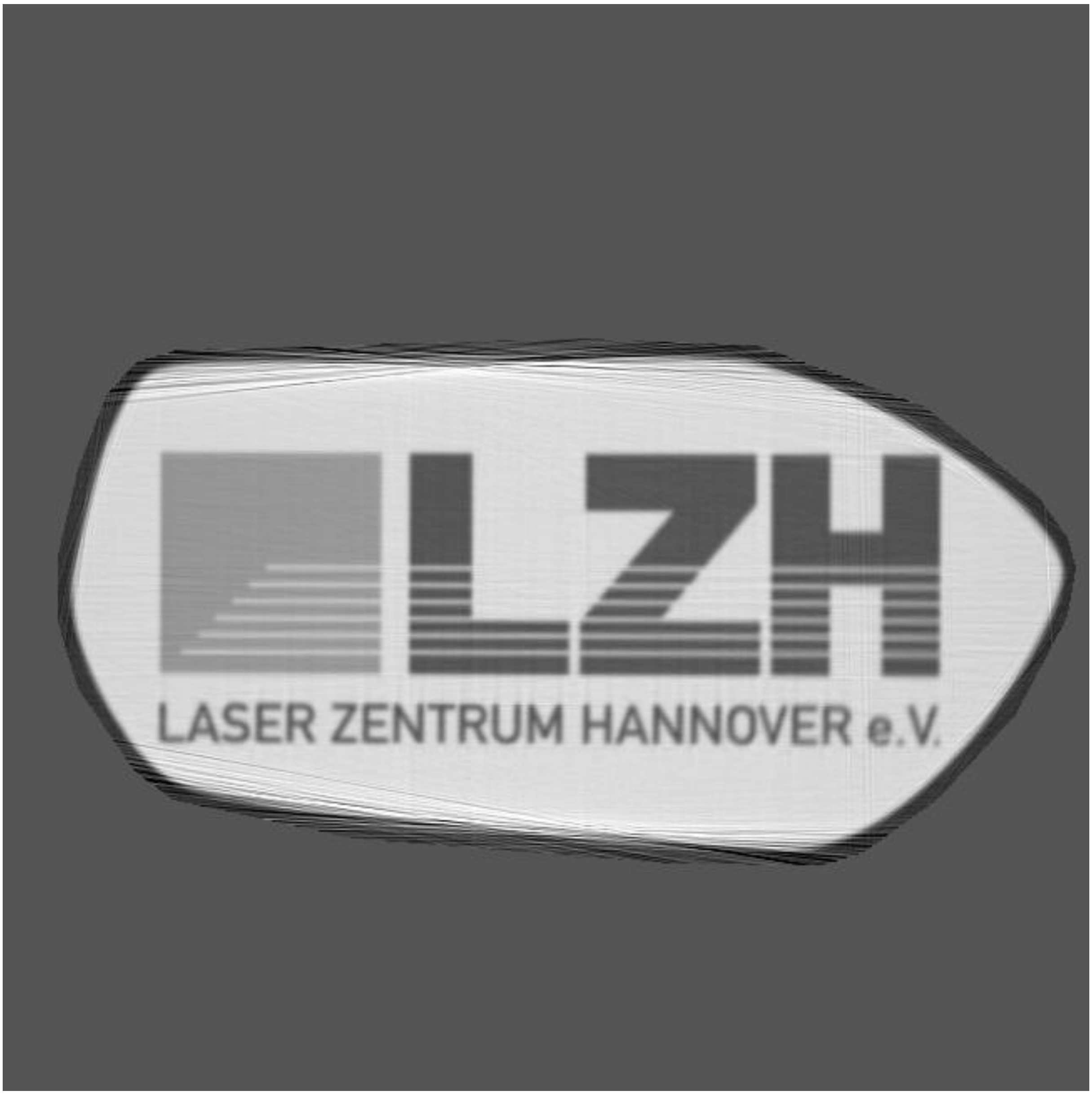
LZH
LASER ZENTRUM HANNOVER e.V.

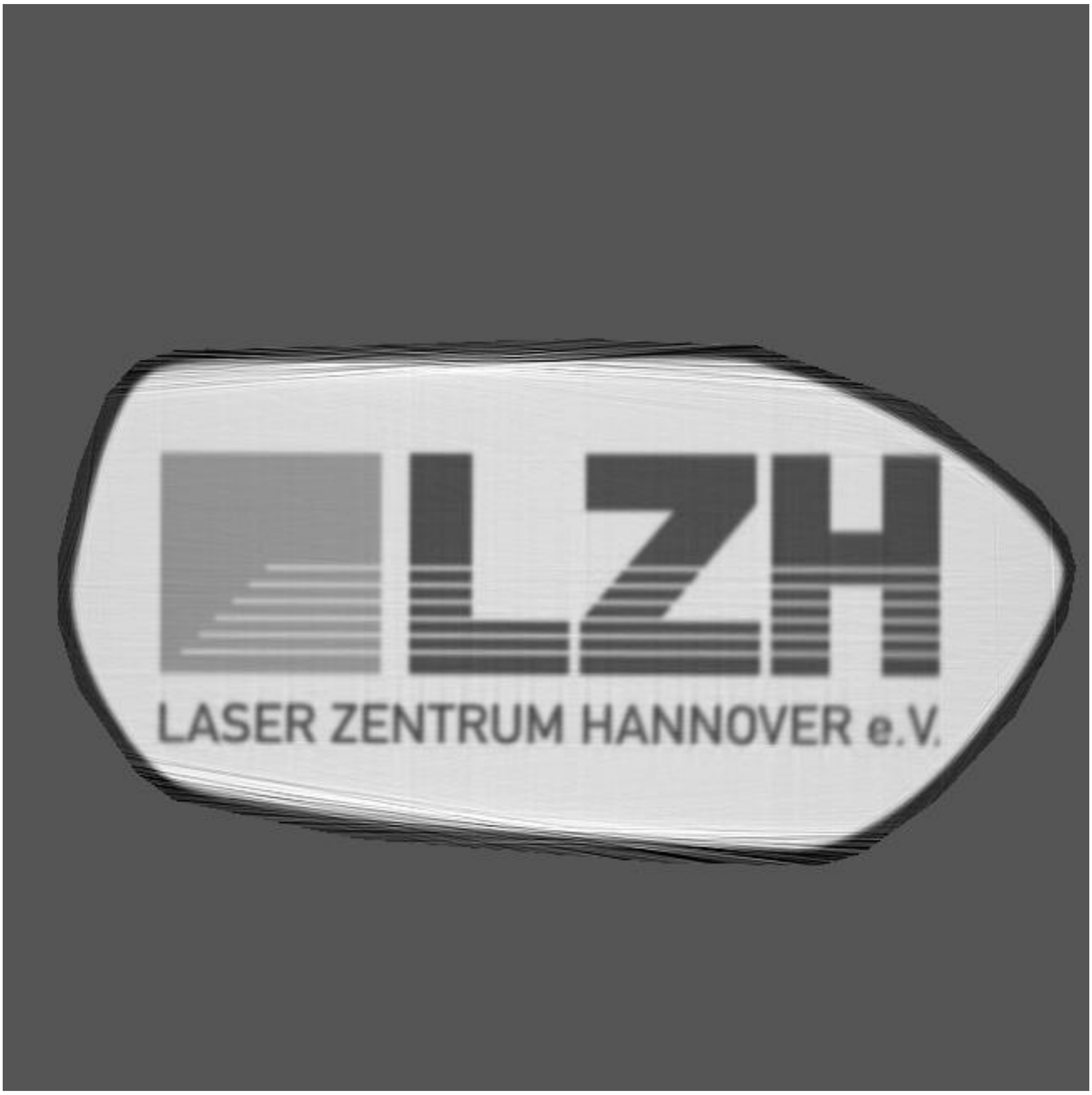
LZH
LASER ZENTRUM HANNOVER e.V.

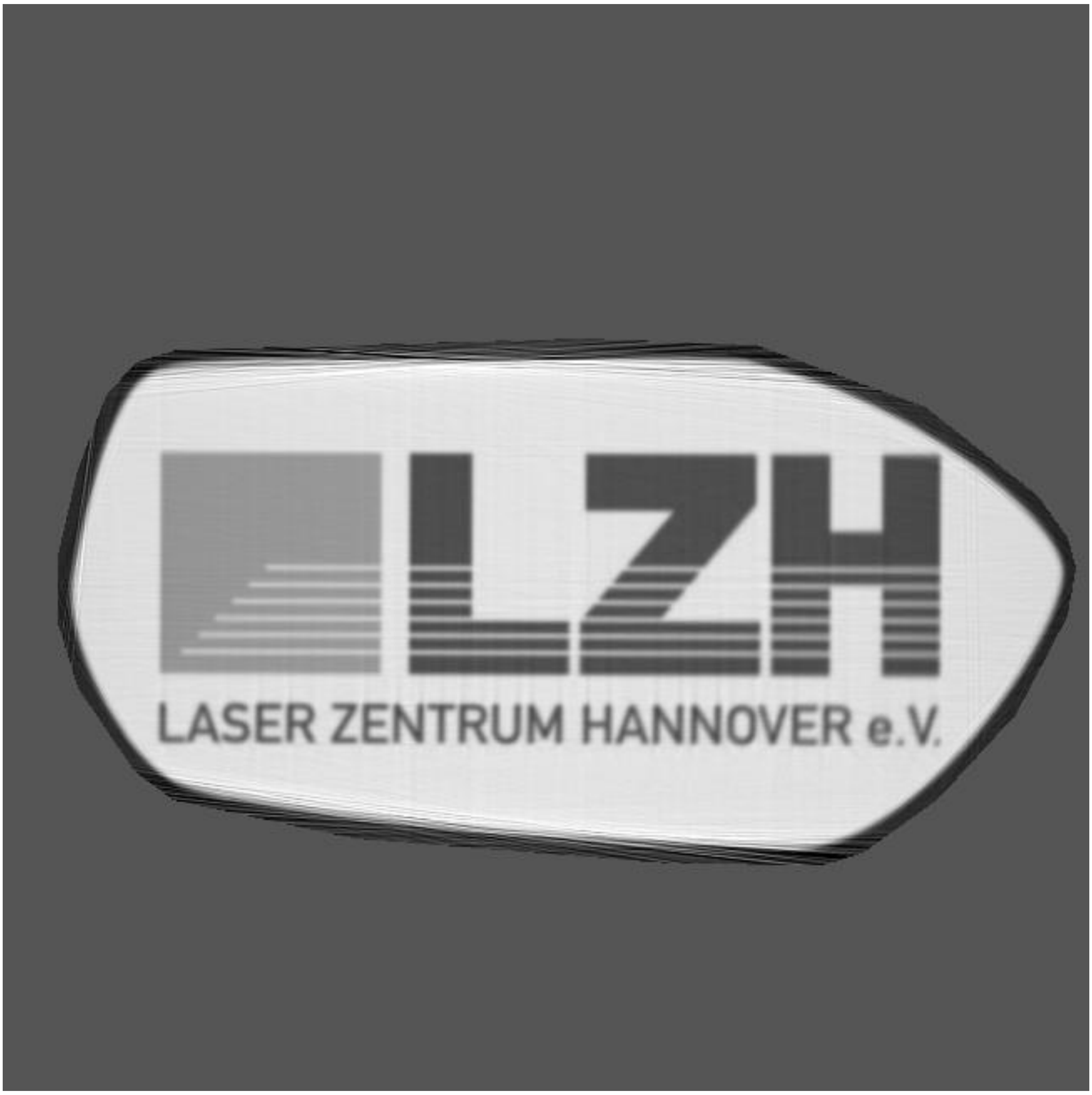
LZH
LASER ZENTRUM HANNOVER e.V.

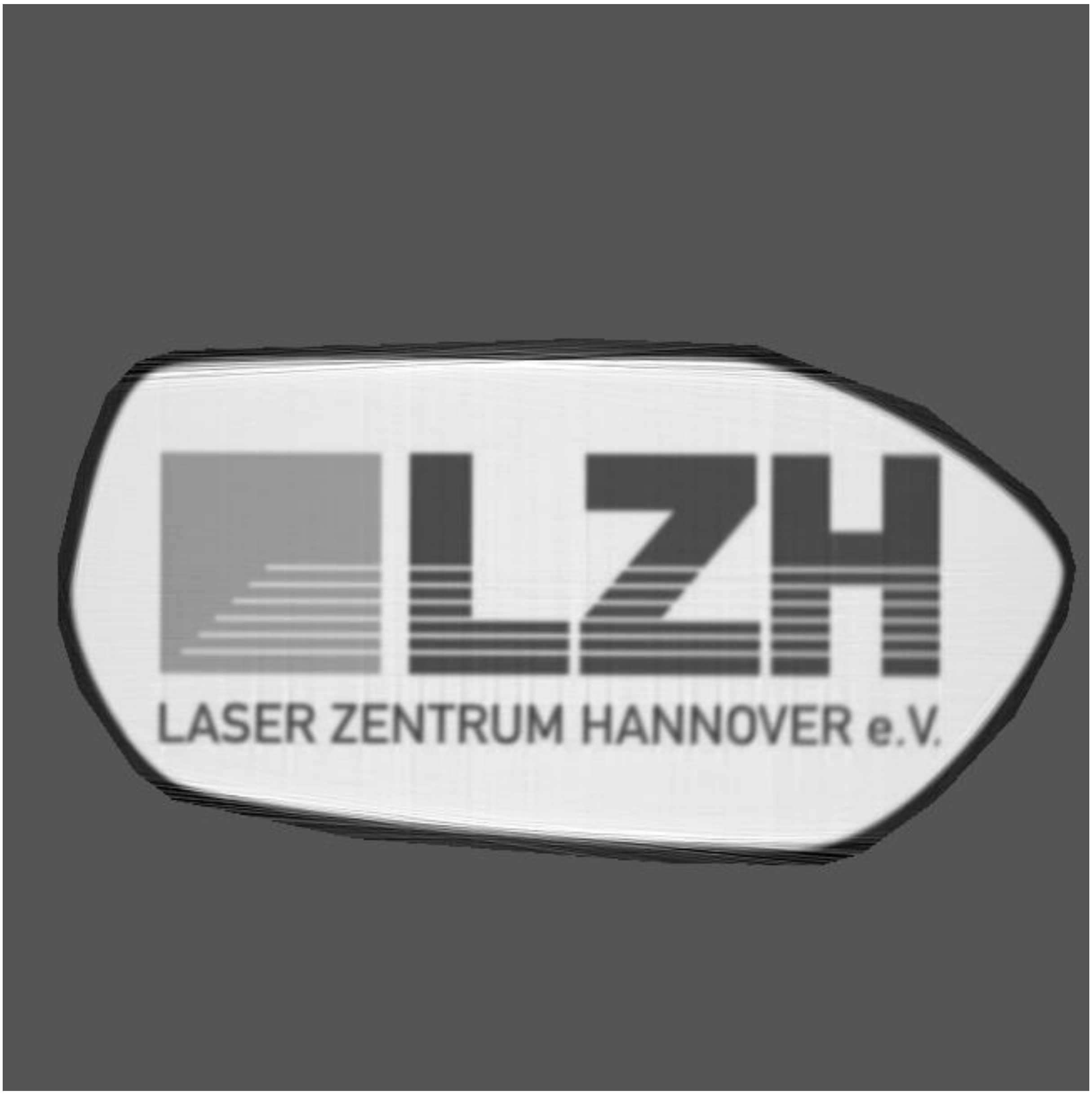
LZH
LASER ZENTRUM HANNOVER e.V.

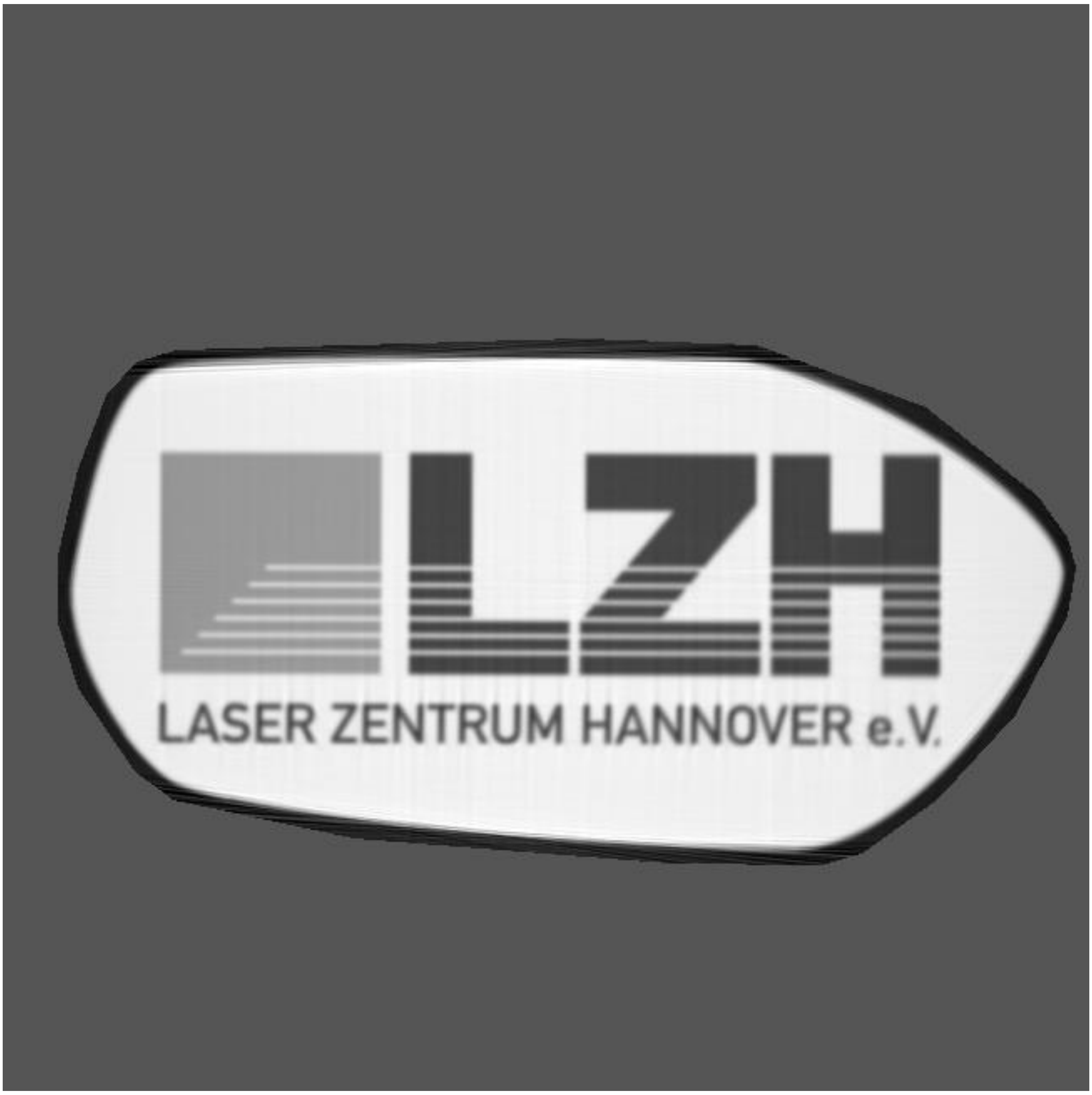
LZH
LASER ZENTRUM HANNOVER e.V.

LZH

LASER ZENTRUM HANNOVER e.V.

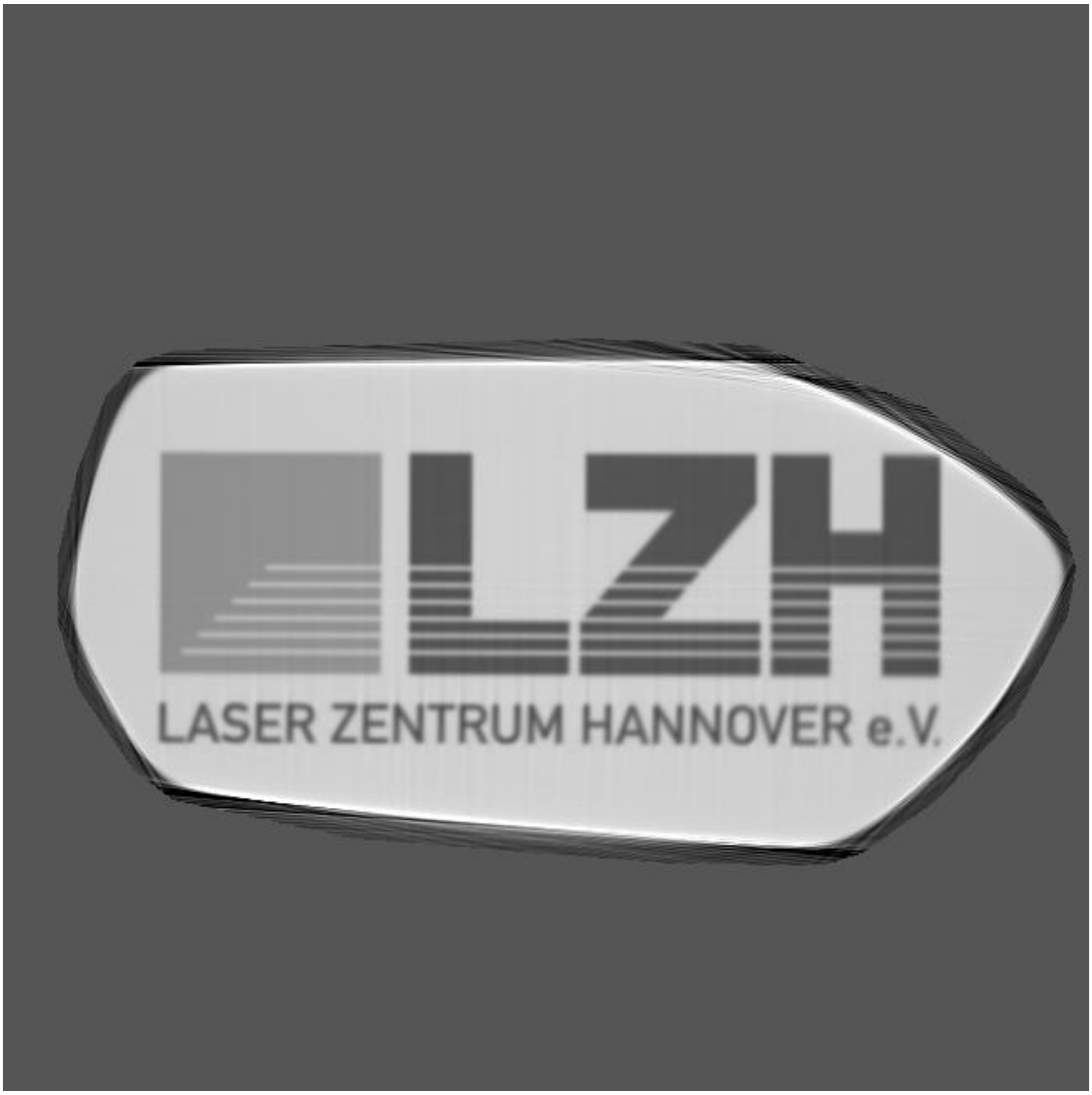
LZH
LASER ZENTRUM HANNOVER e.V.

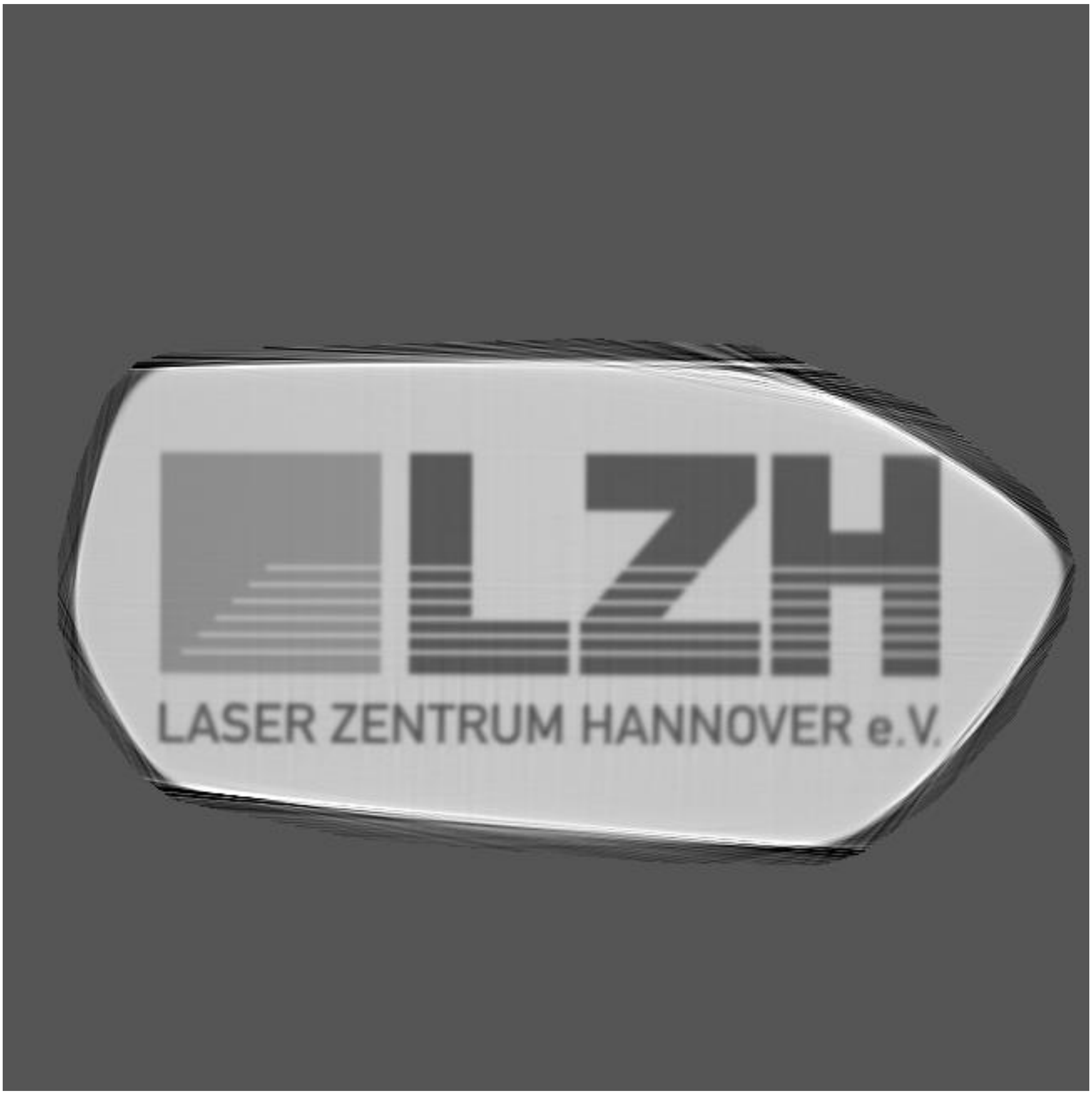
LZH
LASER ZENTRUM HANNOVER e.V.

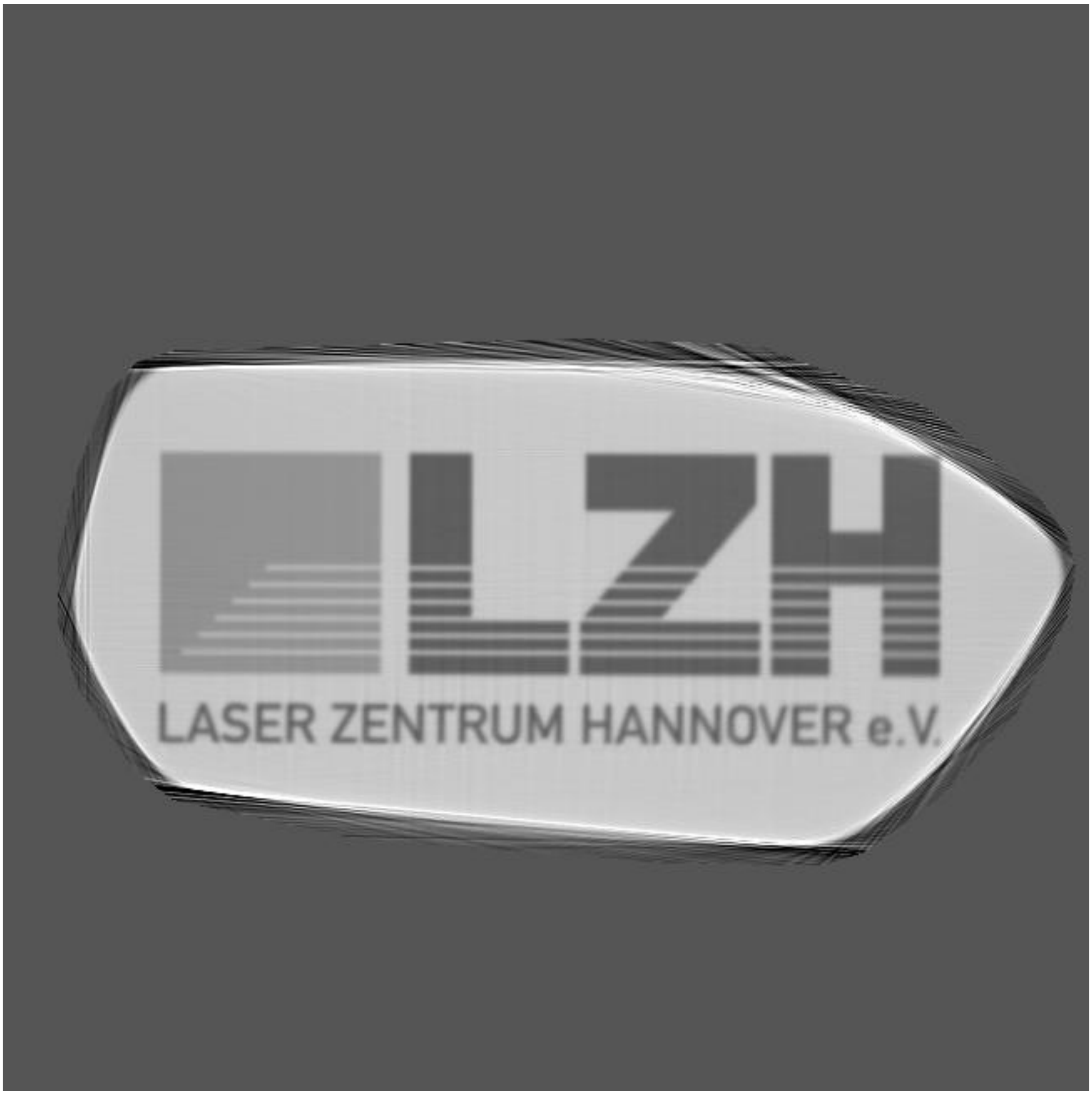
LZH
LASER ZENTRUM HANNOVER e.V.

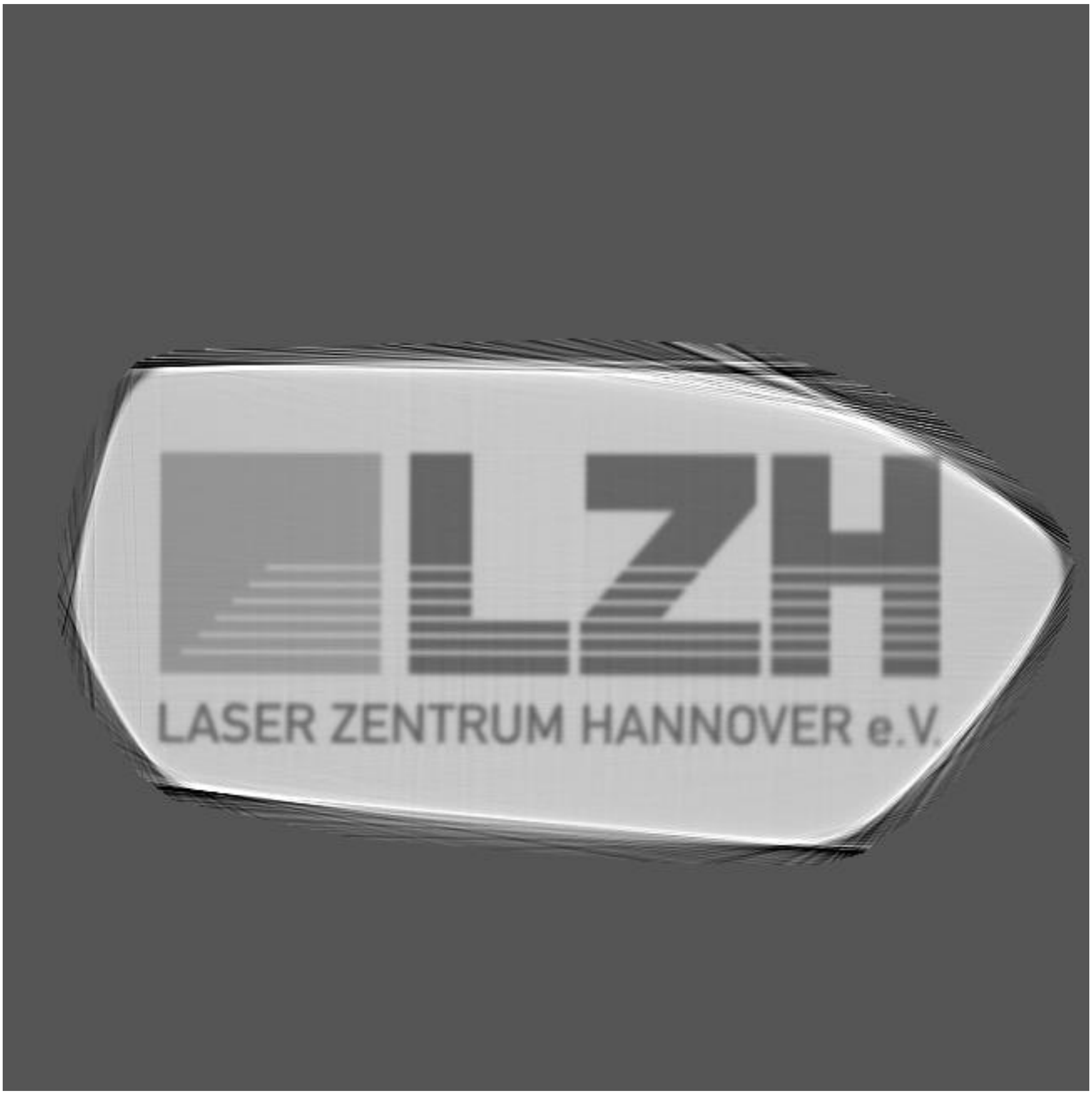
LZH
LASER ZENTRUM HANNOVER e.V.

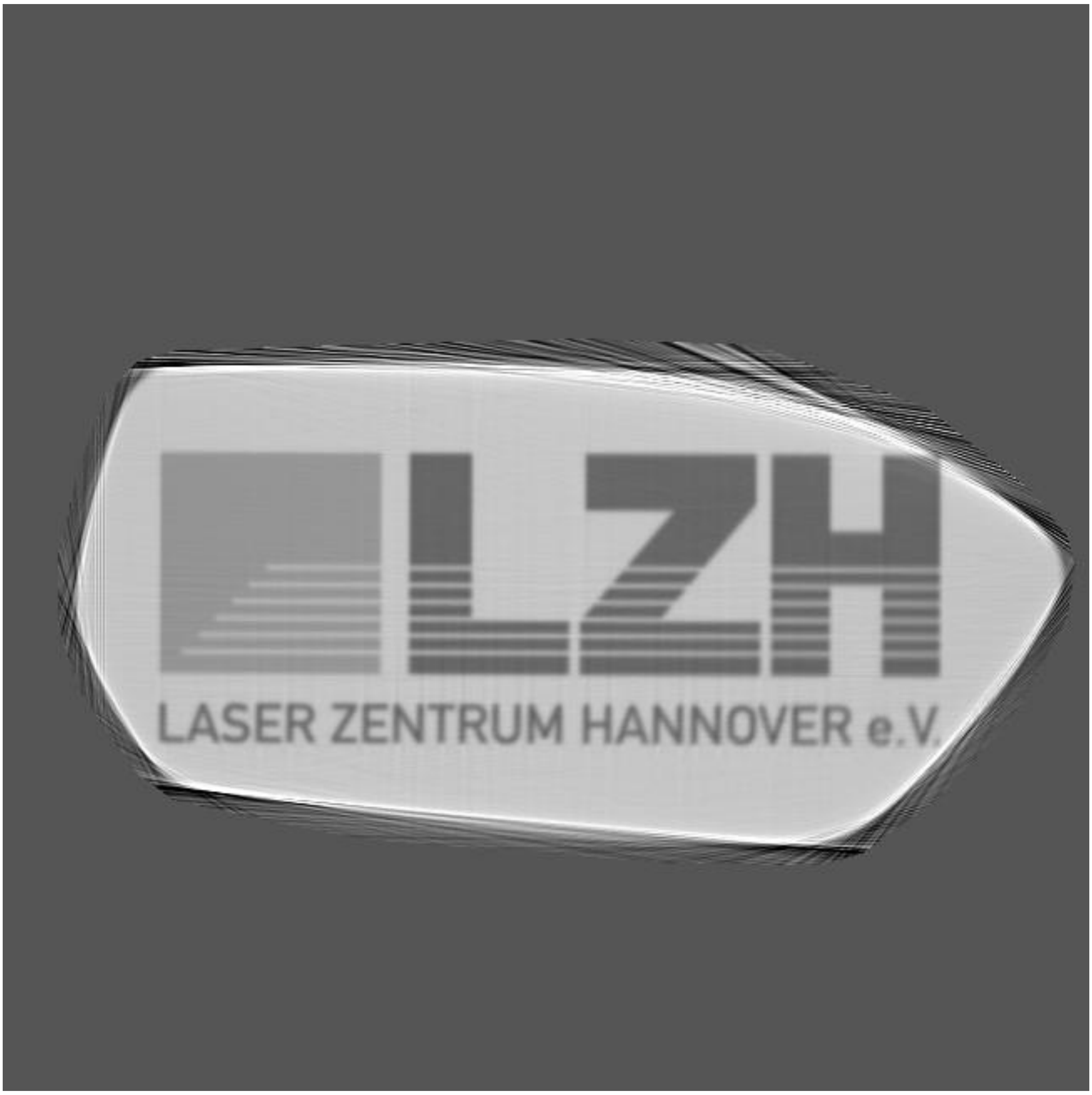
LZH
LASER ZENTRUM HANNOVER e.V.

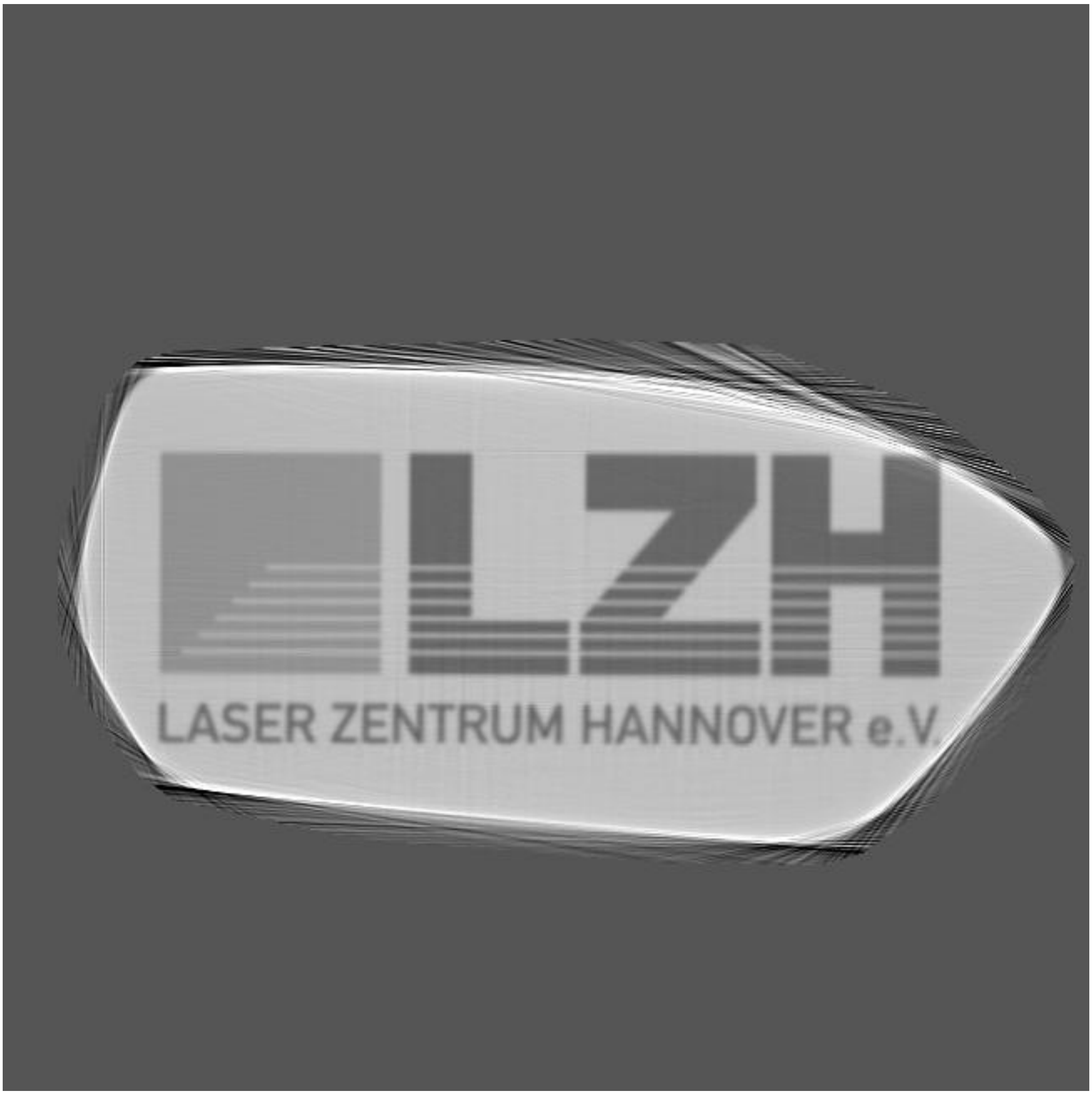
LZH
LASER ZENTRUM HANNOVER e.V.

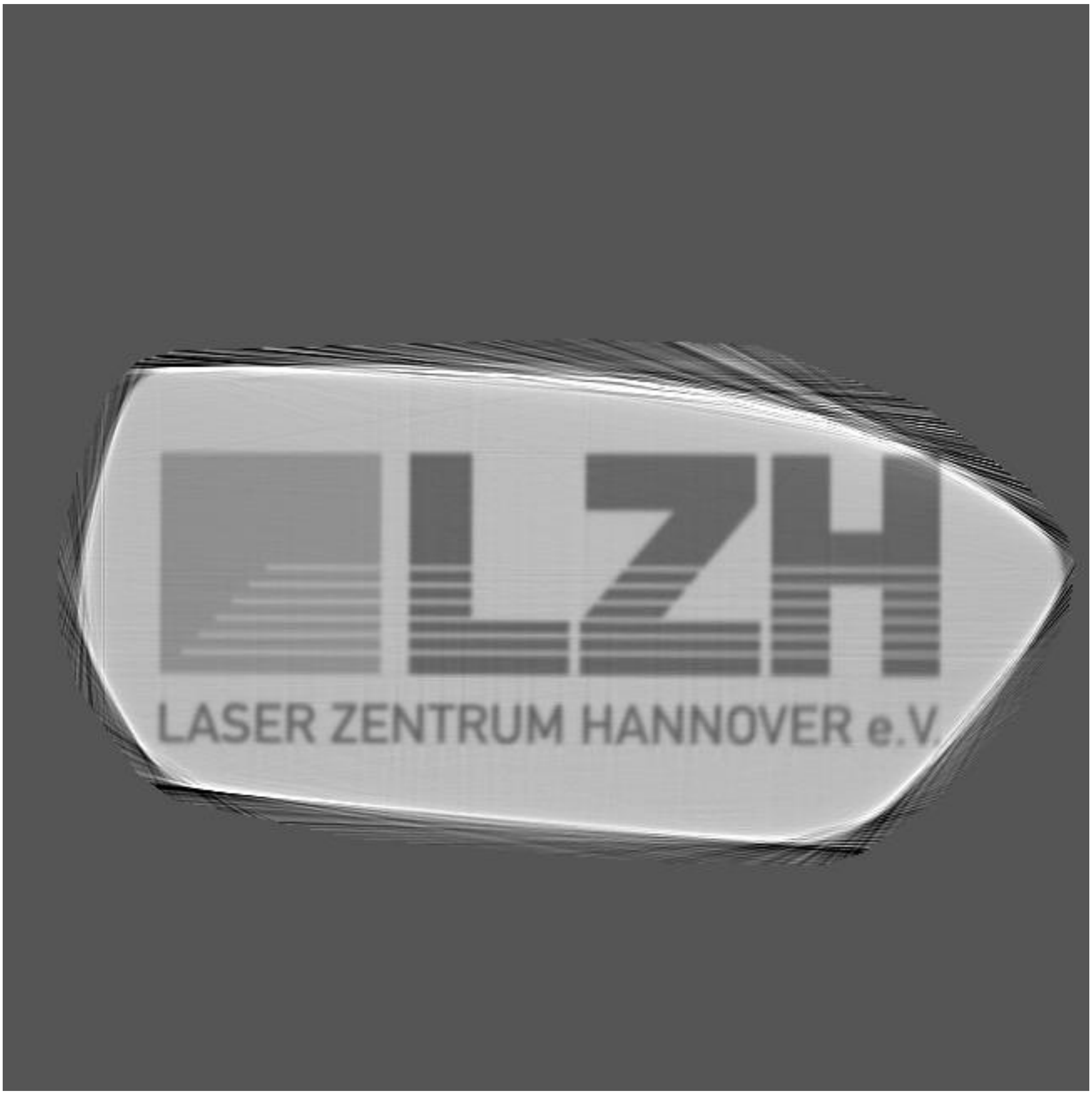
LZH
LASER ZENTRUM HANNOVER e.V.

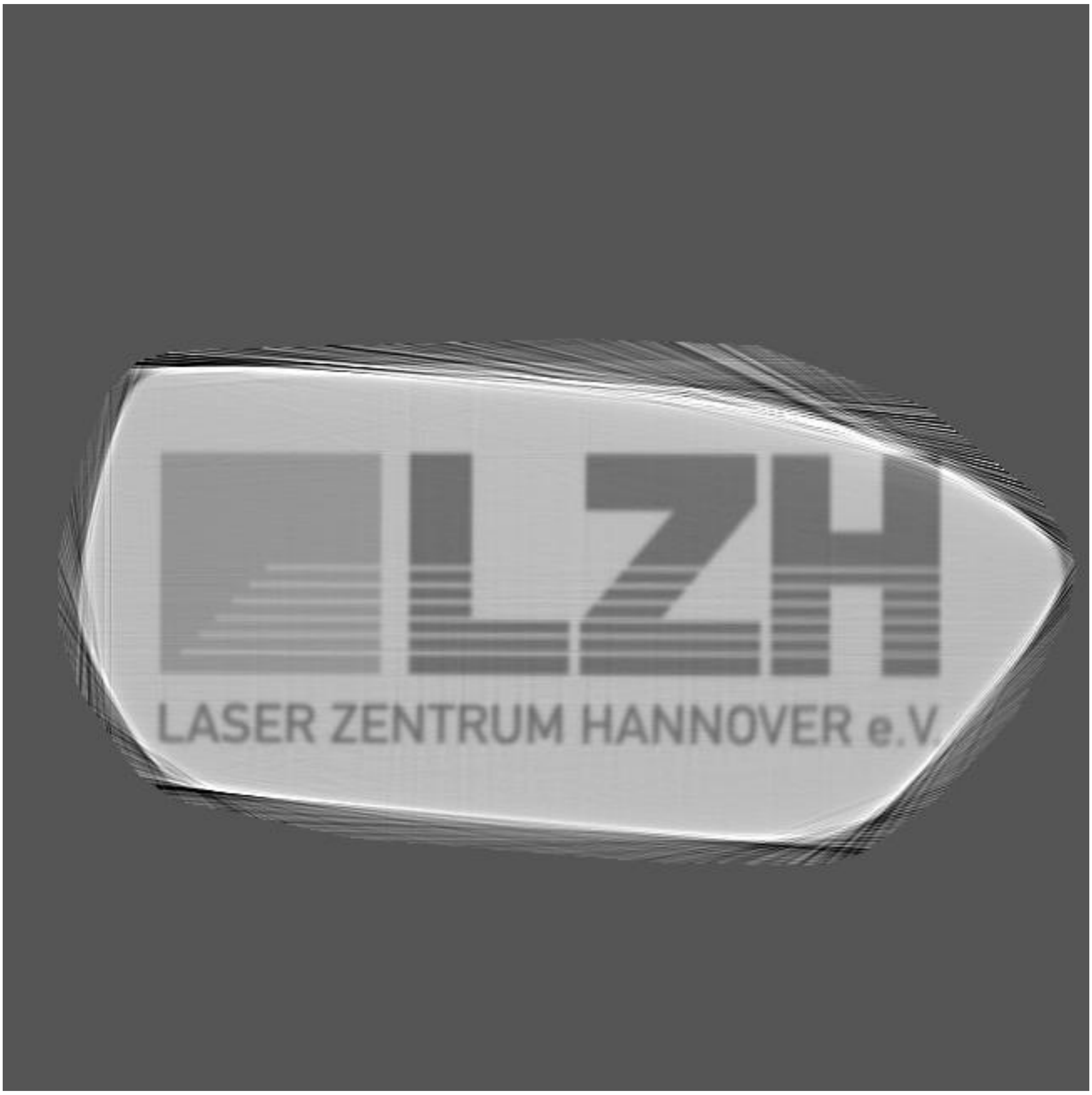
LZH
LASER ZENTRUM HANNOVER e.V.

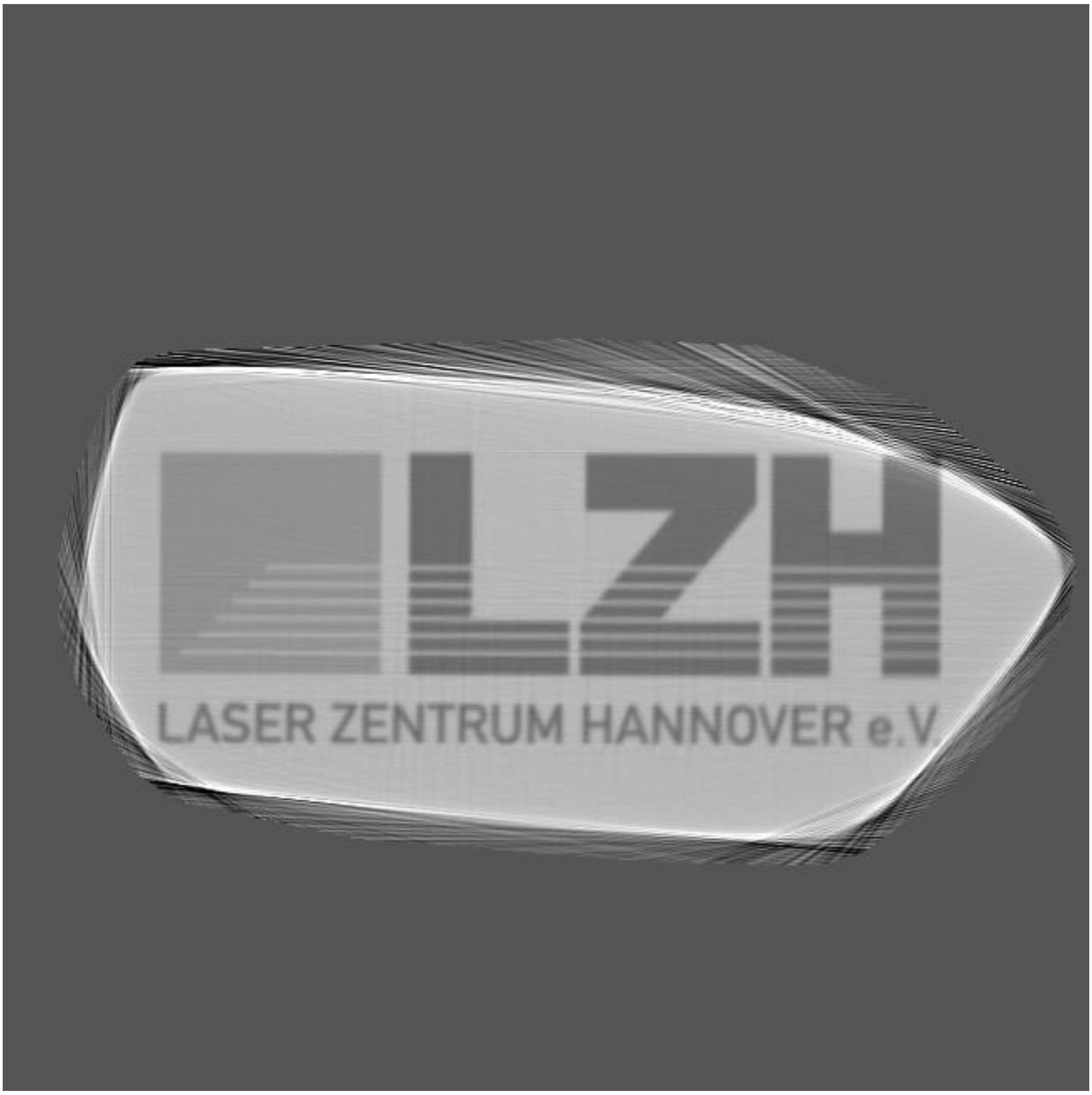
LZH
LASER ZENTRUM HANNOVER e.V.

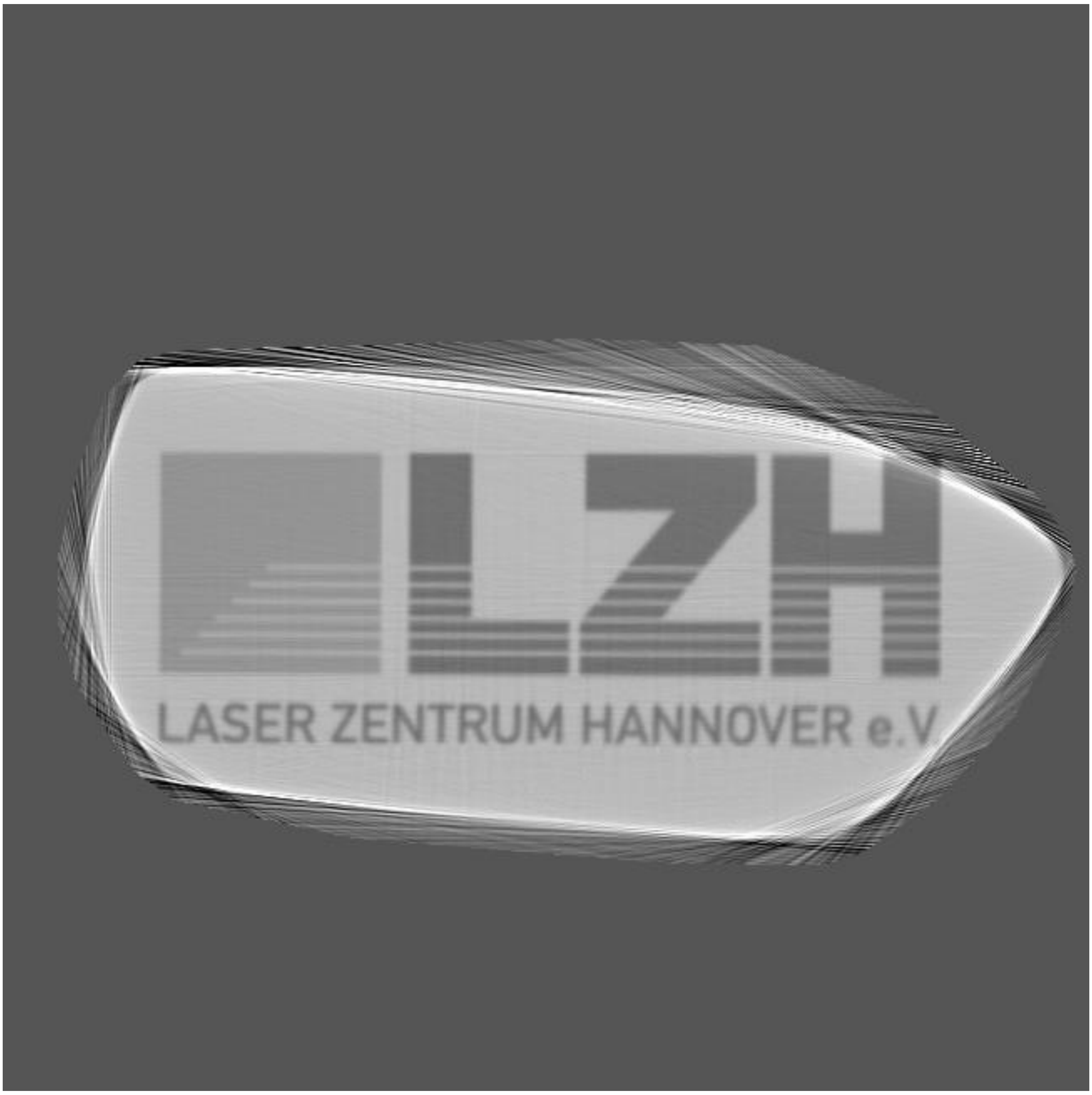
LZH
LASER ZENTRUM HANNOVER e.V.

SSIM and APD analysis of the previous images (also available on github under data\Lzh logo SSIM and APD analysis.xlsx):

Uncorrected simulation, entire image:

Medium RI	SSIM		Medium RI	APD	
	Value	Standard deviation		Value	Standard deviation
1.3	0.326	0.306	1.3	74.851	52.162
1.31	0.349	0.315	1.31	72.123	50.873
1.32	0.388	0.329	1.32	69.24	49.56
1.33	0.416	0.337	1.33	65.804	48.052
1.34	0.446	0.343	1.34	63.083	46.952
1.35	0.497	0.348	1.35	58.585	45.41
1.36	0.547	0.346	1.36	52.206	42.966
1.37	0.612	0.336	1.37	44.648	40.086
1.38	0.708	0.301	1.38	37.378	34.761
1.39	0.826	0.235	1.39	27.406	25.974
1.4	1	0	1.4	0	0
1.41	0.845	0.246	1.41	22.032	20.758
1.42	0.752	0.309	1.42	37.178	25.606
1.43	0.69	0.342	1.43	48.702	29.524
1.44	0.641	0.355	1.44	64.053	34.565
1.45	0.612	0.366	1.45	67.639	36.375
1.46	0.592	0.371	1.46	71.89	37.962
1.47	0.579	0.374	1.47	72.835	38.338
1.48	0.563	0.372	1.48	77.415	40.59
1.49	0.554	0.371	1.49	78.352	40.953
1.5	0.543	0.367	1.5	81.239	42.192

Uncorrected simulation, logo region:

Medium RI	SSIM		Medium RI	APD	
	Value	Standard deviation		Value	Standard deviation
1.3	0.375	0.34	1.3	53.075	34.84
1.31	0.401	0.348	1.31	51.512	33.83
1.32	0.439	0.355	1.32	49.86	32.681
1.33	0.472	0.362	1.33	47.868	31.367
1.34	0.509	0.364	1.34	46.114	29.856
1.35	0.556	0.361	1.35	43.032	28.012
1.36	0.608	0.345	1.36	38.601	25.647
1.37	0.674	0.316	1.37	33.1	22.557
1.38	0.762	0.261	1.38	27.832	18.289
1.39	0.876	0.155	1.39	20.627	12.498
1.4	1	0	1.4	0	0
1.41	0.879	0.156	1.41	17.606	12.349
1.42	0.769	0.253	1.42	31.006	20.479
1.43	0.694	0.303	1.43	41.146	26.718
1.44	0.637	0.325	1.44	54.398	34.873
1.45	0.604	0.34	1.45	57.683	37.014
1.46	0.582	0.346	1.46	61.304	39.067
1.47	0.567	0.349	1.47	62.138	39.217
1.48	0.552	0.346	1.48	66.044	42.228
1.49	0.542	0.344	1.49	66.782	42.478
1.5	0.532	0.341	1.5	69.147	44.094

Corrected simulation, entire image:

Medium RI	SSIM		Medium RI	APD	
	Value	Standard deviation		Value	Standard deviation
1.3	0.629	0.31	1.3	39.707	24.761
1.31	0.656	0.306	1.31	37.766	23.41
1.32	0.684	0.301	1.32	32.613	21.548
1.33	0.721	0.295	1.33	31.504	20.556
1.34	0.747	0.292	1.34	30.506	19.878
1.35	0.773	0.281	1.35	26.77	18.262
1.36	0.818	0.263	1.36	24.623	16.275
1.37	0.848	0.248	1.37	19.961	14.062
1.38	0.886	0.21	1.38	15.998	11.575
1.39	0.932	0.154	1.39	8.867	8.002
1.4	1	0	1.4	0	0
1.41	0.849	0.252	1.41	32.158	18.852
1.42	0.809	0.277	1.42	39.281	21.948
1.43	0.782	0.295	1.43	39.571	23.724
1.44	0.758	0.3	1.44	41.547	25.628
1.45	0.737	0.312	1.45	42.616	27.692
1.46	0.712	0.315	1.46	46.842	29.243
1.47	0.698	0.317	1.47	49.797	30.387
1.48	0.675	0.319	1.48	50.893	31.035
1.49	0.657	0.323	1.49	53.613	32.346
1.5	0.637	0.329	1.5	53.758	33.256

Corrected simulation, logo region:

Medium RI	SSIM		Medium RI	APD		Medium RI
	Value	Standard deviation		Value	Standard deviation	
1.3	0.768	0.148	1.3	31.718	17.288	1.3
1.31	0.79	0.138	1.31	30.266	16.464	1.31
1.32	0.816	0.119	1.32	26.465	14.478	1.32
1.33	0.846	0.098	1.33	25.297	13.627	1.33
1.34	0.867	0.086	1.34	24.479	13.186	1.34
1.35	0.884	0.076	1.35	21.757	11.846	1.35
1.36	0.919	0.051	1.36	19.724	10.386	1.36
1.37	0.937	0.041	1.37	16.279	8.725	1.37
1.38	0.953	0.031	1.38	13.494	7.058	1.38
1.39	0.975	0.019	1.39	7.913	4.149	1.39
1.4	1	0	1.4	0	0	1.4
1.41	0.926	0.058	1.41	25.836	13.676	1.41
1.42	0.895	0.079	1.42	31.593	16.779	1.42
1.43	0.877	0.089	1.43	31.727	17.355	1.43
1.44	0.855	0.103	1.44	33.367	18.956	1.44
1.45	0.844	0.111	1.45	34.017	19.463	1.45
1.46	0.82	0.129	1.46	37.217	20.976	1.46
1.47	0.809	0.134	1.47	39.365	21.783	1.47
1.48	0.778	0.16	1.48	40.574	23.624	1.48
1.49	0.77	0.161	1.49	42.356	23.923	1.49
1.5	0.755	0.174	1.5	42.436	24.818	1.5
